# A fourth-order C-bracket scheme for nonlinear shallow-water sloshing in an oscillating tank with non-periodic inflow-outflow boundary conditions and biharmonic dissipation

**DOI:** 10.1007/s00332-026-10317-0

**Published:** 2026-08-14

**Authors:** Hamid Alemi Ardakani

**Affiliations:** Department of Mathematics and Statistics, University of Exeter, Exeter, EX4 4QF UK

**Keywords:** High-order Poisson-bracket discretisation, Arakawa C-grid, Biharmonic dissipation, Shallow-water sloshing, Discrete conservation laws, Non-periodic inflow-outflow boundary conditions, 65M06, 65P10, 70G45, 70H25, 76M30

## Abstract

The second-order energy- and potential-enstrophy-conserving numerical scheme introduced by Arakawa & Lamb (1981) for the shallow-water equations with *periodic* boundary conditions, and extended by Salmon (2004) in the context of Hamiltonian Poisson-bracket discretisation, is further extended to a *fourth-order discretisation* for the problem of *nonlinear shallow-water sloshing* over a *corrugated bottom surface* in a rectangular rigid basin, with *symmetric and non-symmetric porous solid side walls* and *periodic and non-periodic prescribed inflow-outflow boundary conditions*, undergoing a prescribed *coupled surge-sway motion*. Adaptation to a finite domain with periodic and non-periodic inflow-outflow boundary conditions requires a new approach to the boundary conditions at *porous* solid boundaries, and the ghost cell grid point approximations in the context of the fourth-order finite-difference discretisation on the Arakawa C-grid. Theoretical Poisson-bracket arguments are used to define the required boundary conditions. In addition, standard *biharmonic dissipation* is incorporated into the numerical modelling to prevent potential enstrophy from accumulating at the smallest resolved scales, which improves the quality and stability of the approximate solutions, enabling long-term integrations to be carried out. The scheme is implemented, shown to preserve the total mass, energy, and potential enstrophy over *long-time* integration with bounded fluctuations. The presented *higher-order C-bracket sloshing integrator* provides a robust, stable, fast and precise building block for long-time computational modelling of floating ocean wave energy devices with *flexible* components, like a flexible bottom surface or membrane for wave energy extraction.

## Introduction

To improve the simulation of nonlinear aspects of the flow over steep topography, Arakawa and Lamb (1981) derived an energy- and potential-enstrophy-conserving difference scheme for the shallow-water equations (SWEs), that apart from errors associated with finite time step, exactly conserves finite-difference analogues of the energy and potential enstrophy. Simultaneous conservation of energy and potential enstrophy is particularly important because it prevents the spurious cascade of energy into high wavenumbers (Salmon 2004; Stewart and Dellar 2016; Arakawa and Lamb 1981). Cascades of energy and potential enstrophy are two important features of two-dimensional incompressible turbulent flows, see e.g., (Kraichnan 1967; Lilly 1971), where the energy transfers to lower wave numbers and the enstrophy transfers to higher wave numbers (Brecht et al. 2021). Hence, energy conserving and potential-enstrophy dissipating numerical schemes are also desirable to prevent potential enstrophy from accumulating at the grid-scale, which improves the stability of the numerical scheme over *long-time* integration.

Based on the anticipated potential vorticity method (Sadourny and Basdevant 1985), which is closely related to Lax–Wendroff advection schemes (McRae and Cotter 2014; Arakawa and Hsu 1990) developed a family of finite-difference schemes to incorporate potential enstrophy dissipation into discrete shallow water equations while energy is conserved. The anticipated potential vorticity method has been widely used in the literature, see e.g., (Ringler et al. 2008, 2010; McRae and Cotter 2014) and references therein, to dissipate potential enstrophy at small scales. More recently, Gay-Balmaz and Holm (2013) developed the continuous selective decay framework, in which a Casimir of the ideal formulation, i.e. enstrophy in two-dimensional flows and helicity in three-dimensional flows, decays in time while the energy is conserved. The theory of the selective decay uses the Lie–Poisson structure of the ideal theory. Brecht et al. (2021) used the variational discretisation of Pavlov et al. (2011) in combination with the continuous selective decay theory of Gay-Balmaz and Holm (2013) to develop the discrete version of the selective decay framework. This method is applied to the shallow-water equations to dissipate the potential enstrophy, which improves the quality of the numerical solutions over long-time integration. The selective decay framework is related to the numerical method of anticipated potential vorticity discussed in Sadourny and Basdevant (1981) (see also Gay-Balmaz and Holm (2013)). In the present paper, we start with an energy- and potential-enstrophy-conserving model and then introduce a *biharmonic dissipating term*, which gives us complete control over the enstrophy dynamics in the model.

Salmon (2004) rederived and generalised the 1981 algorithm of Arakawa and Lamb (1981) by writing the shallow-water equations in Hamiltonian Poisson-bracket form, and replacing the Hamiltonian functional and the Poisson-bracket operator by finite-difference approximations that maintain the desired conservation laws. In particular, Salmon (2004) discussed how the second-order Arakawa–Lamb type schemes can be extended to the fourth-order Takano–Wurtele type schemes (Takano and Wurtele 1982) (fourth-order in the vorticity equation only) on the Arakawa C-grid using Poisson-bracket arguments. Stewart and Dellar (2016) extended the second-order non-canonical Poisson-bracket discretisation of Salmon (2004) to derive an energy- and potential-enstrophy-conserving finite-difference scheme for the non-traditional single layer and multi-layer shallow-water equations that include the complete Coriolis force and topography. The schemes of Arakawa and Lamb (1981), Salmon (2004), and Stewart and Dellar (2016) are applied to the shallow-water equations in domains with boundaries and *periodic boundary conditions*.

Incorporation of *solid-wall boundary conditions* into the shallow-water Poisson bracket schemes on the staggered Arakawa C-grid has not been straightforward, since potential vorticity should be specified along the domain boundaries. In addition, incorporation of *prescribed non-periodic inflow-outflow boundary conditions* into the shallow-water C-bracket schemes for nonlinear sloshing problems has not been discussed in the literature. Hence, a principal aim in the current paper is to adapt *higher-order C-bracket schemes* to shallow-water sloshing problems which preserve potential-enstrophy, total mass and energy in the presence of *non-symmetric rigid porous boundaries with non-periodic inflow-outflow boundary conditions*.

In the present study, porous boundaries are modeled as symmetric or asymmetric inlet and outlet openings distributed along the solid side walls, allowing *prescribed* periodic or non-periodic inflow and outflow boundary conditions to be imposed. Fluid transport through the side walls is therefore not modeled explicitly, and neither Darcy’s law nor pressure-drop transmission conditions are considered, in contrast to the approaches adopted, e.g., in Laws and Livesey (1978), Molin (2001), Straughan (2008), Behera et al. (2013), Chen et al. (2019).

There are a few papers in the literature that are devoted to finite-difference bracket schemes for the shallow-water equations that conserve energy and potential enstrophy in the presence of boundaries. Salmon (2007), Salmon (2009) used a Nambu bracket formulation (with the vorticity, divergence and wave height as the field variables) on a *standard grid*. Ketefian and Jacobson (2009) derived a boundary scheme which extends the Arakawa–Lamb scheme on the Arakawa C grid to model fluid-land boundaries of arbitrary shape in inviscid shallow water flows. Their scheme uses stairsteps to approximate boundary profiles. Furthermore, Ketefian and Jacobson (2011) derived a numerical scheme for treating fluid-land boundaries in inviscid shallow water flows that approximates boundary profiles with piecewise linear segments while conserving the domain-summed mass, energy, vorticity, and potential enstrophy. This scheme is an extension of the boundary scheme presented in Ketefian and Jacobson (2009).

Alemi Ardakani and Bridges (2024) adapted the *second-order* finite-difference Poisson-bracket scheme of Salmon (2004) and Stewart and Dellar (2016) to the problem of *shallow-water sloshing* over a variable bottom surface in a rectangular basin bounded by solid walls, undergoing a prescribed forcing function in the horizontal plane (prescribed surge-sway motions), with *sloshing boundary conditions*. They argued in favour of a *zero-potential-vorticity condition* at a rigid-wall boundary, which is consistent with the material conservation of the potential vorticity satisfied by the shallow-water equations, and the fact that potential enstrophy is a Casimir of the shallow-water Poisson bracket. The principal motivation in Alemi Ardakani and Bridges (2024) was the design of structure-preserving algorithms for *long-time* simulation of *ocean wave energy converters* [49].

In particular, we are interested in robust, stable, fast and precise *structure-preserving* integrators for long-time computational modelling of a new class of ducted wave energy converters (WEC) with a *flexible* bottom surface, named *FlexSlosh WEC* [50]. It is of interest to preserve the total energy in the numerical scheme, but also to maintain the correct partition of energy between different components of the dynamical system, e.g., the partition of energy between the WEC’s interior fluid sloshing and the flexible bottom topography. Structure-preserving numerical schemes are particularly useful for long-time numerical modelling and power take-off optimisation of wave energy devices. These methods have not been applied to computational modelling of ocean wave energy extraction.

**Fig. 1:**
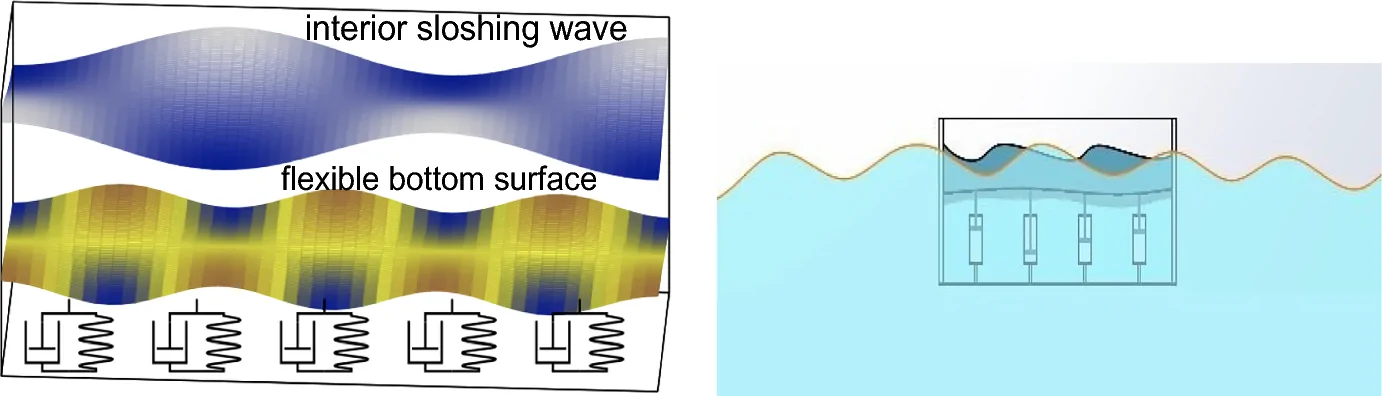
Schematic of the FlexSlosh wave energy converter. On the left, the schematic shows a rectangular basin with interior shallow water sloshing over a flexible bottom surface that is mechanically connected to a group of spring/dashpot system as its power take-off (PTO) system. On the right, the FlexSlosh WEC is in hydrodynamic interaction with ocean surface waves. The flexible bottom surface is mechanically connected to single or double action hydraulic pumps, which dissipate or transform the energy of interior fluid sloshing through the deflection of the bottom topography.

The FlexSlosh WEC is a *freely* floating rigid-body, with porous solid side walls and a synthetic flexible bottom surface installed inside and along the bottom of the rigid-body, which is partially filled with shallow water and is in hydrodynamic interaction with exterior ocean surface waves. It extracts energy from the coupling between the interior shallow-water sloshing and the the *flexible* bottom topography. The flexible bottom is mechanically connected to a mesh/group of spring/dashpot system, as its power take-off (PTO) system, e.g., single or double action hydraulic pumps, which dissipate or transform the energy of interior fluid sloshing through the deflection of the bottom topography. The schematic of the FlexSlosh WEC is illustrated in Fig. 1. The FlexSlosh WEC can be divided into multiple compartments which makes the design of the flexible bottom surface and its energy extraction system more efficient. The inlets/outlets can be located along the rigid side walls such that the WEC’s influx/efflux flow rates would not affect the interior fluid sloshing dynamics. In addition, through appropriate adjustment of influx/efflux flow rates, one has control over the total mass in the FlexSlosh WEC, and so the shallowness parameter for optimal sloshing height and interior sloshing energy extraction.

To provide a high-resolution, stable, accurate and robust building block for long-time simulation of the sloshing problem in complex dynamical systems such as the FlexSlosh WEC, the aim in this paper is threefold:

(i) To improve the simulation of nonlinear aspects of the shallow-water sloshing over a *corrugated bottom surface*, the second-order C-bracket *sloshing integrator* of Alemi Ardakani and Bridges (2024) is extended to a *fourth-order* accurate integrator (in the grid spacing) in the potential vorticity dependent component of the shallow-water Poisson bracket (1.15). We note that a fourth-order energy- and potential-enstrophy-conserving difference scheme was first designed by Takano and Wurtele (1982). This scheme can be recovered from the generalised non-canonical Poisson-bracket C-grid discretisations of Salmon (2004). To the best of our knowledge, the fourth-order C-bracket difference scheme is only used in Salmon (2004) for a numerical simulation with *periodic boundary conditions* and an initial condition for parallel shear layers slightly perturbed by a sinusoidal cross flow (see figure 6 in Salmon (2004)). In the current paper, we first adapt *the fourth-order C-bracket scheme* to the problem of *shallow-water sloshing over a variable bottom topography* in a rigid rectangular tank undergoing a *prescribed translational motion* in the horizontal plane. This requires extension of the sloshing boundary conditions presented in Alemi Ardakani and Bridges (2024).
(ii) The FlexSlosh WEC can have *symmetric* or *non-symmetric porous solid side walls*, and so to model the associated *periodic and/or non-periodic inflow-outflow boundary conditions*, the fourth-order C-bracket sloshing integrator in part (i) is extended to include inflow-outflow boundary conditions in the context of the fourth-order Poisson-bracket discretisation on the Arakawa C-grid. This requires specification of the flow field variables for the ghost cell grid points, and the potential vorticity on the boundary grid points and for the ghost cell grid points such that the Casimir properties of the shallow-water Poisson-bracket are preserved. Derivation of the inflow-outflow boundary conditions, using theoretical Poisson-bracket arguments, is given in §3.
(iii) The potential enstrophy accumulation at the grid-scale should be dissipated to damp the small scale noise, and thus improve the quality of the numerical solutions and obtain physically relevant solutions. In addition, a linear fourth-order diffusion (biharmonic dissipation), with a second-order finite-difference discretisation on the C-grid, is applied to the velocity field in the shallow-water momentum equations to remove this small scale noise and improve the stability of the numerical scheme for long-time *large amplitude* numerical simulations.

The development of a *fourth-order* C-bracket scheme, accommodating symmetric or non-symmetric porous boundaries with associated periodic and/or non-periodic inflow-outflow boundary conditions in the numerical modelling, and adding standard biharmonic dissipation to the model to prevent potential enstrophy from accumulating at the smallest resolved scales are the new features of the present paper in relation to Alemi Ardakani and Bridges (2024).

For the shallow-water sloshing simulations in this paper, the fluid occupies a rectangular region with a single-valued free surface $h\left( x,y,t\right)$, that is

1.1 $$\begin{aligned} 0\le z\le h\left( x,y,t\right) ,\quad 0\le x\le L_1,\quad 0\le y\le L_2\,. \end{aligned}$$

The SWEs in two-horizontal space dimensions over a variable bottom surface $h_b\left( x,y\right)$ and with a prescribed translational forcing function $\boldsymbol{q}(t)=\left( q_1(t),q_2(t)\right)$ take the form

1.2 $$\begin{aligned} \widetilde{u}_t-\mathcal {V}hv+\Phi _x = 0\,,\quad \widetilde{v}_t+\mathcal {V}hu+\Phi _y = 0\,,\quad h_t+\left( hu\right) _x+\left( hv\right) _y = 0 \,, \end{aligned}$$

where the canonical velocity $\widetilde{\boldsymbol{u}}\left( \boldsymbol{x},t\right) =\left( \widetilde{u}\left( x,y,t\right) , \widetilde{v}\left( x,y,t\right) \right)$ is given by

1.3 $$\begin{aligned} \widetilde{u}\left( x,y,t\right) =u\left( x,y,t\right) +\dot{q}_1(t) \quad \text{ and }\quad \widetilde{v}\left( x,y,t\right) =v\left( x,y,t\right) +\dot{q}_2(t) \,, \end{aligned}$$

$\boldsymbol{u}=\left( u\left( x,y,t\right) ,v\left( x,y,t\right) \right)$ is the vertically averaged horizontal fluid velocity, $\dot{\boldsymbol{q}}(t)=\left( \dot{q}_1(t),\dot{q}_2(t)\right)$, $h\left( x,y,t\right)$ is the fluid depth relative to the bottom surface $h_b\left( x,y\right)$, and the potential vorticity $\mathcal {V}\left( x,y,t\right)$ and the Bernoulli potential $\Phi \left( x,y,t\right)$ are respectively

1.4 $$\begin{aligned} \mathcal {V}=\left( v_x-u_y\right) /h \quad \text{ and }\quad \Phi = \frac{1}{2}\left( u^2+v^2\right) +g\left( h+h_b\right) \,. \end{aligned}$$

A Poisson manifold (or Poisson vector space) $\left( \mathscr {P},\{\boldsymbol{\cdot },\,\boldsymbol{\cdot }\}\right)$ is a manifold $\mathscr {P}$ endowed with an operator acting on smooth functions (e.g., see (Holm et al. 2009; Salmon 1998))

1.5 $$\begin{aligned} \{\boldsymbol{\cdot }\,,\,\boldsymbol{\cdot }\} = C^\infty \left( \mathscr {P}\right) \times C^\infty \left( \mathscr {P}\right) \,\rightarrow \, C^\infty \left( \mathscr {P}\right) \,, \end{aligned}$$

called the Poisson bracket, satisfying the axioms

1.6 $$\begin{aligned} \begin{array}{rcl} & & \displaystyle \{\mathcal {F}+\mathcal {G}\,,\mathcal {K}\} = \displaystyle \{\mathcal {F}\,,\mathcal {K}\} +\{\mathcal {G}\,,\mathcal {K}\} \quad \text{(bilinear) } \,,\\ & & \displaystyle \{\mathcal {F}\,,\mathcal {K}\} = \displaystyle -\{\mathcal {K}\,,\mathcal {F}\} \quad \text{(antisymmetric) } \,,\\ & & \displaystyle \{\mathcal {F}\,,\{\mathcal {G}\,,\mathcal {K}\}\} +\{\mathcal {G}\,,\{\mathcal {K}\,,\mathcal {F}\}\} +\{\mathcal {K}\,,\{\mathcal {F}\,,\mathcal {G}\}\} = 0 \quad \text{(Jacobi } \text{ identity) } \,,\\ & & \displaystyle \{\mathcal {F}\,\mathcal {G}\,,\mathcal {K}\} = \{\mathcal {F}\,,\mathcal {K}\}\mathcal {G} +\mathcal {F}\{\mathcal {G}\,,\mathcal {K}\} \quad \text{(Leibniz } \text{ rule) }\,, \end{array} \end{aligned}$$

for any three functionals $\mathcal {F}$, $\mathcal {G}$ and $\mathcal {K}$. The first three properties in (1.6) identify a Lie algebra structure. The space $C^{\infty }\left( \mathscr {P}\right)$ is a Lie algebra with Lie bracket $[\boldsymbol{\cdot },\,\boldsymbol{\cdot }]=\{\boldsymbol{\cdot },\,\boldsymbol{\cdot }\}$. The Leibniz rule in (1.8) confers on this Lie algebra an extra structural property, so that the Lie algebra $C^{\infty }\left( \mathscr {P}\right)$ becomes a Poisson algebra.

Many models in fluid mechanics have a Hamiltonian structure which can be written in canonical or non-canonical Poisson bracket forms. Non-canonical Hamiltonian structures take the form

1.7 $$\begin{aligned} \boldsymbol{Z}_t = \textsf{J}\,\frac{\delta \mathcal {H}}{\delta \boldsymbol{Z}}\,, \end{aligned}$$

as evolution equations for the field variables $\boldsymbol{Z}$, where $\mathcal {H}$ is the Hamiltonian functional, and $\textsf{J}$ the Poisson tensor (e.g., see (Dellar 2003) and references therein). Equation (1.7) is equivalent to $\partial \mathcal {F}/\partial t=\{\mathcal {F},\mathcal {H}\}$ for any functional $\mathcal {F}$, where the Poisson bracket of any two functionals $\mathcal {A}$ and $\mathcal {B}$ in terms of the Poisson tensor $\textsf{J}$ is given by the volume integral

1.8 $$\begin{aligned} \{\mathcal {A}\,,\mathcal {B}\} = \int _{\textsf{D}} d\boldsymbol{x} \left( \frac{\delta \mathcal {A}}{\delta \boldsymbol{Z}}\right) ^T\textsf{J} \,\frac{\delta \mathcal {B}}{\delta \boldsymbol{Z}} \,. \end{aligned}$$

This bracket is bilinear in $\mathcal {A}$ and $\mathcal {B}$, and is antisymmetric when the Poisson tensor $\textsf{J}$ is antisymmetric, or when $\textsf{J}$ is an anti-selfadjoint differential operator (Dellar 2003). The bracket (1.8) is a Poisson bracket if it also satisfies the Jacobi identity in (1.6) (Salmon 1988; Shepherd 1990; Dellar 2003). The three properties of bilinearity, antisymmetry, and the Jacobi identity create a generalised Hamiltonian structure in the sense that is independent of any particular choice of coordinates (Salmon 1988; Shepherd 1990; Dellar 2003).

The forced SWEs in Eulerian coordinates (1.2) can be expressed in Hamiltonian Poisson-bracket form

1.9 $$\begin{aligned} \frac{d\,\mathcal {F}}{dt} = \{\mathcal {F}\,,\mathcal {H}\} \,, \end{aligned}$$

where $\mathcal {F}\left[ \boldsymbol{u},h\right]$ is an arbitrary functional of the fluid velocity $\boldsymbol{u}\left( x,y,t\right)$ and the fluid depth $h\left( x,y,t\right)$. The Hamiltonian $\mathcal {H}$, in terms of the particle velocity components $u\left( x,y,t\right)$ and $v\left( x,y,t\right)$, is the total energy

1.10 $$\begin{aligned} \mathcal {H}\left[ u,v,h\right] = \iint \, d\boldsymbol{x}\left( \frac{1}{2}h\left( u^2+v^2\right) +gh\left( \frac{1}{2}h+h_b\right) \right) \,. \end{aligned}$$

However, the Hamiltonian is considered as a functional of the canonical velocity components and the wave height, i.e. $\mathcal {H}=\mathcal {H}\big [\boldsymbol{u}\left( \widetilde{\boldsymbol{u}}\right) ,h\big ]$. The Poisson tensor in (1.7) for the forced SWEs (1.2) takes the form

1.11 $$\begin{aligned} \begin{array}{rcl} \textsf{J} & =& \displaystyle -\begin{pmatrix} 0 & -\mathcal {V} & \partial /\partial x \\ \mathcal {V} & 0 & \partial /\partial y \\ \partial /\partial x & \partial /\partial y & 0 \end{pmatrix}\,, \end{array} \end{aligned}$$

and the canonical variables are $\boldsymbol{Z}=\left( \widetilde{u}(\boldsymbol{x},t),\widetilde{v}(\boldsymbol{x},t),h(\boldsymbol{x},t)\right) ^T$. Hence, the bracket (1.8) for the forced SWEs takes the form

1.12 $$\begin{aligned} \begin{array}{rcl} \displaystyle \{\mathcal {A}\,,\mathcal {B}\}= &  \displaystyle \iint _{\textsf{D}}\, d\boldsymbol{x} \left[ \mathcal {V}\,\frac{\delta \left( \mathcal {A},\mathcal {B}\right) }{\delta \left( \widetilde{u},\widetilde{v}\right) } -\frac{\delta \mathcal {A}}{\delta \widetilde{\boldsymbol{u}}} \boldsymbol{\cdot }\boldsymbol{\nabla }\frac{\delta \mathcal {B}}{\delta h} -\frac{\delta \mathcal {A}}{\delta h}\, \boldsymbol{\nabla }\boldsymbol{\cdot }\frac{\delta \mathcal {B}}{\delta \widetilde{\boldsymbol{u}}}\right] \,, \end{array} \end{aligned}$$

where $\textsf{D}$ is the domain of integration, and $\boldsymbol{\nabla }=\left( \partial /\partial x,\partial /\partial y\right)$. But using the divergence theorem, the last term on the right-hand side becomes

1.13 $$\begin{aligned} \begin{array}{rcl} \displaystyle -\iint _{\textsf{D}} d\boldsymbol{x}\,\,\frac{\delta \mathcal {A}}{\delta h}\, \boldsymbol{\nabla }\boldsymbol{\cdot }\frac{\delta \mathcal {B}}{\delta \widetilde{\boldsymbol{u}}}= &  \displaystyle \iint _{\textsf{D}} d\boldsymbol{x}\,\,\frac{\delta \mathcal {B}}{\delta \widetilde{\boldsymbol{u}}} \boldsymbol{\cdot }\boldsymbol{\nabla }\frac{\delta \mathcal {A}}{\delta h} -\int _{\partial \textsf{D}}\frac{\delta \mathcal {A}}{\delta h} \frac{\delta \mathcal {B}}{\delta \widetilde{\boldsymbol{u}}}\boldsymbol{\cdot }d\boldsymbol{s}\,, \end{array} \end{aligned}$$

where $\partial \textsf{D}$ is the oriented boundary of the domain of integration $\textsf{D}$. Now substitution of (1.13) into (1.12) gives

1.14 $$\begin{aligned} \{\mathcal {A}\,,\mathcal {B}\} = \iint _{\textsf{D}}\, d\boldsymbol{x} \left[ \mathcal {V}\,\frac{\delta \left( \mathcal {A},\mathcal {B}\right) }{\delta \left( \widetilde{u},\widetilde{v}\right) } -\frac{\delta \mathcal {A}}{\delta \widetilde{\boldsymbol{u}}} \boldsymbol{\cdot }\boldsymbol{\nabla }\frac{\delta \mathcal {B}}{\delta h} +\frac{\delta \mathcal {B}}{\delta \widetilde{\boldsymbol{u}}} \boldsymbol{\cdot }\boldsymbol{\nabla }\frac{\delta \mathcal {A}}{\delta h}\right] -\int _{\partial \textsf{D}}\frac{\delta \mathcal {A}}{\delta h} \frac{\delta \mathcal {B}}{\delta \widetilde{\boldsymbol{u}}}\boldsymbol{\cdot }d\boldsymbol{s}\,. \end{aligned}$$

The boundary integral in (1.14) breaks the antisymmetry property required for a bracket operator to be a Poisson bracket. Hence, to make the differential operator (1.14) a Poisson bracket, the boundary integral on the right-hand side must vanish (e.g., using appropriate boundary conditions), or be cancelled out — calculating the volume integral using the divergence theorem (see below). Neglecting the boundary integral in (1.14) gives the *non-canonical shallow water Poisson-bracket* reported in the literature (e.g., see (Salmon 2004; Stewart and Dellar 2016)):

1.15 $$\begin{aligned} \{\mathcal {A}\,,\mathcal {B}\} = \iint _{\textsf{D}}\, d\boldsymbol{x} \left[ \mathcal {V}\left( \frac{\delta \mathcal {A}}{\delta \widetilde{u}} \frac{\delta \mathcal {B}}{\delta \widetilde{v}} -\frac{\delta \mathcal {A}}{\delta \widetilde{v}} \frac{\delta \mathcal {B}}{\delta \widetilde{u}}\right) -\frac{\delta \mathcal {A}}{\delta \widetilde{\boldsymbol{u}}} \boldsymbol{\cdot }\boldsymbol{\nabla }\frac{\delta \mathcal {B}}{\delta h} +\frac{\delta \mathcal {B}}{\delta \widetilde{\boldsymbol{u}}} \boldsymbol{\cdot }\boldsymbol{\nabla }\frac{\delta \mathcal {A}}{\delta h}\right] \,, \end{aligned}$$

which is antisymmetric. The functional derivatives are defined with respect to the canonical variables $\widetilde{\boldsymbol{u}}\left( x,y,t\right) =\left( \widetilde{u},\widetilde{v}\right)$ and $h\left( x,y,t\right)$.

The evolution equations (1.2) may be derived by setting $\mathcal {F}=\widetilde{u}\left( \boldsymbol{x}_0,t\right)$, $\mathcal {F}=\widetilde{v}\left( \boldsymbol{x}_0,t\right)$, and $\mathcal {F}=h\left( \boldsymbol{x}_0,t\right)$ for a fixed location $\boldsymbol{x}_0=\left( x_0,y_0\right)$ in (1.9). We note that for $\mathcal {F}=\widetilde{u}\left( \boldsymbol{x}_0,t\right)$ and $\mathcal {F}=\widetilde{v}\left( \boldsymbol{x}_0,t\right)$ the boundary integral in (1.14) is automatically vanished. For $\mathcal {F}=h_0=h\left( \boldsymbol{x}_0,t\right)$ the boundary integral in (1.14) is cancelled out using the divergence theorem:

1.16 $$\begin{aligned} \begin{array}{rcl} \displaystyle \frac{dh_0}{dt} = \{h_0\,,\,\mathcal {H}\} & =& \displaystyle \iint _{\textsf{D}}\, d\boldsymbol{x}\,\,\frac{\delta \mathcal {H}}{\delta \widetilde{\boldsymbol{u}}}\boldsymbol{\cdot }\boldsymbol{\nabla }\,\frac{\delta h\left( \boldsymbol{x}_0,t\right) }{\delta h\left( \boldsymbol{x},t\right) } -\int _{\partial \textsf{D}}\,\frac{\delta h\left( \boldsymbol{x}_0,t\right) }{\delta h\left( \boldsymbol{x},t\right) }\, \frac{\delta \mathcal {H}}{\delta \widetilde{\boldsymbol{u}}}\boldsymbol{\cdot }d\boldsymbol{s} \\ & =& \displaystyle \iint _{\textsf{D}} \,d\boldsymbol{x}\,\,h\boldsymbol{u}\boldsymbol{\cdot }\boldsymbol{\nabla }\delta \left( \boldsymbol{x}-\boldsymbol{x}_0\right) -\int _{\partial \textsf{D}}\,\delta \left( \boldsymbol{x}-\boldsymbol{x}_0\right) h\boldsymbol{u}\boldsymbol{\cdot }d\boldsymbol{s} \\ & =& \displaystyle \iint _{\textsf{D}} \,d\boldsymbol{x}\,\big [\boldsymbol{\nabla }\boldsymbol{\cdot }\left( \delta \left( \boldsymbol{x}-\boldsymbol{x}_0\right) h\boldsymbol{u}\right) -\delta \left( \boldsymbol{x}-\boldsymbol{x}_0\right) \boldsymbol{\nabla }\boldsymbol{\cdot }\left( h\boldsymbol{u}\right) \big ] \\ & & \displaystyle -\int _{\partial \textsf{D}}\,\delta \left( \boldsymbol{x}-\boldsymbol{x}_0\right) h\boldsymbol{u}\boldsymbol{\cdot }d\boldsymbol{s} \\ & =& \displaystyle \int _{\partial \textsf{D}}\,\delta \left( \boldsymbol{x}-\boldsymbol{x}_0\right) h\boldsymbol{u}\boldsymbol{\cdot }d\boldsymbol{s} -\iint _{\textsf{D}}\,d\boldsymbol{x}\,\,\delta \left( \boldsymbol{x}-\boldsymbol{x}_0\right) \boldsymbol{\nabla }\boldsymbol{\cdot }\left( h\boldsymbol{u}\right) \\ & & \displaystyle -\int _{\partial \textsf{D}}\,\delta \left( \boldsymbol{x}-\boldsymbol{x}_0\right) h\boldsymbol{u}\boldsymbol{\cdot }d\boldsymbol{s}\\ & =& \displaystyle -\boldsymbol{\nabla }\boldsymbol{\cdot }\left( h\boldsymbol{u}\right) \bigg |_{\boldsymbol{x}=\boldsymbol{x}_0}\,, \end{array} \end{aligned}$$

where $\delta \left( \boldsymbol{x}-\boldsymbol{x}_0\right)$ is the Dirac delta function in two dimensions. This recovers the mass conservation equation in (1.2).

The total energy is conserved $d\mathcal {H}/dt=\{\mathcal {H},\mathcal {H}\}=0$ because the shallow water Poisson-bracket (1.15) is antisymmetric in its two arguments. We also note that in the presence of boundaries with combined no-flow and inflow-outflow boundary conditions, if we use the bracket (1.14) to construct an energy preserving numerical algorithm, we should ensure that the boundary terms in (A.1) (see Appendix A) vanish using appropriate boundary conditions, e.g., using (a) periodic boundary conditions for the field variables $\left( u,v,h\right)$, and setting $h_b\left( 0,y\right) =h_b\left( L_1,y\right)$ and $h_b\left( x,0\right) =h_b\left( x,L_2\right)$, or (b) using the no-flow boundary conditions in (3.3). In this paper we use the (antisymmetric) shallow water Poisson-bracket (1.15) to design a structure-preserving numerical algorithm.

The shallow-water Poisson-bracket (1.15) also conserves Casimir functionals of the form

1.17 $$\begin{aligned} \mathcal {C} = \iint \, d\boldsymbol{x}\,\big [h\,c\left( \mathcal {V}\right) \big ] \,, \end{aligned}$$

where $c\left( \mathcal {V}\right)$ is an arbitrary function of the potential vorticity. This means $\{\mathcal {F},\mathcal {C}\}=0$ for any functional $\mathcal {F}$, which expresses the material conservation of potential vorticity in an Eulerian setting (Stewart and Dellar 2016). Important cases of Casimir functionals include the total mass $\mathcal {M}$, the circulation ω, and the potential enstrophy $\mathcal {P}$,

1.18 $$\begin{aligned} \mathcal {M} = \iint \, d\boldsymbol{x}\,\big [h\big ]\,,\quad \omega = \iint \, d\boldsymbol{x}\,\big [h\,\mathcal {V}\big ]\quad \text{ and }\quad \mathcal {P} = \iint \, d\boldsymbol{x}\,\big [\frac{1}{2}h\,\mathcal {V}^2\big ] \,. \end{aligned}$$

It is shown in Salmon (2004) that the Arakawa–Lamb scheme is equivalent to a discrete Hamiltonian system that preserves a discrete version of the conservation laws for the potential enstrophy and total mass, when the discretisation is properly constructed.

The paper is organised as follows. A review of the fourth-order C-bracket discretisation based on the antisymmetric shallow water bracket (1.15) is presented in §2. The combined sloshing and inflow-outflow boundary conditions, in the context of the fourth-order C-bracket discretisation based on the shallow water bracket (1.15), for shallow-water sloshing in a rigid rectangular tank with both symmetric and non-symmetric porous side walls are discussed in §3. Long-time structure-preserving sloshing simulations, with porous boundaries and periodic/non-periodic inflow-outflow boundary conditions, and with biharmonic dissipation are presented in §4. The paper ends with concluding remarks in §5.

## Fourth-order Poisson-bracket spatial discretisation

In this section we present an overview of the total mass-, energy-, and potential-enstrophy-conserving finite-difference scheme introduced by Arakawa & Lamb (1981) for the shallow-water equations. This scheme was generalised by Salmon (2004) to fourth-order Takano–Wurtele type schemes on the Arakawa C-grid using a Poisson-bracket approach. Stewart & Dellar (2016) extended the second-order Arakawa–Lamb scheme, in the Poisson-bracket form introduced by Salmon (2004), and applied to the single and multi-layer SWEs with complete Coriolis force and periodic boundary conditions.

To the best our knowledge, *the fourth-order C-bracket discretisation* based on the antisymmetric shallow water Poisson-bracket (1.15) is only introduced in Salmon (2004) and used for the shallow-water equations with *periodic* boundary conditions. The *fourth-order* scheme has not been applied to shallow-water *sloshing* problems, e.g., the forced SWEs with the *combined sloshing and non-periodic inflow-outflow* boundary conditions (as in the current paper). Alemi Ardakani and Bridges (2024) adapted the *second-order* C-bracket discretisation of Salmon (2004) to the problem of shallow-water sloshing over a variable bottom surface in an oscillating rigid rectangular tank with the sloshing boundary conditions only.

In the current paper, to improve the simulation of nonlinear aspects of the shallow-water sloshing over an *irregular bottom surface*, and hence to provide a robust, stable, and accurate scheme for long-time computational modelling of the sloshing problem in the FlexSlosh WEC with a bottom surface and inflow-outflow mass fluxes, the fourth-order Poisson-bracket discretisation based on the antisymmetric shallow-water bracket (1.15) is extracted from the generalised algorithms of Salmon (2004), and accompanied by the *coupled sloshing and inflow-outflow boundary conditions* on the Arakawa C grid. We study both the periodic and non-periodic inflow-outflow boundary conditions. See §3 for more details on the boundary conditions. The presented numerical algorithm is based on the discrete version of the antisymmetric shallow water Poisson-bracket (1.15), i.e. without the boundary integral term in (1.14). We leave the discretisation and algorithm design based on the bracket in (1.14) for further study (see also §5). However, we propose an alternative approach to find the required inflow-outflow boundary conditions which negate the boundary integrals that emerge from integration by parts in enforcement of the potential-enstrophy-conserving Casimir property of the Poisson-bracket (see §3).

We note that the presented numerical scheme in this section does not preserve the discrete version of the Jacobi identity in (1.6), but it preserves the discrete version of the conservation laws for the potential enstrophy and total mass.

**Fig. 2:**
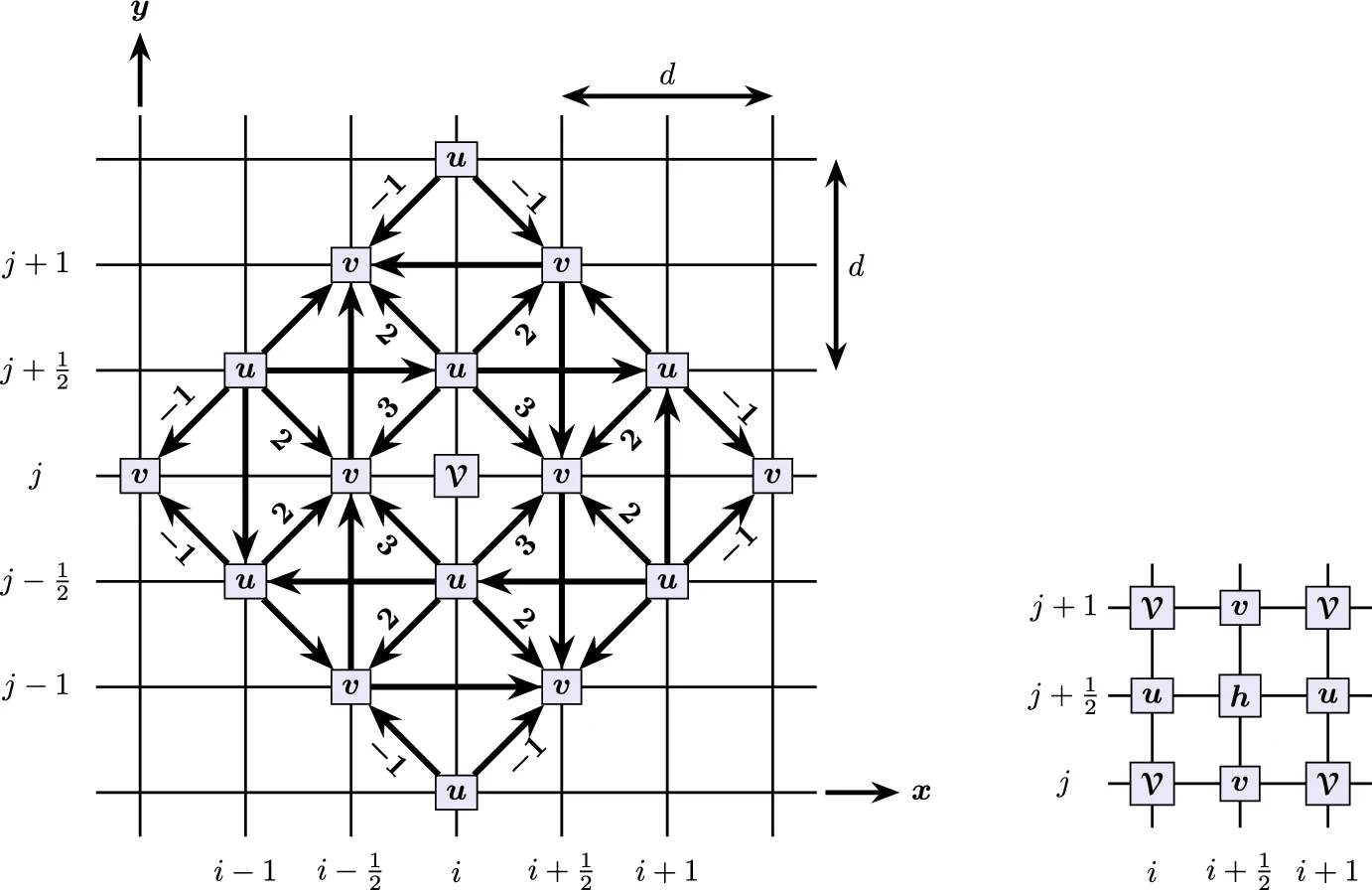
On the left, the stencil for the C-bracket discretisation with fourth-order accuracy in the potential vorticity dependent component of the Poisson-bracket (1.15). Each of the 36 arrows represents a term in the discrete Poisson-bracket (2.6). The four arrows labelled with “3” have weight 3/24; the eight arrows labelled with “2” have weight 2/24; the eight arrows labelled with “-1” have weight -1/24; the other sixteen arrows, which are not labelled, have weight 1/24. On the right, the layout of the variables within a single computational cell of side length *d*.

Exactly how the derivatives in the *x*- and *y*-directions are discretised will be important for enforcing discrete conservation of mass and potential enstrophy. In the Arakawa C grid, the variables are *u*(*x*, *y*, *t*), *v*(*x*, *y*, *t*), *h*(*x*, *y*, *t*), and the potential vorticity $\mathcal {V}\left( x,y,t\right)$ on a staggered grid. The C grid may be thought of as a mesh of square cells of side length *d*, or rectangular cells with different side lengths in the *x* and *y* directions. The wave height *h* is defined at cell centres, the fluid velocity components *u* and *v* are defined at cell faces, and the potential vorticity $\mathcal {V}$ is defined at cell vertices. See Fig. 2 for the staggered layout of the variables on the Arakawa C grid.

For *long-time* nonlinear shallow-water sloshing simulations, simultaneous conservation of energy and potential enstrophy is particularly important because it prevents the spurious cascade of energy into high wavenumbers (e.g., see Salmon (2004); Stewart and Dellar (2016)). The Hamiltonian Poisson-bracket framework can accommodate these desirable numerical requirements.

As discussed by Salmon (2004), the Arakawa–Lamb scheme is equivalent to a discrete Hamiltonian Poisson-bracket system with a discrete Hamiltonian of the form (see also Stewart and Dellar (2016))

2.1 $$\begin{aligned} \begin{array}{rcl} H & =& \displaystyle \sum _{i,j}\big [\,\frac{1}{2}h_{i+1/2,j+1/2} \left( \overline{u^2}^{\,x}_{i+1/2,j+1/2}+\overline{v^2}^{\,y}_{i+1/2,j+1/2}\right) \\ & & \displaystyle +gh_{i+1/2,j+1/2}\left( \frac{1}{2}h+h_b\right) _{i+1/2,j+1/2}\,\big ] \,, \end{array} \end{aligned}$$

that approximates (1.10), where

2.2 $$\begin{aligned} \overline{u^2}^{\,x}_{i+1/2,j+1/2} = \frac{1}{2}\left( u_{i,j+1/2}^2+u_{i+1,j+1/2}^2\right) \,,\,\, \overline{v^2}^{\,y}_{i+1/2,j+1/2} = \frac{1}{2}\left( v_{i+1/2,j}^2+v_{i+1/2,j+1}^2\right) \,. \end{aligned}$$

The sum ranges over all cells in the computational grid. See Fig. 1 for the computational grid and its indices. The evolution of any function $F\left( u_{i,j+1/2},v_{i+1/2,j},\right. \left. h_{i+1/2,j+1/2}\right)$ is given by the discrete version of (1.9)

2.3 $$\begin{aligned} \frac{dF}{dt} = \{F\,,H\}\,. \end{aligned}$$

The exact Poisson bracket (1.15) is split into a potential vorticity dependent part and a remainder (Salmon 2004; Stewart and Dellar 2016)

2.4 $$\begin{aligned} \{A\,,B\} = \{A\,,B\}_{\mathcal {V}}+\{A\,,B\}_{R} \,, \end{aligned}$$

where *A* and *B* are algebraic functions of $u_{i,j+1/2}$, $v_{i+1/2,j}$, and $h_{i+1/2,j+1/2}$ for all *i* and *j*. The discrete approximation to the remainder part of the bracket in (2.4) is

2.5 $$\begin{aligned} \begin{array}{rcl} \{A\,,B\}_R & =& \displaystyle \frac{1}{d}\sum _{i,j} \bigg [\frac{\partial B}{\partial \widetilde{u}_{i,j+1/2}} \left( \frac{\partial A}{\partial h_{i+1/2,j+1/2}} -\frac{\partial A}{\partial h_{i-1/2,j+1/2}}\right) \\ & & \displaystyle +\frac{\partial B}{\partial \widetilde{v}_{i+1/2,j}} \left( \frac{\partial A}{\partial h_{i+1/2,j+1/2}} -\frac{\partial A}{\partial h_{i+1/2,j-1/2}}\right) -\left( A\longleftrightarrow B\right) \bigg ] \,. \end{array} \end{aligned}$$

where $\left( A\longleftrightarrow B\right)$ stands for the preceding terms in (2.5) with interchange of *A* and *B* to enforce the antisymmetry. In (2.5), *d* is the grid spacing (see Fig. 2). The discrete approximation to the potential vorticity dependent part of the bracket is

2.6 $$\begin{aligned} \begin{array}{rcl} \{A\,,B\}_{\mathcal {V}} & =& \displaystyle \sum _{i,j}\mathcal {V}_{i,j}\sum _{\textbf{n},\textbf{m}} \bigg (\alpha _{\textbf{n},\textbf{m}}\,\frac{\partial \left( A,B\right) }{\partial \left( \widetilde{u}_{\left( i,j+1/2\right) +\textbf{n}}, \widetilde{v}_{\left( i+1/2,j\right) +\textbf{m}}\right) } \\ & & \displaystyle \hspace{-1.5cm}+\beta _{\textbf{n},\textbf{m}}\, \frac{\partial \left( A,B\right) }{\partial \left( \widetilde{u}_{\left( i,j+1/2\right) +\textbf{n}}, \widetilde{u}_{\left( i,j+1/2\right) +\textbf{m}}\right) } +\gamma _{\textbf{n},\textbf{m}}\,\frac{\partial \left( A,B\right) }{\partial \left( \widetilde{v}_{\left( i+1/2,j\right) +\textbf{n}}, \widetilde{v}_{\left( i+1/2,j\right) +\textbf{m}}\right) }\bigg )\,, \end{array} \end{aligned}$$

where

2.7 $$\begin{aligned} \frac{\partial \left( A,B\right) }{\partial \left( \widetilde{u},\widetilde{v}\right) } = \frac{\partial A}{\partial \widetilde{u}}\frac{\partial B}{\partial \widetilde{v}} -\frac{\partial B}{\partial \widetilde{u}}\frac{\partial A}{\partial \widetilde{v}} \,, \end{aligned}$$

is the Jacobian of *A* and *B* with respect to $\widetilde{u}$ and $\widetilde{v}$. The indices $\textbf{n}=\left( n_x,n_y\right)$ and $\textbf{m}=\left( m_x,m_y\right)$ are pairs of integers. The sums over $\textbf{n}$ and $\textbf{m}$ represent sums over grid points near $\left( i,j\right)$. That is, the coefficients $\alpha _{\textbf{n},\textbf{m}}$, $\beta _{\textbf{n},\textbf{m}}$, $\gamma _{\textbf{n},\textbf{m}}$ vanish if $\textbf{n}=\left( n_x,n_y\right)$ or $\textbf{m}=\left( m_x,m_y\right)$ lies outside a relatively small prescribed set $\{\left( n_x,n_y\right) \}$ or $\{\left( m_x,m_y\right) \}$ in which $|n_x|$, $|n_y|$, $|m_x|$, $|m_y|\le M$, where *M* is a small integer called the stencil size of the corresponding discrete algorithm. The first term proportional to $\alpha _{\textbf{n},\textbf{m}}$ approximates the term proportional to $\mathcal {V}$ in the continuous Poisson bracket (1.15). The additional terms proportional to $\beta _{\textbf{n},\textbf{m}}$ and $\gamma _{\textbf{n},\textbf{m}}$ introduce the unphysical Coriolis terms in the Arakawa–Lamb scheme that are needed to conserve the potential enstrophy $\mathcal {P}$ (Salmon 2004; Stewart and Dellar 2016).

The discrete potential enstrophy is defined by Salmon (2004); Stewart and Dellar (2016)

2.8 $$\begin{aligned} P = \sum _{i,j}\overline{h}_{i,j}^{\,xy}\,\mathcal {V}_{i,j}^2 \,, \end{aligned}$$

where

2.9a $$\begin{aligned} \overline{h}_{i,j}^{\,xy}&= \displaystyle \frac{1}{4}\left( h_{i+1/2,j+1/2}+h_{i-1/2,j+1/2} +h_{i+1/2,j-1/2}+h_{i-1/2,j-1/2}\right) \,,\end{aligned}$$

2.9b $$\begin{aligned} \mathcal {V}_{i,j}&= \displaystyle \frac{1}{\overline{h}_{i,j}^{\,xy}}\frac{1}{d} \left( v_{i+1/2,j}-v_{i-1/2,j}-u_{i,j+1/2}+u_{i,j-1/2}\right) \,. \end{aligned}$$

The discrete total mass is defined by

2.10 $$\begin{aligned} M = \sum _{i,j} h_{i+1/2,j+1/2} \,. \end{aligned}$$

The antisymmetry of the discrete Poisson-bracket in (2.3) and (2.4) implies conservation of the discrete energy by the discrete analog of $d\mathcal {H}/dt=\{\mathcal {H},\mathcal {H}\}=0$, i.e.

2.11 $$\begin{aligned} \frac{dH}{dt} = \{H\,,H\} =0 \,. \end{aligned}$$

Conservation of *P* and *M* is satisfied if they are Casimirs of the discrete Poisson bracket (2.4), i.e.

2.12 $$\begin{aligned} \{F\,,M\} = \{F\,,P\} = 0 \quad \text{ for } \text{ any } F\left( u_{i,j+1/2},v_{i+1/2,j},h_{i+1/2,j+1/2}\right) \,. \end{aligned}$$

By the chain rule, this is equivalent to requiring

2.13a $$\begin{aligned} \displaystyle \{\widetilde{u}_{i,j+1/2}\,,M\}&= \displaystyle \{\widetilde{v}_{i+1/2,j}\,,M\} = \{h_{i+1/2,j+1/2}\,,M\} = 0 \quad \,\forall \,\, i,j \,,\end{aligned}$$

2.13b $$\begin{aligned} \displaystyle \{\widetilde{u}_{i,j+1/2}\,,P\}&= \displaystyle \{\widetilde{v}_{i+1/2,j}\,,P\} = \{h_{i+1/2,j+1/2}\,,P\} = 0 \quad \,\forall \,\, i,j \,. \end{aligned}$$

The coefficients $\alpha _{\textbf{n},\textbf{m}}$, $\beta _{\textbf{n},\textbf{m}}$ and $\gamma _{\textbf{n},\textbf{m}}$ in (2.6) must be chosen such that the discrete potential enstrophy *P* in (2.8) and the discrete total mass *M* in (2.10) are Casimirs, i.e. $\alpha _{\textbf{n},\textbf{m}}$, $\beta _{\textbf{n},\textbf{m}}$ and $\gamma _{\textbf{n},\textbf{m}}$ must satisfy (2.13).

The discrete total mass *M* satisfies the conditions in (2.13a) for any $\alpha _{\textbf{n},\textbf{m}}$, $\beta _{\textbf{n},\textbf{m}}$ and $\gamma _{\textbf{n},\textbf{m}}$. The condition $\{h_{i+1/2,j+1/2},P\}=0$ in (2.13b) is also satisfied (see the equation (4.15) in Salmon (2004)). To obtain a potential-enstrophy-conserving scheme we must choose the coefficients $\alpha _{\textbf{n},\textbf{m}}$, $\beta _{\textbf{n},\textbf{m}}$ and $\gamma _{\textbf{n},\textbf{m}}$ such that the conditions $\{\widetilde{u}_{i,j+1/2},P\}=0$ and $\displaystyle \{\widetilde{v}_{i+1/2,j},P\}=0$ in (2.13b) are satisfied for arbitrary $\mathcal {V}_{i,j}$ values. For a *fourth-order C-bracket Takano–Wurtele type scheme* these coefficients can be obtained from the equations (4.19), (4.20), (4.21), (6.11), and (6.12) of Salmon (2004). A diagrammatic representation of the $\alpha _{\textbf{n},\textbf{m}}$, $\beta _{\textbf{n},\textbf{m}}$ and $\gamma _{\textbf{n},\textbf{m}}$ terms in (2.6) for the fourth-order C-bracket discretisation, i.e. fourth-order accurate in the potential vorticity dependent part of the Poisson bracket, is shown in Fig. 2. It is a combination of the stencil in Fig. 2 and the $\gamma _1$-stencil in Fig. 5 of Salmon (2004). Since the *u* and *v* grid points never coincide on the Arakawa C-grid, the diagonal arrows in Fig. 2 correspond to $\alpha _{\textbf{n},\textbf{m}}$ terms; the horizontal and vertical arrows connecting the *u* grid points correspond to $\beta _{\textbf{n},\textbf{m}}$ terms; and the horizontal and vertical arrows connecting the *v* grid points correspond to $\gamma _{\textbf{n},\textbf{m}}$ terms. Each arrow’s weight is defined in Fig. 2.

The fourth-order Poisson-bracket semi-discretisations of the evolution equations (1.2), using (2.3) with the discrete Hamiltonian (2.1) and the discrete Poisson brackets (2.5) and (2.6), with the $\alpha _{\textbf{n},\textbf{m}}$, $\beta _{\textbf{n},\textbf{m}}$, $\gamma _{\textbf{n},\textbf{m}}$ weights defined in Fig. 2, take the form:

2.14 $$\begin{aligned} \begin{array}{rcl} \displaystyle \frac{d\,}{dt}h_{i+1/2,j+1/2}= &  \displaystyle \frac{1}{d} \left( U_{i,j+1/2}-U_{i+1,j+1/2}+V_{i+1/2,j}-V_{i+1/2,j+1}\right) \,, \end{array} \end{aligned}$$

and

2.15 $$\begin{aligned} \begin{array}{rcl} & & \displaystyle \frac{d\,}{dt}\left( u_{i,j+1/2}+\dot{q}_1\right) \\ & =& \displaystyle -\frac{1}{d}\left( \Phi _{i+1/2,j+1/2}-\Phi _{i-1/2,j+1/2}\right) \\ & & \displaystyle +\frac{1}{24}\bigg [\big (2\mathcal {V}_{i,j}+\mathcal {V}_{i+1,j} +2\mathcal {V}_{i+1,j+1}+3\mathcal {V}_{i,j+1} -\mathcal {V}_{i,j+2}-\mathcal {V}_{i-1,j+1}\big )V_{i+1/2,j+1} \\ & & \displaystyle +\big (\mathcal {V}_{i-1,j}+2\mathcal {V}_{i,j}+3\mathcal {V}_{i,j+1} +2\mathcal {V}_{i-1,j+1}-\mathcal {V}_{i+1,j+1}-\mathcal {V}_{i,j+2}\big )V_{i-1/2,j+1} \\ & & \displaystyle +\big (2\mathcal {V}_{i-1,j}+3\mathcal {V}_{i,j}+2\mathcal {V}_{i,j+1} +\mathcal {V}_{i-1,j+1}-\mathcal {V}_{i,j-1}-\mathcal {V}_{i+1,j}\big )V_{i-1/2,j} \\ & & \displaystyle +\big (3\mathcal {V}_{i,j}+2\mathcal {V}_{i+1,j}+\mathcal {V}_{i+1,j+1} +2\mathcal {V}_{i,j+1}-\mathcal {V}_{i,j-1}-\mathcal {V}_{i-1,j}\big )V_{i+1/2,j} \\ & & \displaystyle +\big (\mathcal {V}_{i,j}+\mathcal {V}_{i+1,j} -\mathcal {V}_{i+1,j+1}-\mathcal {V}_{i,j+1}\big )U_{i+1,j+1/2} \\ & & \displaystyle +\big (-\mathcal {V}_{i-1,j}-\mathcal {V}_{i,j}+\mathcal {V}_{i,j+1} +\mathcal {V}_{i-1,j+1}\big )U_{i-1,j+1/2} \\ & & \displaystyle +\big (\mathcal {V}_{i+1,j}-\mathcal {V}_{i-1,j}\big )U_{i,j-1/2} +\big (-\mathcal {V}_{i+1,j+1}+\mathcal {V}_{i-1,j+1}\big )U_{i,j+3/2}\bigg ] \,, \end{array} \end{aligned}$$

and

2.16 $$\begin{aligned} \begin{array}{rcl} & & \displaystyle \frac{d\,}{dt}\left( v_{i+1/2,j}+\dot{q}_2\right) \\ & =& \displaystyle -\frac{1}{d}\left( \Phi _{i+1/2,j+1/2}-\Phi _{i+1/2,j-1/2}\right) \\ & & \displaystyle -\frac{1}{24}\bigg [\big (2\mathcal {V}_{i,j-1}+\mathcal {V}_{i+1,j-1} +2\mathcal {V}_{i+1,j}+3\mathcal {V}_{i,j} -\mathcal {V}_{i,j+1}-\mathcal {V}_{i-1,j}\big )U_{i,j-1/2} \\ & & \displaystyle +\big (\mathcal {V}_{i,j-1}+2\mathcal {V}_{i+1,j-1} +3\mathcal {V}_{i+1,j}+2\mathcal {V}_{i,j} -\mathcal {V}_{i+2,j}-\mathcal {V}_{i+1,j+1}\big )U_{i+1,j-1/2} \\ & & \displaystyle +\big (2\mathcal {V}_{i,j}+3\mathcal {V}_{i+1,j}+2\mathcal {V}_{i+1,j+1} +\mathcal {V}_{i,j+1}-\mathcal {V}_{i+1,j-1}-\mathcal {V}_{i+2,j}\big )U_{i+1,j+1/2} \\ & & \displaystyle +\big (3\mathcal {V}_{i,j}+2\mathcal {V}_{i+1,j}+\mathcal {V}_{i+1,j+1} +2\mathcal {V}_{i,j+1}-\mathcal {V}_{i,j-1}-\mathcal {V}_{i-1,j}\big )U_{i,j+1/2} \\ & & \displaystyle +\big (-\mathcal {V}_{i,j-1}+\mathcal {V}_{i+1,j-1}+\mathcal {V}_{i+1,j} -\mathcal {V}_{i,j}\big )V_{i+1/2,j-1} \\ & & \displaystyle +\big (\mathcal {V}_{i,j}-\mathcal {V}_{i+1,j}-\mathcal {V}_{i+1,j+1} +\mathcal {V}_{i,j+1}\big )V_{i+1/2,j+1} \\ & & \displaystyle +\big (-\mathcal {V}_{i,j-1}+\mathcal {V}_{i,j+1}\big )V_{i-1/2,j} +\big (\mathcal {V}_{i+1,j-1}-\mathcal {V}_{i+1,j+1}\big )V_{i+3/2,j}\bigg ] \,, \end{array} \end{aligned}$$

where

2.17 $$\begin{aligned} \begin{array}{rcl} U_{i,j+1/2} & =& \displaystyle \frac{1}{2}\left( h_{i+1/2,j+1/2} +h_{i-1/2,j+1/2}\right) u_{i,j+1/2} \,,\\ V_{i+1/2,j} & =& \displaystyle \frac{1}{2}\left( h_{i+1/2,j+1/2} +h_{i+1/2,j-1/2}\right) v_{i+1/2,j} \,, \end{array} \end{aligned}$$

and

2.18 $$\begin{aligned} \Phi _{i+1/2,j+1/2} = \frac{1}{2}\left( \overline{u^2}^{\,x}_{i+1/2,j+1/2} +\overline{v^2}^{\,y}_{i+1/2,j+1/2}\right) +g\left( h+h_b\right) _{i+1/2,j+1/2} \,. \end{aligned}$$

For the numerical simulations in this paper, we integrate the semi-discretisations (2.14), (2.15) and (2.16) forward in time using the standard fourth-order Runge–Kutta scheme for *nonautonomous systems* with a constant timestep. Alternatively, one could use fractional-step splitting or Strang-splitting methods (e.g., Durran (1999)) to split the nonautonomous dissipative dynamics into a series of fractional steps. See §2.1 for the incorporation of viscosity into our numerical discretisation.

### Biharmonic dissipation

For the numerical simulations, we will compare the structure-preserving simulations with no dissipation against the simulations with a standard dissipation method. A common approach that is used to remove small scale noise from the potential enstrophy field and improve the stability of the numerical scheme over long-time integration is to apply a linear *fourth-order diffusion* or *biharmonic dissipation* to the velocity field in the shallow-water momentum equations (see e.g., (Sadourny and Basdevant 1985; Ringler and Randall 2002; Flyer et al. 2012; Brecht et al. 2021)). For the shallow-water equations (1.2) this yields

2.19 $$\begin{aligned} \widetilde{u}_t-\mathcal {V}hv+\Phi _x = -\nu \Delta ^2u\,,\quad \widetilde{v}_t+\mathcal {V}hu+\Phi _y = -\nu \Delta ^2v\,,\quad h_t+\left( hu\right) _x+\left( hv\right) _y = 0 \,, \end{aligned}$$

where ν is the diffusion coefficient and

2.20 $$\begin{aligned} \Delta ^2 u = \frac{\partial ^4u}{\partial x^4}+2\, \frac{\partial ^4u}{\partial x^2\,\partial y^2} +\frac{\partial ^4u}{\partial y^4}\,. \end{aligned}$$

The function $u\left( x,y,t\right)$ minimising the value of the functional

2.21 $$\begin{aligned} \mathcal {I}\left( u\right) = \iint _{\textsf{D}} d\boldsymbol{x}\, \left[ \left( u_{xx}\right) ^2+2\left( u_{xy}\right) ^2+\left( u_{yy}\right) ^2\right] \,, \end{aligned}$$

over a given domain $\textsf{D}$, subject to suitable conditions on the boundary $\partial \textsf{D}$, can be shown to satisfy the biharmonic equation $\Delta ^2u=0$ on $\textsf{D}$. We discretise the functional (2.21) using the second-order central finite-difference approximation, and extract the discrete version of the fourth-order diffusion term on the right-hand side of the *x*-momentum equations in (2.19) using the discrete variational derivative. This gives the following spatial discretisations for the dissipation term in the *x*-momentum equation in (2.19) on the C-grid:

2.22 $$\begin{aligned} \begin{array}{rcl} -\nu \left( \Delta ^2 u\right) _{i,j+1/2} & =& \displaystyle -\frac{\nu }{8d^4}\big ( u_{i-2,j+5/2}+6u_{i-2,j+1/2}+u_{i-2,j-3/2}-32u_{i-1,j+1/2} \\ & & \displaystyle +6u_{i,j+5/2}-32u_{i,j+3/2}+100u_{i,j+1/2}-32u_{i,j-1/2}+6u_{i,j-3/2} \\ & & \displaystyle -32u_{i+1,j+1/2}+u_{i+2,j+5/2}+6u_{i+2,j+1/2}+u_{i+2,j-3/2}\big ) \,. \end{array} \end{aligned}$$

Using a similar argument we obtain the discrete version of the fourth-order diffusion term on the right-hand side of the *y*-momentum equation in (2.19) on the C-grid:

2.23 $$\begin{aligned} \begin{array}{rcl} -\nu \left( \Delta ^2 v\right) _{i+1/2,j} & =& \displaystyle -\frac{\nu }{8d^4}\big ( v_{i-3/2,j+2}+6v_{i-3/2,j}\\ & & +v_{i-3/2,j-2}-32v_{i-1/2,j}+6v_{i+1/2,j+2} \\ & & \displaystyle -32v_{i+1/2,j+1}+100v_{i+1/2,j}-32v_{i+1/2,j-1}+6v_{i+1/2,j-2} \\ & & \displaystyle -32v_{i+3/2,j}+v_{i+5/2,j+2}+6v_{i+5/2,j}+v_{i+5/2,j-2}\big ) \,. \end{array} \end{aligned}$$

For the numerical simulations with biharmonic dissipation, the discretisations (2.22) and (2.23) can be added to the right-hand side of the Poisson-bracket semi-discretisations (2.15) and (2.16), respectively.

## The sloshing and inflow-outflow boundary conditions on the Arakawa C grid with symmetric and non-symmetric porous boundaries

For the problem of shallow-water sloshing in a rigid rectangular tank, a Poisson bracket argument is used in Alemi Ardakani and Bridges (2024) to impose a zero-potential-vorticity boundary condition along the solid boundaries, i.e. $\mathcal {V}\left( 0,y,t\right) =\mathcal {V}\left( L_1,y,t\right) =\mathcal {V}\left( x,0,t\right) =\mathcal {V}\left( x,L_2,t\right) =0$, in the context of the second-order Poisson-bracket discretisation on the Arakawa C grid, which respects the potential-enstrophy-conserving property of the shallow-water Poisson bracket (1.15).

In the current paper, for our problem of shallow-water sloshing in a rigid rectangular tank with *inflow-outflow mass fluxes*, we extend the Poisson bracket approach of Alemi Ardakani and Bridges (2024) to obtain *the coupled sloshing and inflow-outflow boundary conditions*, in the context of *the fourth-order C-bracket discretisation*, which respect the potential-enstrophy-conserving property of the shallow-water Poisson bracket (1.15), i.e. $\{\mathcal {F},\mathcal {P}\}=0$ for any functional $\mathcal {F}$.

We first propose a particular arrangement of the inlets/outlets, with periodic inflow-outflow boundary conditions, to vanish the *boundary integrals* that arise in enforcement of the potential-enstrophy-conserving property of the shallow-water Poisson bracket. We then generalise our argument for any arbitrary arrangement of the inlets/outlets with non-periodic inflow-outflow mass fluxes. In this case, we discuss that the condition on global conservation of the Casimir property in (3.1c) might be relaxed.

### Symmetric porous boundaries with periodic inflow-outflow mass fluxes

**Fig. 3:**
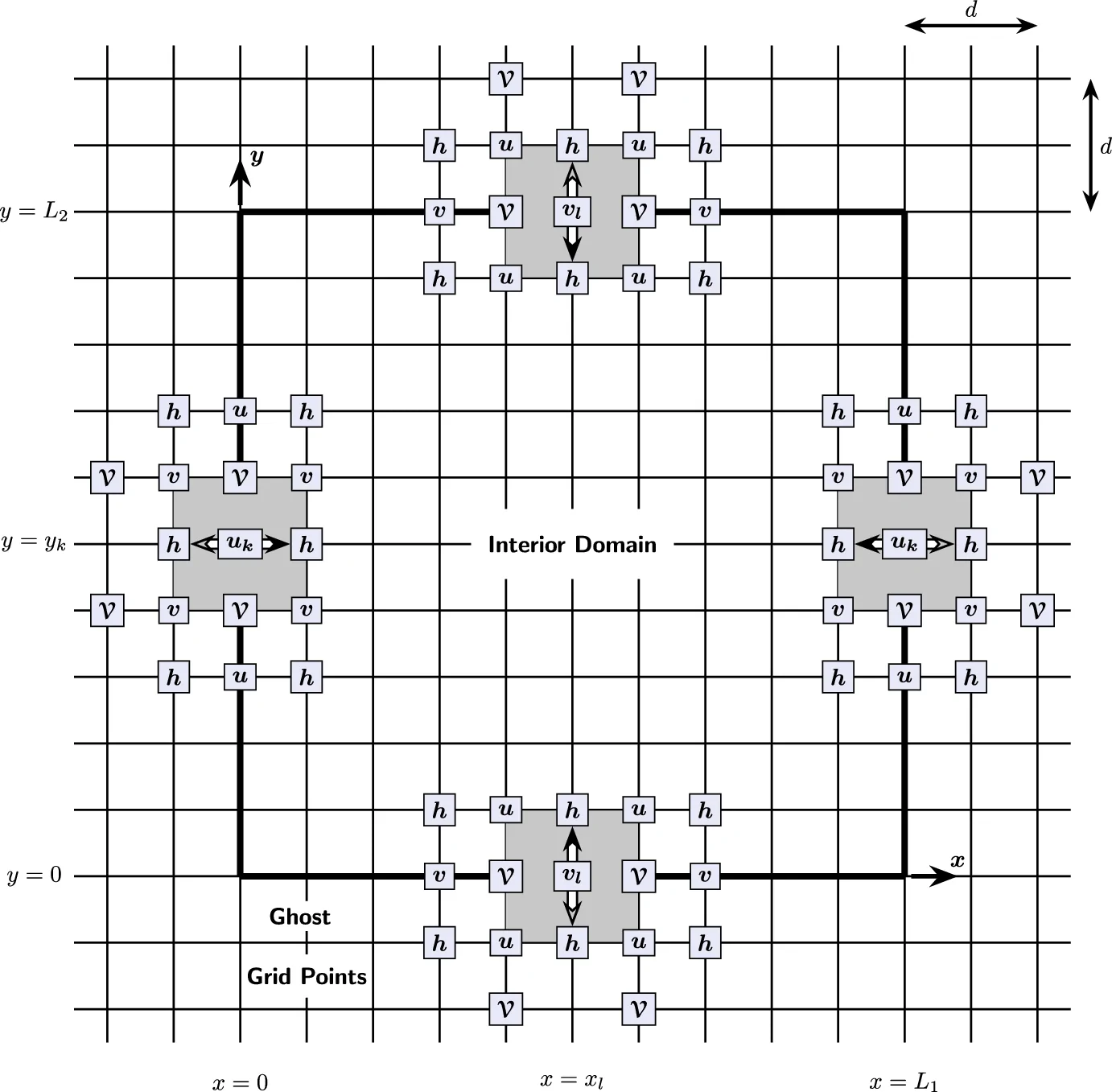
The computational grid with inlets/outlets depicted in gray cells along the *x*-direction and *y*-direction boundaries. The periodic boundary conditions (3.6), (3.8), (3.20) and (3.35) are used for the highlighted exterior/ghost *h* grid points. The symmetric boundary conditions (3.17) and (3.32) are used for the highlighted exterior/ghost *v* and *u* grid points, respectively. For the simulations in this paper, the boundary conditions (3.24) and (3.37) are used for the highlighted ghost potential vorticity $\mathcal {V}$ grid points in the *x*-direction and *y*-direction, respectively. The boundary lines (rigid side walls) are the coordinate lines $\{(x,y)\,:\,x=0,L_1\}$ in the *x*-direction, and $\{(x,y)\,:\,y=0,L_2\}$ in the *y*-direction. The sloshing boundary conditions are applied along the rigid side walls.

**Fig. 4:**
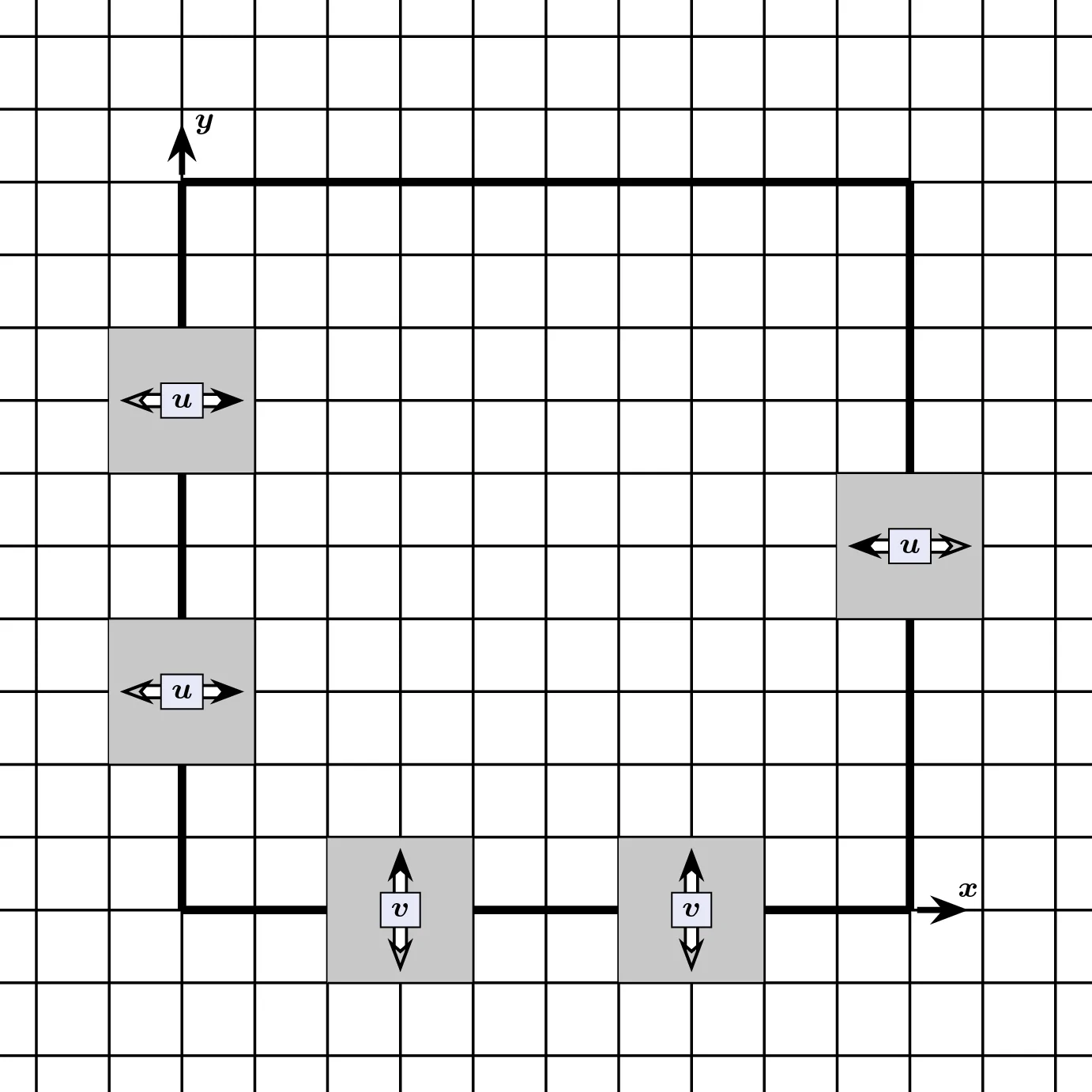
Non-symmetric arrangement of the inlets/outlets depicted in gray cells along the solid boundaries with non-periodic inflow-outflow mass fluxes.

The computational grid for the sloshing problem in a rigid rectangular tank with inlets/outlets is depicted in Fig. 3. As shown in this figure, the inlets/outlets illustrated in gray cells are located symmetrically along the *x*-direction and *y*-direction boundaries. The proposed symmetric arrangement of the inlets/outlets is posed by the Casimir property of the shallow-water bracket to be satisfied in our computational modelling. The boundary lines, i.e. the rigid porous side walls, are the coordinate lines $\{(x,y):\,x=0,L_1\}$ in the *x*-direction, and $\{(x,y):\,y=0,L_2\}$ in the *y*-direction. The horizontal fluid velocity denoted by $u_k$ is prescribed at the inlets/outlets along the *x*-direction boundaries, and the vertical velocity denoted by $v_l$ is prescribed at the inlets/outlets along the *y*-direction boundaries. For the FlexSlosh WEC problem, there may exist multiple inlets/outlets along the rigid side walls. Furthermore, their arrangement may differ from the schematic shown in Fig. 3, i.e. the inlets/outlets along the *x*-direction and *y*-directions boundaries can be asymmetrical with non-periodic boundary conditions (see Fig. 4 and §3.2 for more details).

We note that the $\alpha _{\textbf{n},\textbf{m}}$, $\beta _{\textbf{n},\textbf{m}}$ and $\gamma _{\textbf{n},\textbf{m}}$ weights in the fourth-order C-bracket discretisation (as defined in Fig. 2) — for a finite domain with periodic boundary conditions — are obtained by enforcing the conditions in (2.13b) for the conservation of the discrete potential enstrophy *P* using the discrete version of the shallow water Poisson-bracket (1.15) in (2.4). But in the presence of *solid boundaries with inlets/outlets* (porous side walls) and *combined no-flow and inflow-outflow boundary conditions*, if we use the same $\alpha _{\textbf{n},\textbf{m}}$, $\beta _{\textbf{n},\textbf{m}}$ and $\gamma _{\textbf{n},\textbf{m}}$ weights in the numerical scheme, i.e. using the discrete version of the shallow water Poisson-bracket (1.15) instead of discretising the bracket (1.14) with the boundary integral term on the right-hand side, then to satisfy the potential-enstrophy-conserving property of the shallow-water bracket, we should look into the *boundary integrals* that emerge from integration by parts (at continuous level) in enforcement of the Casimir property

3.1a $$\begin{aligned} \displaystyle \{\widetilde{u}_0\,,\mathcal {P}\}&= 0 \,,\end{aligned}$$

3.1b $$\begin{aligned} \displaystyle \{\widetilde{v}_0\,,\mathcal {P}\}&= 0 \,,\end{aligned}$$

3.1c $$\begin{aligned} \displaystyle \{h_0\,,\mathcal {P}\}&= 0 \,, \end{aligned}$$

where $\widetilde{u}_0=\widetilde{u}\left( \boldsymbol{x}_0,t\right)$, $\widetilde{v}_0=\widetilde{v}\left( \boldsymbol{x}_0,t\right)$, and $h_0=h\left( \boldsymbol{x}_0,t\right)$, and ensure that they are vanished using appropriate boundary conditions. The boundary integrals give rise to the boundary conditions and ghost field variables values required in the fourth-order C-bracket discretisation, with the given $\alpha _{\textbf{n},\textbf{m}}$, $\beta _{\textbf{n},\textbf{m}}$ and $\gamma _{\textbf{n},\textbf{m}}$ weights as in Fig. 2, for the sloshing problem with inflow-outflow mass fluxes. We also note that the total mass $\mathcal {M}$ satisfies the Casimir property $\{\widetilde{u}_0\,,\mathcal {M}\}=\{\widetilde{v}_0\,,\mathcal {M}\} =\{h_0\,,\mathcal {M}\}=0$ using the shallow water Poisson-bracket (1.15), or using the bracket (1.14) because the boundary integral vanishes.

For the sloshing problem in the FlexSlosh WEC with porous side walls, we are interested in preserving the total amount of mass in the system. Here, we also look into the bracket form of the equation for the mass conservation based on the antisymmetric shallow water bracket (1.15) and the boundary terms that emerge using the divergence theorem. The evolution equation for the fluid depth $h_0=h\left( \boldsymbol{x}_0,t\right)$ using the shallow water Poisson-bracket (1.15) takes the form

3.2 $$\begin{aligned} \begin{array}{rcl} \displaystyle \frac{dh_0}{dt} = \{h_0\,,\,\mathcal {H}\} & =& \displaystyle -\iint _{\textsf{D}}\,d\boldsymbol{x}\,\,\delta \left( \boldsymbol{x}-\boldsymbol{x}_0\right) \boldsymbol{\nabla }\boldsymbol{\cdot }\left( h\boldsymbol{u}\right) \\ & & \displaystyle +\int \,dy\,\big (\delta \left( \boldsymbol{x}-\boldsymbol{x}_0\right) hu\big ) \bigg |_{\boldsymbol{x}=\left( 0,y\right) }^{\boldsymbol{x}=\left( L_1,y\right) }\\ & & +\int \,dx\,\big (\delta \left( \boldsymbol{x}-\boldsymbol{x}_0\right) hv\big ) \bigg |_{\boldsymbol{x}=\left( x,0\right) }^{\boldsymbol{x}=\left( x,L_2\right) } \\ & =& \displaystyle -\boldsymbol{\nabla }\boldsymbol{\cdot }\left( h\boldsymbol{u}\right) \bigg |_{\boldsymbol{x}=\boldsymbol{x}_0} +hu\left( L_1,y_0,t\right) -hu\left( 0,y_0,t\right) \\ & & \displaystyle +hv\left( x_0,L_2,t\right) -hv\left( x_0,0,t\right) \,, \end{array} \end{aligned}$$

for $0\le x_0\le L_1$ and $0\le y_0\le L_2$. The second term, the third term, the fourth term, and the last term on the right-hand side of (3.2) are potentially nonzero for the points $\boldsymbol{x}_0$ on the boundary lines $\{\left( x_0,y_0\right) :\,x_0=L_1\}$, $\{\left( x_0,y_0\right) :\,x_0=0\}$, $\{\left( x_0,y_0\right) :\,y_0=L_2\}$, and $\{\left( x_0,y_0\right) :\,y_0=0\}$, respectively. Hence, in order to satisfy the mass conservation equation and preserve the total amount of mass in the sloshing system with porous side walls, we enforce the following boundary conditions:

(i) The no-flow or sloshing boundary conditions for the fluid velocity along the solid walls, i.e.
  3.3 $$\begin{aligned} u\left( 0,y,t\right) =u\left( L_1,y,t\right) =0 \quad \text{ and }\quad v\left( x,0,t\right) =v\left( x,L_2,t\right) =0\,. \end{aligned}$$
(ii) The periodic inflow-outflow boundary conditions for the *u* grid points at the inlets/outlets along the *x*-direction boundaries $\{\left( x,y\right) :\,x=0,L_1\}$, and for the *v* grid points at the inlets/outlets along the *y*-direction boundaries $\{\left( x,y\right) :\,y=0,L_2\}$, i.e.
  3.4 $$\begin{aligned} hu\left( 0,y,t\right) =hu\left( L_1,y,t\right) \quad \text{ and }\quad hv\left( x,0,t\right) =hv\left( x,L_2,t\right) \,. \end{aligned}$$

To enforce the periodic inflow-outflow boundary conditions in (3.4), first take a prescribed horizontal velocity of the form

3.5 $$\begin{aligned} u_k\left( 0,y_k,t\right) =u_k\left( L_1,y_k,t\right) = \epsilon _k\cos \omega _kt \,,\,\,\, 1\le k\le \textrm{I} \,, \end{aligned}$$

for the *u* grid points at the *x*-direction inlets/outlets, i.e. at the centre of the gray grid cells along the *x*-direction boundaries, as depicted in Fig. 3, with coordinates $\left( 0,y_k\right)$ and $\left( L_1,y_k\right)$, where $\textrm{I}$ is a positive integer representing the total number of inlets/outlets along the left/right boundary line, $\epsilon _k$ is a given amplitude, and $\omega _k$ is a given frequency. There may exist multiple inlets/outlets along the *x*-direction boundaries (see the numerical simulation in §4.3). Now, to satisfy the *x*-direction boundary condition in (3.4), i.e. $hu_k\left( 0,y_k,t\right) =hu_k\left( L_1,y_k,t\right)$, we impose a periodic boundary condition for the wave height *h* at the exterior/ghost *h* grid points (a) to the left of the boundary grid points with prescribed $u_k\left( 0,y_k,t\right)$, and (b) to the right of the boundary grid points with prescribed $u_k\left( L_1,y_k,t\right)$, i.e.

3.6 $$\begin{aligned} h\left( -d/2,y_k,t\right) = h\left( L_1-d/2,y_k,t\right) \quad \text{ and }\quad h\left( L_1+d/2,y_k,t\right) = h\left( d/2,y_k,t\right) \,. \end{aligned}$$

Next, take a prescribed horizontal velocity of the form

3.7 $$\begin{aligned} v_l\left( x_l,0,t\right) =v_l\left( x_l,L_2,t\right) = \epsilon _l^\prime \cos \omega _l^\prime t \,,\,\,\, 1\le l\le \textrm{J} \,, \end{aligned}$$

for the *v* grid points at the *y*-direction inlets/outlets, i.e. at the centre of the gray grid cells along the *y*-direction boundaries, as depicted in Fig. 3, with coordinates $\left( x_l,0\right)$ and $\left( x_l,L_2\right)$, where $\textrm{J}$ is a positive integer representing the total number of inlets/outlets along the bottom/top boundary line, $\epsilon _l^\prime$ is a given amplitude, and $\omega _l^\prime$ is a given frequency. Similarly, there may exist multiple inlets/outlets along the *y*-direction boundaries. To satisfy the *y*-direction boundary condition in (3.4), i.e. $hv_l\left( x_l,0,t\right) =hv_l\left( x_l,L_2,t\right)$, we impose a periodic boundary condition for the wave height *h* at the exterior/ghost *h* grid points (c) below the boundary grid points with prescribed $v_l\left( x_l,0,t\right)$, and (d) above the boundary grid points with prescribed $v_l\left( x_l,L_2,t\right)$, i.e.

3.8 $$\begin{aligned} h\left( x_l,-d/2,t\right) = h\left( x_l,L_2-d/2,t\right) \quad \text{ and }\quad h\left( x_l,L_2+d/2,t\right) = h\left( x_l,d/2,t\right) \,. \end{aligned}$$

In addition, we use the boundary integrals that arise from the enforcement of the potential-enstrophy-conserving property of the shallow-water Poisson bracket in (3.1) to define the boundary condition for the $\mathcal {V}$ grid points along the porous boundaries, and calculate the ghost field variables values in the context of the fourth-order C-bracket discretisation, for the following cases:

1. ***Inflow-outflow related grid points in the*** *x*-***direction***. Calculate the ghost field variables values for the *h*, *v*, and $\mathcal {V}$ grid points to the left and right of the prescribed $u_k$ boundary grid points along the boundary lines x=0 and $x=L_1$, respectively. Moreover, find the boundary condition for the $\mathcal {V}$ grid points along the *x*-direction boundaries surrounding the prescribed $u_k$ boundary grid points – in other words, the $\mathcal {V}$ grid points surrounding the *porous* parts of the *x*-direction boundaries. These grid points are highlighted in Fig. 3. Explicitly, find the values of the following grid points:

**Figure:**
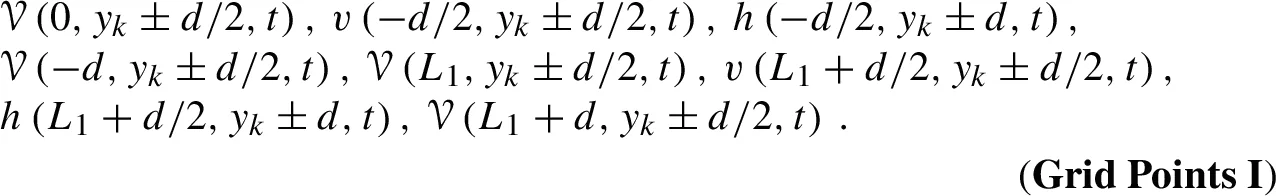
2. ***Inflow-outflow related grid points in the*** *y*-***direction***. Calculate the ghost field variables values for the *h*, *u*, and $\mathcal {V}$ grid points below and above the prescribed $v_l$ boundary grid points along the boundary lines y=0 and $y=L_2$, respectively. Moreover, find the boundary condition for the $\mathcal {V}$ grid points along the *y*-direction boundaries surrounding the prescribed $v_l$ boundary grid points – in other words, the $\mathcal {V}$ grid points surrounding the *porous* parts of the *y*-direction boundaries. These grid points are highlighted in Fig. 3. Explicitly, find the values of the following grid points:

**Figure:**
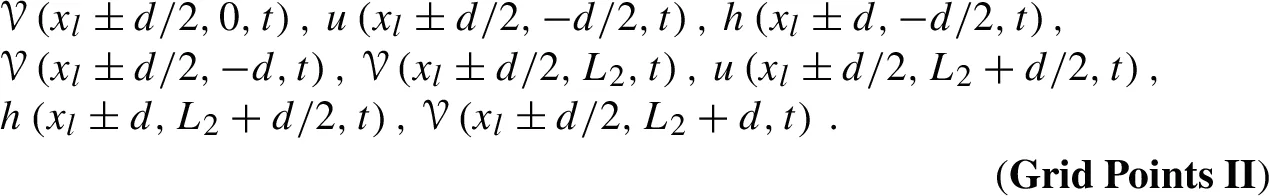
3. ***Sloshing related grid points along the solid side walls***. Calculate the ghost field variables values for the *h*, *v*, and $\mathcal {V}$ grid points to the left and right of the *solid* parts of the boundary lines x=0 and $x=L_1$, respectively. Moreover, calculate the ghost field variables values for the *h*, *u*, and $\mathcal {V}$ grid points below and above the *solid* parts of the boundary lines y=0 and $y=L_2$, respectively. Furthermore, find the boundary condition for the $\mathcal {V}$ grid points along the *solid* parts of the boundaries. See §3.1.3 for more details.

#### Inflow-outflow boundary conditions for the *x*-direction boundaries

We use the Casimir property of the shallow-water Poisson-bracket in (3.1a) to derive the required boundary condition for the $\mathcal {V}$ grid points, and calculate the ghost grid points values in (**Grid Points I**). To satisfy (3.1a), using the shallow water Poisson-bracket (1.15), we need to have

3.9 $$\begin{aligned} \begin{array}{rcl} \{\widetilde{u}_0\,,\mathcal {P}\}= &  \displaystyle \iint \,d\boldsymbol{x}\,\,\delta \left( \boldsymbol{x}-\boldsymbol{x}_0\right) \left( \mathcal {V}\,\frac{\delta \mathcal {P}}{\delta \widetilde{v}} -\frac{\partial \,}{\partial x}\left( \frac{\delta \mathcal {P}}{\delta h}\right) \right) = 0\,. \end{array} \end{aligned}$$

The variations in (3.9) take the form

3.10 $$\begin{aligned} \frac{\delta \mathcal {P}}{\delta h} = -\iint \, d\boldsymbol{x}\,\left( \frac{1}{2}\mathcal {V}^2\delta h\right) \,, \end{aligned}$$

for an arbitrary variation δh, and

3.11 $$\begin{aligned} \begin{array}{rcl} \displaystyle \frac{\delta \mathcal {P}}{\delta \widetilde{v}} & =& \displaystyle \frac{\delta \mathcal {P}}{\delta v} = \frac{d\,}{d\varepsilon }\bigg |_{\varepsilon =0}\iint \,d\boldsymbol{x}\, \left( \frac{1}{2}\frac{1}{h}\left( v_x+\varepsilon \delta v_x-u_y\right) ^2\right) \\ & =& \displaystyle -\iint \,d\boldsymbol{x}\,\left( \frac{\partial \mathcal {V}}{\partial x}\,\delta v\right) +\int \,dy\,\big (\left( \mathcal {V}\,\delta v\right) \left( L_1,y,t\right) -\left( \mathcal {V}\,\delta v\right) \left( 0,y,t\right) \big )\,, \end{array} \end{aligned}$$

for an arbitrary variation δv. Hence (3.9) is satisfied if we impose the *periodic boundary condition*

3.12 $$\begin{aligned} \left( \mathcal {V}\delta v\right) \left( 0,y,t\right) =\left( \mathcal {V}\delta v\right) \left( L_1,y,t\right) \,, \end{aligned}$$

in the *boundary integral* on the right-hand side of (3.11).

Note that $\{\widetilde{u}_0,\mathcal {P}\}$ with the bracket (1.14) leads to the same expression on the right-hand side in (3.9) because the boundary integral in (1.14) with $\mathcal {A}=\widetilde{u}_0$ vanishes.

To satisfy the boundary condition (3.12) for the $\mathcal {V}$ grid points surrounding the inlets/outlets along the *x*-direction boundaries, with the coordinates $\left( 0,y_k\pm d/2\right)$ and $\left( L_1,y_k\pm d/2\right)$, i.e.

3.13 $$\begin{aligned} \left( \mathcal {V}\delta v\right) \left( 0,y_k\pm d/2,t\right) =\left( \mathcal {V}\delta v\right) \left( L_1,y_k\pm d/2,t\right) \,, \end{aligned}$$

we first impose

3.14 $$\begin{aligned} \delta v\left( 0,y_k\pm d/2,t\right) = \delta v\left( L_1,y_k\pm d/2,t\right) \,, \end{aligned}$$

which gives the *periodic boundary condition* for the exterior/ghost *v* grid points:

3.15 $$\begin{aligned} \begin{array}{rcl} v\left( -d/2,y_k\pm d/2,t\right) & =& v\left( L_1-d/2,y_k\pm d/2,t\right) \quad \text{ and }\quad \\ v\left( L_1+d/2,y_k\pm d/2,t\right) & =& v\left( d/2,y_k\pm d/2,t\right) \,. \end{array} \end{aligned}$$

Another possible choice is to impose a zero boundary condition

3.16 $$\begin{aligned} \delta v\left( 0,y_k\pm d/2,t\right) = \delta v\left( L_1,y_k\pm d/2,t\right) = 0\,, \end{aligned}$$

which gives the *symmetric boundary condition* for *v*:

3.17 $$\begin{aligned} \begin{array}{rcl} v\left( -d/2,y_k\pm d/2,t\right) & =& v\left( d/2,y_k\pm d/2,t\right) \quad \text{ and }\quad \\ v\left( L_1+d/2,y_k\pm d/2,t\right) & =& v\left( L_1-d/2,y_k\pm d/2,t\right) \,. \end{array} \end{aligned}$$

Next, we impose the periodic boundary condition for $\mathcal {V}$ in (3.13), i.e.

3.18 $$\begin{aligned} \mathcal {V}\left( 0,y_k\pm d/2,t\right) =\mathcal {V}\left( L_1,y_k\pm d/2,t\right) \,, \end{aligned}$$

and so using (2.9b) we get

3.19 $$\begin{aligned} \begin{array}{rcl} & & \displaystyle \frac{v\left( d/2,y_k\pm d/2,t\right) -v\left( -d/2,y_k\pm d/2,t\right) \pm u_k\left( 0,y_k,t\right) \mp u\left( 0,y_k\pm d,t\right) }{h\left( d/2,y_k,t\right) +h\left( -d/2,y_k,t\right) +h\left( d/2,y_k\pm d,t\right) +h\left( -d/2,y_k\pm d,t\right) } \\ & & \quad \displaystyle = \frac{v\left( L_1+d/2,y_k\pm d/2,t\right) -v\left( L_1-d/2,y_k\pm d/2,t\right) \pm u_k\left( L_1,y_k,t\right) \mp u\left( L_1,y_k\pm d,t\right) }{h\left( L_1+d/2,y_k,t\right) +h\left( L_1-d/2,y_k,t\right) +h\left( L_1+d/2,y_k\pm d,t\right) +h\left( L_1-d/2,y_k\pm d,t\right) }\,. \end{array} \end{aligned}$$

Substitution of the no-flow boundary conditions $u\left( 0,y_k\pm d,t\right) =u\left( L_1,y_k\pm d,t\right) =0$, the prescribed velocity $u_k$ given in (3.5), and the periodic boundary conditions (3.6) and (3.15) (or the symmetric boundary condition (3.17) for *v*) in (3.19) gives the *periodic boundary condition* for the exterior/ghost *h* grid points in the *x*-direction:

3.20 $$\begin{aligned} \begin{array}{rcl} h\left( -d/2,y_k\pm d,t\right) & =& h\left( L_1-d/2,y_k\pm d,t\right) \quad \text{ and }\quad \\ h\left( L_1+d/2,y_k\pm d,t\right) & =& h\left( d/2,y_k\pm d,t\right) \,. \end{array} \end{aligned}$$

**Remark 3.1.** ***Justification of*** (3.18)-(3.20) ***with*** δv=0. If we impose the zero boundary condition for δv in (3.16), then the boundary condition (3.13) is satisfied, and so it is not required to further impose the periodic boundary condition for $\mathcal {V}$ in (3.18). However, to satisfy *global conservation* of the Casimir property in (3.1c) at continuous level, the following periodic boundary conditions must be satisfied:

3.21a $$\begin{aligned} \displaystyle \frac{\partial \mathcal {V}}{\partial y}\left( 0,y,t\right)&= \displaystyle \frac{\partial \mathcal {V}}{\partial y}\left( L_1,y,t\right) \,, \end{aligned}$$

3.21b $$\begin{aligned} \displaystyle \frac{\partial \mathcal {V}}{\partial x}\left( x,0,t\right)&= \displaystyle \frac{\partial \mathcal {V}}{\partial x}\left( x,L_2,t\right) \,. \end{aligned}$$

But, at discrete level, the boundary condition (3.21a) is satisfied if the boundary conditions (3.18) and so (3.20) hold, taking into account that we use a *zero-potential-vorticity* boundary condition for the $\mathcal {V}$ grid points along the solid boundaries (see §3.1.3). Note that the boundary conditions in (3.21) emerge from the *boundary integrals* in enforcement of the Casimir property (3.1c) using the shallow water Poisson-bracket (1.15). The proof is given below:

3.22 $$\begin{aligned} \begin{array}{rcl} \{h_0\,,\mathcal {P}\} & =& \displaystyle \iint \, d\boldsymbol{x}\,\frac{\delta \mathcal {P}}{\delta \widetilde{\boldsymbol{u}}}\boldsymbol{\cdot }\boldsymbol{\nabla }\,\frac{\delta h_0}{\delta h} \\ & =& \displaystyle \iint \, d\boldsymbol{x}\,\left( \boldsymbol{\nabla }\boldsymbol{\cdot }\left( \frac{\delta \mathcal {P}}{\delta \widetilde{\boldsymbol{u}}}\,\delta \left( \boldsymbol{x}-\boldsymbol{x}_0\right) \right) -\delta \left( \boldsymbol{x}-\boldsymbol{x}_0\right) \boldsymbol{\nabla }\boldsymbol{\cdot }\frac{\delta \mathcal {P}}{\delta \widetilde{\boldsymbol{u}}}\right) \\ & =& \displaystyle -\left( \boldsymbol{\nabla }\boldsymbol{\cdot }\frac{\delta \mathcal {P}}{\delta \widetilde{\boldsymbol{u}}}\right) \bigg |_{\boldsymbol{x}=\boldsymbol{x}_0} +\int \, dy\,\,\mathcal {V}_y\,\delta \left( \boldsymbol{x}-\boldsymbol{x}_0\right) \bigg |_{\boldsymbol{x}=(0,y)}^{\boldsymbol{x}=(L_1,y)} \\ & & \displaystyle -\int \, dx\,\,\mathcal {V}_x\,\delta \left( \boldsymbol{x}-\boldsymbol{x}_0\right) \bigg |_{\boldsymbol{x}=(x,0)}^{\boldsymbol{x}=(x,L_2)} \\ & =& \displaystyle -\left( \boldsymbol{\nabla }\boldsymbol{\cdot }\frac{\delta \mathcal {P}}{\delta \widetilde{\boldsymbol{u}}}\right) \bigg |_{\boldsymbol{x}=\boldsymbol{x}_0} +\mathcal {V}_y\left( L_1,y_0,t\right) -\mathcal {V}_y\left( 0,y_0,t\right) \\ & & \displaystyle -\mathcal {V}_x\left( x_0,L_2,t\right) +\mathcal {V}_x\left( x_0,0,t\right) \,, \end{array} \end{aligned}$$

for $0\le x_0\le L_1$ and $0\le y_0\le L_2$. The second term, the third term, the fourth term, and the last term on the right-hand side of (3.22) are potentially nonzero for the points $\boldsymbol{x}_0$ on the boundary lines $\{\left( x_0,y_0\right) :\,x_0=L_1\}$, $\{\left( x_0,y_0\right) :\,x_0=0\}$, $\{\left( x_0,y_0\right) :\,y_0=L_2\}$, and $\{\left( x_0,y_0\right) :\,y_0=0\}$, respectively. Hence, using (3.18) and (3.20), global conservation of the Casimir property in (3.1c) at discrete level is satisfied, i.e. discrete integration over all spatial grid points, as the terms on the right-hand side of (3.22) cancel out when summed over all grid points along the boundaries. See also Remark 3.2 and Remark 3.3. In (3.22) we use (3.11) and (3.26) for $\delta \mathcal {P}/\delta \widetilde{\boldsymbol{u}}$. We also note that using the boundary conditions (3.18) and (3.33) is in harmony with the periodic boundary condition required to satisfy the potential vorticity equation in (B.5) (see Appendix B) based on the shallow water Poisson-bracket (1.15).

It is shown in Appendix C that $\{h_0,\mathcal {P}\}=0$ with the bracket (1.14) because the boundary terms are cancelled out. As we use the shallow water Poisson-bracket (1.15) the boundary conditions (3.18), (3.33), and (3.55) are imposed to satisfy the potential-enstrophy-conserving property of the numerical scheme with the no-flow and inflow-outflow boundary conditions.

Using the fourth-order Poisson-bracket discretisations (2.14), (2.15), (2.16), the values of the exterior/ghost grid points $\mathcal {V}\left( -d,y_k\pm d/2,t\right)$ and $\mathcal {V}\left( L_1+d,y_k\pm d/2,t\right)$ are required to calculate the interior grid points $v\left( d/2,y_k\pm d/2,t\right)$ and $v\left( L_1-d/2,\right. \left. y_k\pm d/2,t\right)$, respectively. Since we have used the periodic boundary condition (3.18) for the $\mathcal {V}$ grid points surrounding the porous parts of the boundary, one possible choice is to use the periodic boundary condition

3.23 $$\begin{aligned} \begin{array}{rcl} \mathcal {V}\left( -d,y_k\pm d/2,t\right) & =& \mathcal {V}\left( L_1-d,y_k\pm d/2,t\right) \quad \text{ and }\quad \\ \mathcal {V}\left( L_1+d,y_k\pm d/2,t\right) & =& \mathcal {V}\left( d,y_k\pm d/2,t\right) \,. \end{array} \end{aligned}$$

Another possible choice is to use the discrete approximations

3.24 $$\begin{aligned} \begin{array}{rcl} \mathcal {V}\left( -d,y_k\pm d/2,t\right) & =& 2\mathcal {V}\left( 0,y_k\pm d/2,t\right) -\mathcal {V}\left( d,y_k\pm d/2,t\right) \,,\\ \mathcal {V}\left( L_1+d,y_k\pm d/2,t\right) & =& 2\mathcal {V}\left( L_1,y_k\pm d/2,t\right) -\mathcal {V}\left( L_1-d,y_k\pm d/2,t\right) \,. \end{array} \end{aligned}$$

We note that the choice of (3.23) or (3.24) does not affect the conservation properties discussed above. This completes the derivation and justification of the inflow-outflow boundary conditions for the grid points in (**Grid Points I**).

#### Inflow-outflow boundary conditions for the *y*-direction boundaries

We use the Casimir property of the shallow-water Poisson-bracket in (3.1b) to derive the required boundary condition for the $\mathcal {V}$ grid points, and calculate the ghost grid points values in (**Grid Points II**). To satisfy (3.1b), using the shallow water Poisson-bracket (1.15), we need to have

3.25 $$\begin{aligned} \begin{array}{rcl} \{\widetilde{v}_0\,,\mathcal {P}\}= &  \displaystyle \iint \,d\boldsymbol{x}\,\, \delta \left( \boldsymbol{x}-\boldsymbol{x}_0\right) \left( -\mathcal {V}\,\frac{\delta \mathcal {P}}{\delta \widetilde{u}} -\frac{\partial \,}{\partial y}\left( \frac{\delta \mathcal {P}}{\delta h}\right) \right) = 0\,. \end{array} \end{aligned}$$

The variations in (3.25) take the form (3.10) and

3.26 $$\begin{aligned} \begin{array}{rcl} \displaystyle \frac{\delta \mathcal {P}}{\delta \widetilde{u}} & =& \displaystyle \frac{\delta \mathcal {P}}{\delta u} = \frac{d\,}{d\varepsilon }\bigg |_{\varepsilon =0}\iint \,d\boldsymbol{x}\, \left( \frac{1}{2}\frac{1}{h}\left( v_x-u_y-\varepsilon \delta u_y\right) ^2\right) \\ & =& \displaystyle \iint \,d\boldsymbol{x}\,\left( \frac{\partial \mathcal {V}}{\partial y}\,\delta u\right) -\int \,dx\,\big (\left( \mathcal {V}\,\delta u\right) \left( x,L_2,t\right) -\left( \mathcal {V}\,\delta u\right) \left( x,0,t\right) \big )\,, \end{array} \end{aligned}$$

for an arbitrary variation δu. Hence (3.25) is satisfied if we impose the *periodic boundary condition*

3.27 $$\begin{aligned} \left( \mathcal {V}\delta u\right) \left( x,0,t\right) =\left( \mathcal {V}\delta u\right) \left( x,L_2,t\right) \,, \end{aligned}$$

in the *boundary integral* on the right-hand side of (3.26).

Note that $\{\widetilde{v}_0,\mathcal {P}\}$ with the bracket (1.14) leads to the same expression on the right-hand side in (3.25) because the boundary integral in (1.14) with $\mathcal {A}=\widetilde{v}_0$ vanishes.

To satisfy the boundary condition (3.27) for the $\mathcal {V}$ grid points surrounding the inlets/outlets along the *y*-direction boundaries, with the coordinates $\left( x_l\pm d/2,0\right)$ and $\left( x_l\pm d/2,L_2\right)$, i.e.

3.28 $$\begin{aligned} \left( \mathcal {V}\delta u\right) \left( x_l\pm d/2,0,t\right) =\left( \mathcal {V}\delta u\right) \left( x_l\pm d/2,L_2,t\right) \,, \end{aligned}$$

we first impose

3.29 $$\begin{aligned} \delta u\left( x_l\pm d/2,0,t\right) = \delta u\left( x_l\pm d/2,L_2,t\right) \,, \end{aligned}$$

which gives the *periodic boundary condition* for the exterior/ghost *u* grid points:

3.30 $$\begin{aligned} \begin{array}{rcl} u\left( x_l\pm d/2,-d/2,t\right) & =& u\left( x_l\pm d/2,L_2-d/2,t\right) \quad \text{ and }\quad \\ u\left( x_l\pm d/2,L_2+d/2,t\right) & =& u\left( x_l\pm d/2,d/2,t\right) \,. \end{array} \end{aligned}$$

Another possible choice is to impose a zero boundary condition

3.31 $$\begin{aligned} \delta u\left( x_l\pm d/2,0,t\right) = \delta u\left( x_l\pm d/2,L_2,t\right) = 0\,, \end{aligned}$$

which gives the *symmetric boundary condition* for *u*:

3.32 $$\begin{aligned} \begin{array}{rcl} u\left( x_l\pm d/2,-d/2,t\right) & =& u\left( x_l\pm d/2,d/2,t\right) \quad \text{ and }\quad \\ u\left( x_l\pm d/2,L_2+d/2,t\right) & =& u\left( x_l\pm d/2,L_2-d/2,t\right) \,. \end{array} \end{aligned}$$

Next, we impose the periodic boundary condition for $\mathcal {V}$ in (3.28), i.e.

3.33 $$\begin{aligned} \mathcal {V}\left( x_l\pm d/2,0,t\right) =\mathcal {V}\left( x_l\pm d/2,L_2,t\right) \,, \end{aligned}$$

and so using (2.9b) we get

3.34 $$\begin{aligned} \begin{array}{rcl} & & \displaystyle \frac{\pm v\left( x_l\pm d,0,t\right) \mp v_l\left( x_l,0,t\right) + u\left( x_l\pm d/2,-d/2,t\right) -u\left( x_l\pm d/2,d/2,t\right) }{h\left( x_l,d/2,t\right) +h\left( x_l,-d/2,t\right) +h\left( x_l\pm d,d/2,t\right) +h\left( x_l\pm d,-d/2,t\right) } \\ & & \displaystyle = \frac{\pm v\left( x_l\pm d,L_2,t\right) \mp v_l\left( x_l,L_2,t\right) + u\left( x_l\pm d/2,L_2-d/2,t\right) -u\left( x_l\pm d/2,L_2+d/2,t\right) }{h\left( x_l,L_2+d/2,t\right) +h\left( x_l,L_2-d/2,t\right) +h\left( x_l\pm d,L_2+d/2,t\right) +h\left( x_l\pm d,L_2-d/2,t\right) }\,. \end{array} \end{aligned}$$

Substitution of the no-flow boundary conditions $v\left( x_l\pm d,0,t\right) =v\left( x_l\pm d,L_2,t\right) =0$, the prescribed velocity $v_l$ given in (3.7), and the periodic boundary conditions (3.8) and (3.30) (or the symmetric boundary condition (3.32) for *u*) in (3.34) gives the *periodic boundary condition* for the exterior/ghost *h* grid points in the *y*-direction:

3.35 $$\begin{aligned} \begin{array}{rcl} h\left( x_l\pm d,-d/2,t\right) & =& h\left( x_l\pm d,L_2-d/2,t\right) \quad \text{ and }\quad \\ h\left( x_l\pm d,L_2+d/2,t\right) & =& h\left( x_l\pm d,d/2,t\right) \,. \end{array} \end{aligned}$$

**Remark 3.2.** ***Justification of*** (3.33)-(3.35) ***with*** δu=0. If we impose the zero boundary condition for δu in (3.31), then the boundary condition (3.28) is satisfied, and so it is not required to further impose the periodic boundary condition for $\mathcal {V}$ in (3.33). However, to satisfy global conservation of the Casimir property in (3.1c) at discrete level, the boundary condition in (3.21b) must be satisfied, which means the boundary conditions (3.33) and so (3.35) must hold, taking into account that we use a *zero-potential-vorticity* boundary condition for the $\mathcal {V}$ grid points along the solid boundaries.

Similarly, using the fourth-order Poisson-bracket discretisations (2.14), (2.15), (2.16), the values of the exterior/ghost grid points $\mathcal {V}\left( x_l\pm d/2,-d,t\right)$ and $\mathcal {V}\left( x_l\pm d/2,\right. \left. L_2+d,t\right)$ are required to calculate the interior grid points $u\left( x_l\pm d/2,d/2,t\right)$ and $u\left( x_l\pm d/2,L_2-d/2,t\right)$, respectively. Since we have used the periodic boundary condition (3.33) for the $\mathcal {V}$ grid points surrounding the porous parts of the boundary, one possible choice is to use the periodic boundary condition

3.36 $$\begin{aligned} \begin{array}{rcl} \mathcal {V}\left( x_l\pm d/2,-d,t\right) & =& \mathcal {V}\left( x_l\pm d/2,L_2-d,t\right) \quad \text{ and }\quad \\ \mathcal {V}\left( x_l\pm d/2,L_2+d,t\right) & =& \mathcal {V}\left( x_l\pm d/2,d,t\right) \,. \end{array} \end{aligned}$$

Another possible choice is to use the discrete approximations

3.37 $$\begin{aligned} \begin{array}{rcl} \mathcal {V}\left( x_l\pm d/2,-d,t\right) & =& 2\mathcal {V}\left( x_l\pm d/2,0,t\right) -\mathcal {V}\left( x_l\pm d/2,d,t\right) \,,\\ \mathcal {V}\left( x_l\pm d/2,L_2+d,t\right) & =& 2\mathcal {V}\left( x_l\pm d/2,L_2,t\right) -\mathcal {V}\left( x_l\pm d/2,L_2-d,t\right) \,. \end{array} \end{aligned}$$

We note that the choice of (3.36) or (3.37) does not affect the conservation properties discussed above. This completes the derivation and justification of the inflow-outflow boundary conditions for the grid points in (**Grid Points II**).

#### Sloshing boundary conditions for the rigid boundaries

In this section we introduce a similar approach to obtain the boundary conditions required, in the context of the fourth-order C-bracket discretisation, at solid parts of the boundaries. In addition to the no-flow boundary conditions (3.3), we need to define the boundary condition for the $\mathcal {V}$ grid points, and the exterior/ghost field variables in the *x*-direction:

**Figure:**
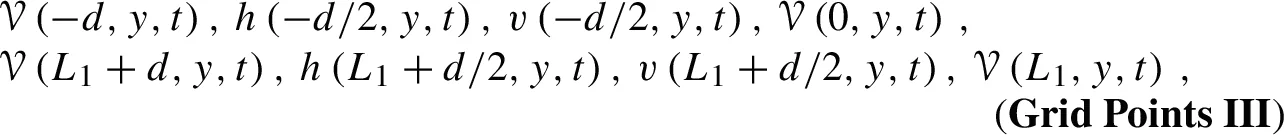

and the following grid points in the *y*-direction:

**Figure:**
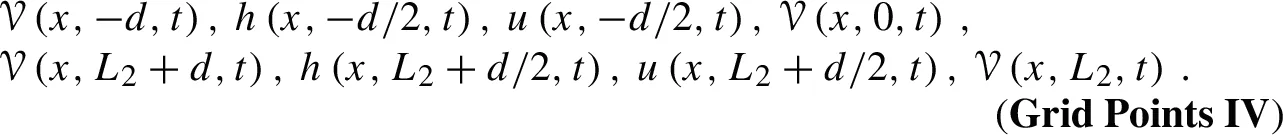

In order to satisfy the potential-enstrophy-conserving property of the shallow-water Poisson-bracket, the boundary conditions (3.12) and (3.27) must hold. For the *x*-direction boundaries, using a periodic boundary condition for δv in (3.12), in combination with the boundary conditions (3.43) and (3.44) for the wave height, does not give a periodic boundary condition for $\mathcal {V}$ in (3.12). Hence, to satisfy (3.12), one possible choice is imposition of

3.38 $$\begin{aligned} \delta v\left( 0,y,t\right) = \delta v\left( L_1,y,t\right) = 0\,, \end{aligned}$$

which gives the *symmetric* boundary condition

3.39 $$\begin{aligned} v\left( -d/2,y,t\right) = v\left( d/2,y,t\right) \quad \text{ and }\quad v\left( L_1+d/2,y,t\right) = v\left( L_1-d/2,y,t\right) \,. \end{aligned}$$

This boundary condition, in combination with the no-flow boundary condition for *u*, gives a *zero potential vorticity* along the *x*-direction boundaries, i.e.

3.40 $$\begin{aligned} \mathcal {V}\left( 0,y,t\right) = \mathcal {V}\left( L_1,y,t\right) = 0\,. \end{aligned}$$

The boundary condition (3.40) can be used to obtain

3.41 $$\begin{aligned} \mathcal {V}\left( -d,y,t\right) = -\mathcal {V}\left( d,y,t\right) \quad \text{ and }\quad \mathcal {V}\left( L_1+d,y,t\right) = -\mathcal {V}\left( L_1-d,y,t\right) \,. \end{aligned}$$

The discrete boundary condition for the wave height can be obtained from the reduced version of the momentum equations in (1.2). Using the boundary conditions (3.3) along the boundary lines $\{\left( x,y\right) :\,x=0,L_1\}$ where $u=u_y=0$, the *x*-momentum equation in the SWEs (1.2) reduces to

3.42 $$\begin{aligned} \begin{array}{rcl} g\left( h+h_b\right) _x= &  -\ddot{q}_1 \,, \end{array} \end{aligned}$$

where $\ddot{q}_1$ is the second time derivative of the perscribed surge motion $q_1(t)$ of the rectangular basin. We set $\partial h_b/\partial x=0$ in (3.42). Then the reduced equation (3.42) for the left boundary line, where x=0, gives

3.43 $$\begin{aligned} h\left( -d/2,y,t\right) = h\left( d/2,y,t\right) +\frac{d}{g}\,\ddot{q}_1 \,, \end{aligned}$$

and for the right boundary line, where $x=L_1$, gives

3.44 $$\begin{aligned} h\left( L_1+d/2,y,t\right) = h\left( L_1-d/2,y,t\right) -\frac{d}{g}\,\ddot{q}_1\,. \end{aligned}$$

This completes the derivation and justification of the sloshing boundary conditions for the grid points in (**Grid Points III**).

Similarly, for the *y*-direction boundaries, one possible choice is imposition of

3.45 $$\begin{aligned} \delta u\left( x,0,t\right) = \delta u\left( x,L_2,t\right) = 0\,, \end{aligned}$$

in (3.27) which gives the *symmetric* boundary condition

3.46 $$\begin{aligned} u\left( x,-d/2,t\right) = u\left( x,d/2,t\right) \quad \text{ and }\quad u\left( x,L_2+d/2,t\right) = u\left( x,L_2-d/2,t\right) \,. \end{aligned}$$

This further gives a *zero potential vorticity* along the *y*-direction boundaries, i.e.

3.47 $$\begin{aligned} \mathcal {V}\left( x,0,t\right) = \mathcal {V}\left( x,L_2,t\right) = 0\,. \end{aligned}$$

This boundary condition can be used to obtain

3.48 $$\begin{aligned} \mathcal {V}\left( x,-d,t\right) = -\mathcal {V}\left( x,d,t\right) \quad \text{ and }\quad \mathcal {V}\left( x,L_2+d,t\right) = -\mathcal {V}\left( x,L_2-d,t\right) \,. \end{aligned}$$

Next, using the boundary conditions (3.3) along the boundary lines $\{\left( x,y\right) :\,y=0,L_2\}$ where $v=v_x=0$, the *y*-momentum equation in the SWEs (1.2) reduces to

3.49 $$\begin{aligned} \begin{array}{rcl} g\left( h+h_b\right) _y= &  -\ddot{q}_2 \,, \end{array} \end{aligned}$$

where $\ddot{q}_2$ is the second time derivative of the perscribed sway motion $q_2(t)$. We set $\partial h_b/\partial y=0$ in (3.49). Then the reduced equation (3.49) for the bottom boundary line, where y=0, gives

3.50 $$\begin{aligned} h\left( x,-d/2,t\right) = h\left( x,d/2,t\right) +\frac{d}{g}\,\ddot{q}_2 \,, \end{aligned}$$

and for the top boundary line, where $y=L_2$, gives

3.51 $$\begin{aligned} h\left( x,L_2+d/2,t\right) = h\left( x,L_2-d/2,t\right) -\frac{d}{g}\,\ddot{q}_2 \,. \end{aligned}$$

This completes the derivation and justification of the sloshing boundary conditions for the grid points in (**Grid Points IV**).

**Remark 3.3.** We note that the boundary conditions (3.40) and (3.47) satisfy the conditions in (3.21a) and (3.21b), respectively. Moreover, they respect *local conservation* of the Casimir property in (3.1c), i.e. the right-hand side of (3.22) vanishes for the grid points along the solid walls. Hence, for the sloshing problem in a rigid tank *without* inlets/outlets, the conservation of the Casimir property in (3.1c) is both local and global. However, for the sloshing problem in a rigid tank with porous side walls, the conservation of the Casimir property in (3.1c) is only global, albeit in the special set-up proposed above.

### Non-symmetric porous boundaries with non-periodic inflow-outflow mass fluxes

The aim in this section is to extend the argument in §3.1 for an arbitrary distribution of the inlets/outlets along the solid boundaries. Fig. 4 shows the computational domain with non-symmetric porous side walls. The non-periodic inflow-outflow mass fluxes may be prescribed such that the total amount of mass in the sloshing system is preserved or not based on the modelling needs.

In order to simulate the sloshing problem in the FlexSlosh WEC for any symmetric or non-symmetric arrangement of the inlets/outlets with non-periodic inflow-outflow mass fluxes, the condition on *global conservation* of the Casimir property in (3.1c) *might be* relaxed, taking into account that (3.1c) is *locally* satisfied for all interior grid points and the boundary grid points which lie outside the stencil size centred at the inflow-outflow grid cell. For this problem, we use the no-flow boundary conditions (3.3), the boundary conditions (3.43), (3.44), (3.50), and (3.51) for the wave height obtained from the reduced version of the SWEs along the boundaries, and the following boundary conditions: 3.52a3.52b The boundary conditions (3.52a) and (3.52b) satisfy the conditions in (3.12) and (3.27), and so respect the Casimir property of the shallow-water Poisson bracket in (3.1a) and (3.1b), respectively. However, with these boundary conditions, global conservation of the Casimir property in (3.1c) is not necessarily satisfied unless the prescribed inflow-outflow velocities and the ghost *h* grid points values are defined such that the boundary terms on the right-hand side of (3.22) are cancelled out when summed over all boundary grid points (see also Remark 3.4). Note that the boundary conditions (3.52a) and (3.52b) lead to the symmetric boundary conditions (3.39) and (3.46), respectively. See §4 for the sloshing simulations in a tank with non-symmetric porous side walls and non-periodic inflow-outflow mass fluxes.

**Fig. 5:**
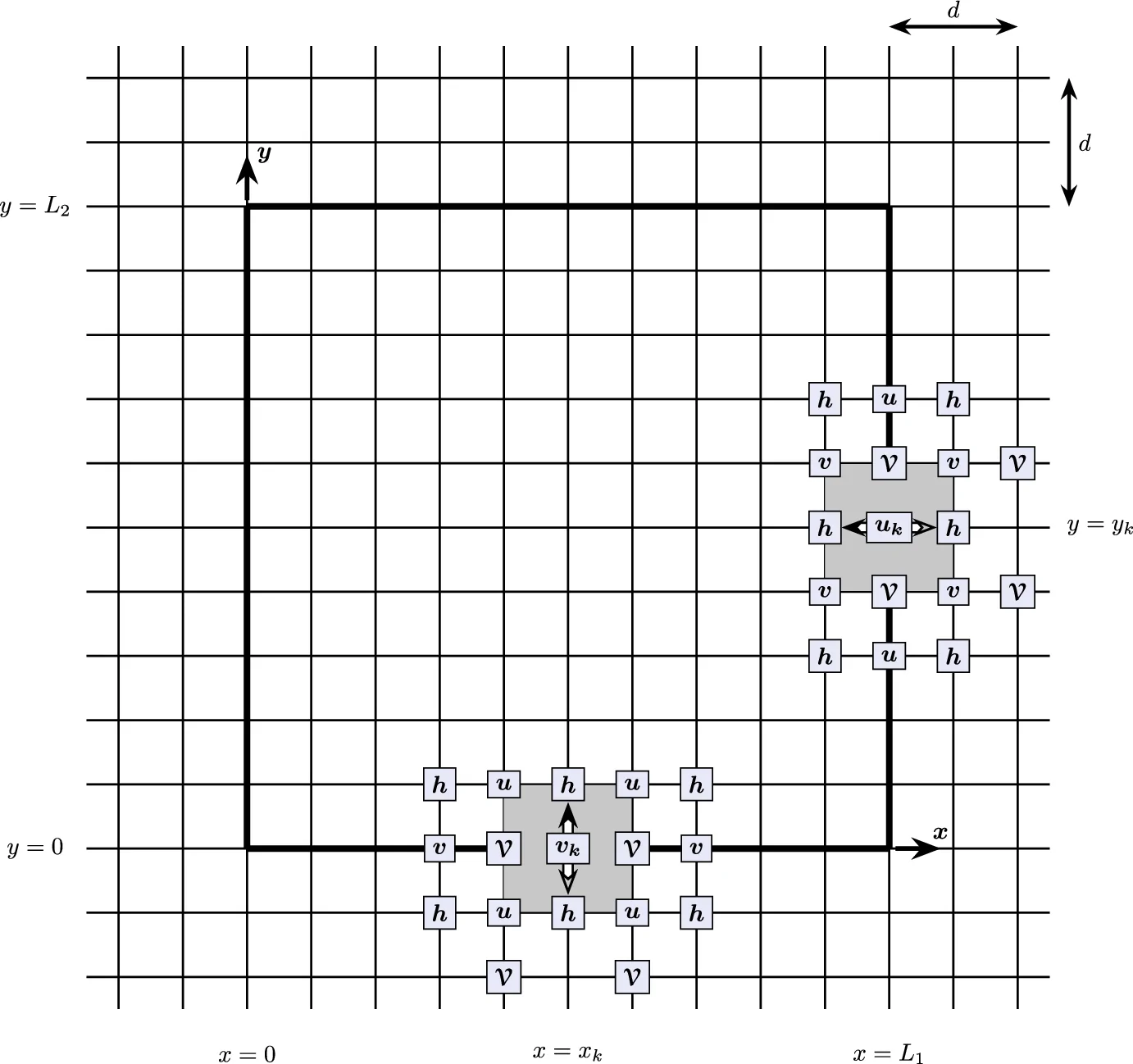
Computational domain with non-symmetric porous boundaries and non-periodic boundary conditions. Non-symmetric distribution of the inlets/outlets depicted in gray cells along the solid boundaries with the boundary conditions (3.53), (3.54) and (3.55) which respect global conservation of the Casimir property in (3.1c).

**Remark 3.4.** We note that *global conservation* of the Casimir property in (3.1c) can be satisfied for the sloshing problem with non-symmetric porous side walls if the prescribed inflow-outflow velocities $u_k$, $v_k$, and the boundary conditions for the ghost/exterior *h* grid points are defined such that the boundary terms on the right-hand side of (3.22) cancel out when summed over all boundary grid points. For example, consider the computational grid with porous bottom and right boundaries presented in Fig. 5. Take a prescribed horizontal velocity of the form

3.53 $$\begin{aligned} u_k\left( L_1,y_k,t\right) = v_k\left( x_k,0,t\right) = \epsilon _k\cos \omega _k t \,,\,\,\, 1\le k\le \textrm{I} \,, \end{aligned}$$

where $\textrm{I}$ is a positive integer representing the total number of inlets/outlets along the bottom/right boundary line, $\epsilon _k$ is a given amplitude, and $\omega _k$ is a given frequency. To preserve the total amount of mass in the sloshing system, we set

3.54 $$\begin{aligned} h\left( L_1+d/2,y_k,t\right) = h\left( x_k,d/2,t\right) \quad \text{ and }\quad h\left( x_k,-d/2,t\right) = h\left( L_1-d/2,y_k,t\right) \,. \end{aligned}$$

Next, to satisfy global conservation of the Casimir property in (3.1c), i.e. to vanish the right-hand side of (3.22) when summed over all grid points along the bottom and right boundary lines, we set

3.55 $$\begin{aligned} \begin{array}{rcl} h\left( L_1+d/2,y_k\pm d,t\right) & =& h\left( x_k\pm d,d/2,t\right) \quad \text{ and }\quad \\ h\left( x_k\pm d,-d/2,t\right) & =& h\left( L_1-d/2,y_k\pm d,t\right) \,. \end{array} \end{aligned}$$

Using the boundary conditions (3.53), (3.54), and (3.55) also ensures that the boundary terms on the right-hand side in (B.5) (see Appendix B) vanish.

See §4 for the numerical simulations with non-symmetric inlets/outlets and non-periodic boundary conditions.

## Structure-preserving sloshing simulations with inflow-outflow boundary conditions

In this section we present long-time structure-preserving numerical simulations for the problem of shallow-water sloshing in a rigid rectangular tank with symmetric porous side walls and periodic inflow-outflow boundary conditions, and for non-symmetric porous side walls with non-periodic inflow-outflow boundary conditions. The wave basin has a variable bottom surface and is under a prescribed coupled surge-sway motion.

The results in this section are inspired by the theory and experiment of Faltinsen *et al.* (2003) and the numerical simulations of Wu & Chen (2009) for fluid sloshing in a *rigid tank* with interior *finite water depth*. Our numerical simulations show that the *shallow-water* Poisson-bracket solver captures the essential features of square-like, diagonal, and swirling waves in a *porous* rigid tank with inflow-outflow mass fluxes and irregular bottom topography.

For the presented fourth-order C-bracket numerical simulations, the typical wall-clock time for 800s of a simulation on a 100×100 grid with $\Delta t=5\times 10^{-3}$ is about 2015s, coded in Matlab and run on a 64 bit 3.3 GHz 12-core Intel Xeon W processor, and the typical CPU time is about 3248s.

In the present study, a Von Neumann-type stability analysis has not been done for the discretised two-dimensional shallow-water equations. However, a CFL-type stability condition involving a $4^\textrm{th}$ power of the grid size *d* is expected due to the biharmonic dissipation terms (2.22) and (2.23). See §3.1.1 in Durran (1999) for stability analysis of the linearised one-dimensional shallow-water equations. Fourier stability analysis of finite element schemes for two-dimensional shallow-water equations is presented in Anmala and Mohtar (2011). A Fourier analysis is used to study the phase and group speeds of the linearised two-dimensional shallow-water equations in a non-orthogonal boundary-fitted coordinate system in Sankaranarayanan and Spaulding (2003). We leave a Fourier stability analysis of the presented discretised equations in the current paper for further study.

### Numerical simulation with an initial non-zero potential vorticity field

We first compare the presented *fourth-order C-bracket sloshing integrator* – fourth-order in the potential vorticity dependent part of the Poisson bracket – against the *second-order* C-bracket integrator in Alemi Ardakani and Bridges (2024) for a sloshing simulation in a rigid rectangular tank *without* inflow-outflow boundary conditions but with an initial non-zero potential vorticity field. The boundary conditions used in this simulation are the no-flow boundary conditions (3.3), and the boundary conditions (3.38) and (3.45) to enforce conservation of the Casimir property in (3.1), which give the zero-potential-vorticity conditions in (3.40) and (3.47). Set $q_1(t)=q_2(t)=0$, $h_b\left( x,y\right) =0$, ν=0 (dissipation/diffusion coefficient), and take an initial wave profile of the form

4.1 $$\begin{aligned} h\left( x,y,0\right) = h_0+\varepsilon \cos \lambda _mx\cos \beta _ny \,, \end{aligned}$$

but with a non-trivial velocity field given by

4.2 $$\begin{aligned} \begin{array}{rcl} u\left( x,y,0\right) & =& \displaystyle \varepsilon \sin \lambda _mx\cos \beta _ny \,,\\ v\left( x,y,0\right) & =& \displaystyle -\varepsilon \,\frac{\lambda _m}{\beta _n} \cos \lambda _mx\sin \beta _ny \,, \end{array} \end{aligned}$$

where ε is an input amplitude, $\lambda _m=m\pi /L_1$, $\beta _n=n\pi /L_2$, and *m*, *n* are positive integers (see §4 in Alemi Ardakani and Bridges (2024)). This velocity field is divergence-free and has vertical vorticity field

4.3 $$\begin{aligned} \big (v_x-u_y\big )\bigg |_{t=0} = \varepsilon \left( \frac{\lambda _m^2+\beta _n^2}{\beta _n}\right) \sin \lambda _mx\sin \beta _ny \,. \end{aligned}$$

Moreover, the velocity field (4.2) satisfies the zero-potential-vorticity boundary conditions (3.40) and (3.47). For this simulation, take the input parameters:

4.4 $$\begin{aligned} \begin{array}{rcl} L_1 & =& 1.0\,m\,,\,\, L_2=1.0\,m\,,\,\, d=0.01\,m\,,\,\, \Delta t=5\times 10^{-3}\,s\,,\\ m & =& n=1\,,\,\, \varepsilon = 0.03\,,\,\, h_0=0.15\,m \,. \end{array} \end{aligned}$$

**Fig. 6:**
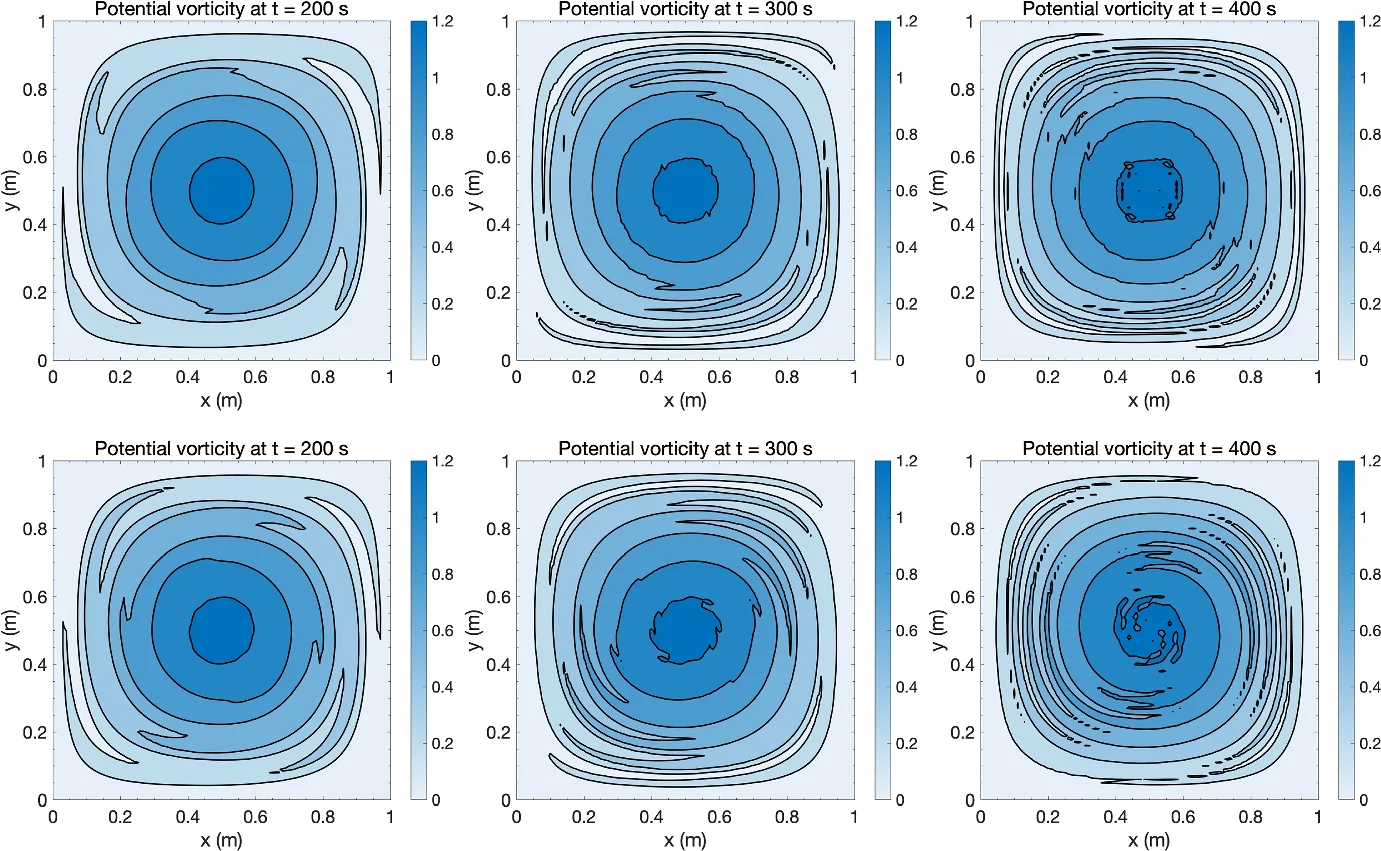
The fourth-order C-bracket sloshing solver presented in this paper against the second-order C-bracket sloshing solver of Alemi Ardakani and Bridges (2024). First row, the computed potential-vorticity-field on a 100×100 grid at t=200s, t=300s and t=400s using the *fourth-order* C-bracket sloshing solver presented in this paper. Second row, the computed potential-vorticity-field on a 100×100 grid at t=200s, t=300s and t=400s using the *second-order* C-bracket solver of Alemi Ardakani and Bridges (2024). The potential vorticity is calculated using (2.9b).

Using the fourth-order C-bracket solver, the snapshots of the potential vorticity field on a 100×100 grid at t=200s, t=300s and t=400s are depicted in Fig. 6 against the second-order C-bracket solver in Alemi Ardakani and Bridges (2024). As shown in these plots the solutions are similar, but the potential vorticity field is less perturbed (or distorted) in the fourth-order solutions, especially at the centre of the domain of solution. The potential vorticity fields are less similar at later times. This numerical test shows that the fourth-order scheme provides high-resolution solutions, which is desirable for more complex modelling problems such as *long-time* sloshing simulations with an external forcing function, a variable bottom surface, and inflow-outflow boundary conditions.

The exact conservation of the discrete Hamiltonian *H* in (2.1) and potential enstrophy *P* in (2.8) only holds in the semi-discrete formulation in which the values of *u*, *v*, *h* at grid points evolve according to an ODE system in continuous time. The fully discrete temporal integrator does not conserve energy or potential enstrophy at the level of machine precision but, rather, the errors fluctuate around long term means. Developing a symplectic time integrator is an open question for future research.

**Fig. 7:**
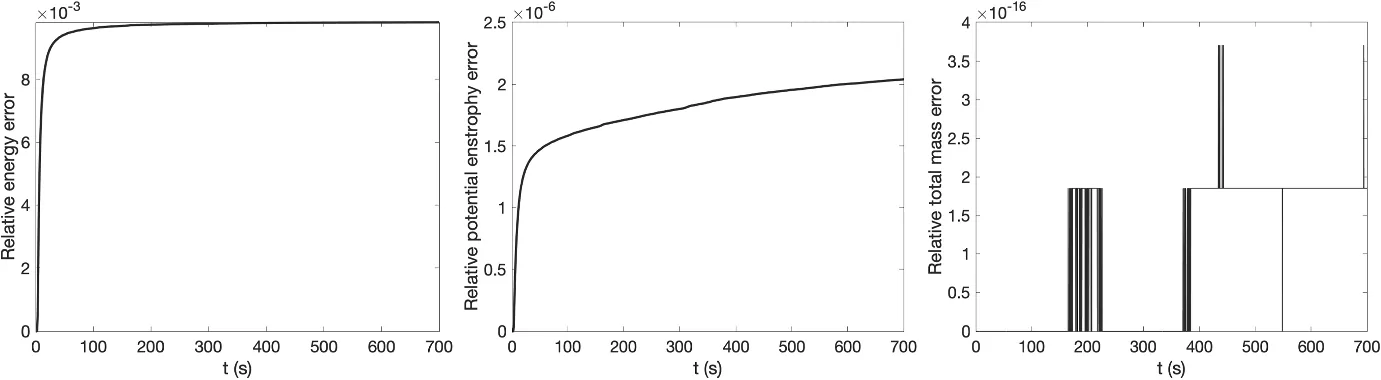
From left to right, the *relative* energy, potential enstrophy, and total mass errors plotted for 700s for the simulation in Fig. 6. The vertical axis for the domain-summed energy error is $|H\left( t\right) -H\left( 0\right) |/|H\left( 0\right) |$, where the discrete Hamiltonian *H* is given in (2.1). The vertical axis for the domain-summed potential enstrophy error is $|P\left( t\right) -P\left( 0\right) |/|P\left( 0\right) |$, where *P* is given in (2.8). The vertical axis for the domain-summed relative mass error is $|M\left( t\right) -M\left( 0\right) |/|M\left( 0\right) |$, where *M* is given in (2.10). The Knuth summation algorithm is used to calculate these errors.

For the simulation in this section, the *relative* discrete energy, potential enstrophy, and total mass errors for 700s are presented in Fig. 7. The domain-summed total mass is conserved to machine precision. The domain-summed total energy and potential enstrophy absolute errors are not fourth-order accurate using the fourth-order Runge–Kutta time-integrator. This might be due to the boundary conditions (3.38) and (3.45), i.e. the imposed zero-potential-vorticity boundary conditions (3.40) and (3.47), used to enforce conservation of the Casimir property in (3.1) *in the presence of boundaries*. But the errors remain bounded over long-time integration.

**Fig. 8:**
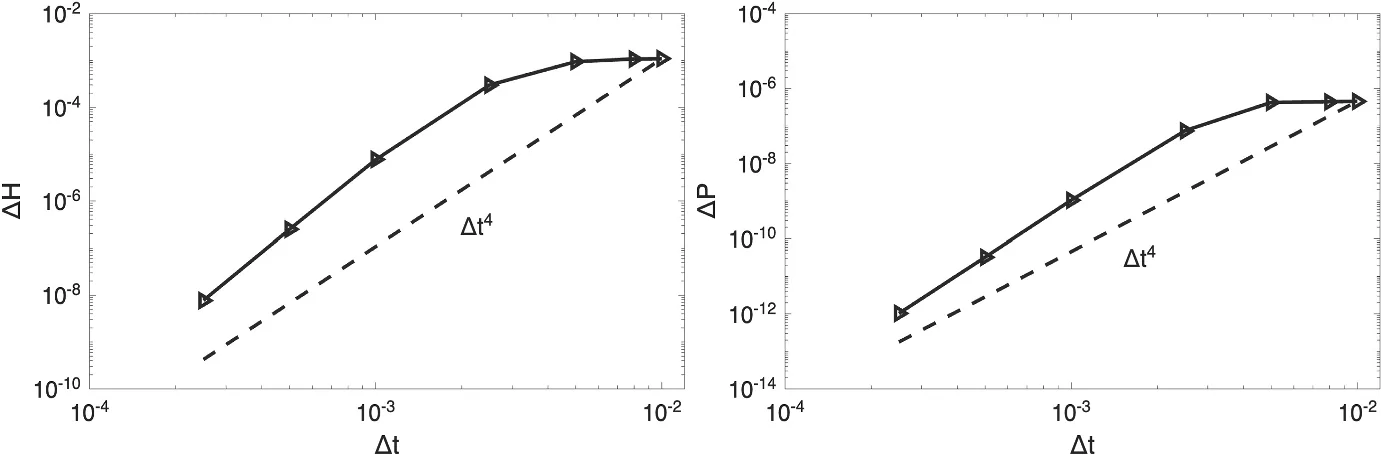
On the left, the $\log -\log$ plot for change in the domain-summed energy (Hamiltonian) between t=0s and t=500s, i.e. ΔH=H(500)-H(0) (the absolute energy error), for the simulation in Fig. 6, on a coarse 50×50 grid. The discrete Hamiltonian *H* is given in (2.1). On the right, the $\log -\log$ plot for change in the domain-summed potential enstrophy between t=0s and t=500s, i.e. ΔP=P(500)-P(0) (the absolute potential enstrophy error), for the simulation in Fig. 6, on a coarse 50×50 grid. The discrete potential enstrophy *P* is given in (2.8). The Knuth summation algorithm is used to calculate these errors.

Figure 8 shows the $\log -\log$ plot of the changes in the instantaneous domain-summed total energy and potential enstrophy (absolute errors) on a coarse 50×50 grid between t=0s and t=500s as a function of the time step Δt using the classical fourth-order Runge–Kutta scheme. The energy error reduces with $\mathcal {O}\left( \Delta t^p\right)$ with p≈3.21, and the potential enstrophy error reduces with $\mathcal {O}\left( \Delta t^p\right)$ with p≈3.52, consistent with with the use of RK4 time-stepping — the expected errors of $\mathcal {O}\left( \Delta t^4\right)$ are also depicted in Fig. 8 for comparison. We have used the Knuth summation algorithm (see e.g. (Knuth 1998; Blanchard et al. 2020)) to compute these errors, which compensates for the residual lost when adding a smaller quantity to a larger quantity.

We note that the antisymmetric shallow water Poisson-bracket (1.15) is used to design the numerical algorithm. We anticipate that if one designs a structure-preserving numerical algorithm based on the bracket (1.14), i.e. including the boundary integral term, then the errors might be closer to $\mathcal {O}\left( \Delta t^4\right)$ using the fourth-order Runge–Kutta scheme.

### Numerical solutions with an initial non-zero potential vorticity field, a prescribed diagonal forcing function near resonance, and a corrugated bottom surface

To further test the long-time modelling properties of the proposed fourth-order C-bracket solver, with biharmonic dissipation, for shallow-water sloshing over a *corrugated bottom surface* in a rigid rectangular tank undergoing a *prescribed diagonal forcing function near resonance*, we reproduce *swirling waves* reported in §5.1.3 of Alemi Ardakani and Bridges (2024), but over a *corrugated* bottom surface and with an injected *initial non-zero potential vorticity field*.

Swirling waves are the sloshing waves that move along the tank walls in a clockwise or counterclockwise direction. Swirling waves are found when the surge and sway excitation frequencies are close to the first natural frequency $\omega _{10}$ (see §4 in Alemi Ardakani and Bridges (2024) for the linearised solutions of the SWEs). Swirling waves are reported in the literature for sloshing over a flat bottom topography (Faltinsen et al. 2003; Wu and Chen 2009; Alemi Ardakani and Bridges 2024), but in this numerical experiment they are observed over a variable bottom surface as well. By taking the surge and sway forcing functions to be out of phase, a clear example of swirling wave emerges. Consider the case of diagonal forcing with the surge and sway $90^{\circ }$ out of phase:

4.5 $$\begin{aligned} q_1\left( t\right) = \varepsilon _x\cos \omega _xt \quad \text{ and }\quad q_2\left( t\right) = \varepsilon _y\sin \omega _yt \,. \end{aligned}$$

The forcing function parameters are taken to be

4.6 $$\begin{aligned} \varepsilon _x=\varepsilon _y=0.0005\,m \quad \text{ and }\quad \omega _x=\omega _y=1.03\,\omega _{10}=3.9252\,rad\,s^{-1}\,, \end{aligned}$$

where $\omega _{mn}=\alpha _{mn}\sqrt{gh_0}$ and $\alpha _{mn}=\sqrt{\lambda _m^2+\beta _n^2}$ ($\lambda _m$ and $\beta _n$ are defined in §4.1) with m=1 and n=0 in (4.6). For this simulation the corrugated bottom surface takes the form

4.7 $$\begin{aligned} h_b\left( x,y\right) = 0.05+0.03\cos \left( 8\pi x\right) \cos \left( 8\pi y\right) \,. \end{aligned}$$

Take the same initial conditions (4.2), (4.3), and the numerical parameters in (4.4), but set m=n=2. The initial wave profile takes a distribution of the form

4.8 $$\begin{aligned} h\left( x,y,0\right) = h_0+\varepsilon \cos \lambda _mx\cos \beta _ny+0.05-h_b\left( x,y\right) \,. \end{aligned}$$

The boundary conditions used for the wave height are (3.43), (3.44), (3.50) and (3.51).

**Fig. 9:**
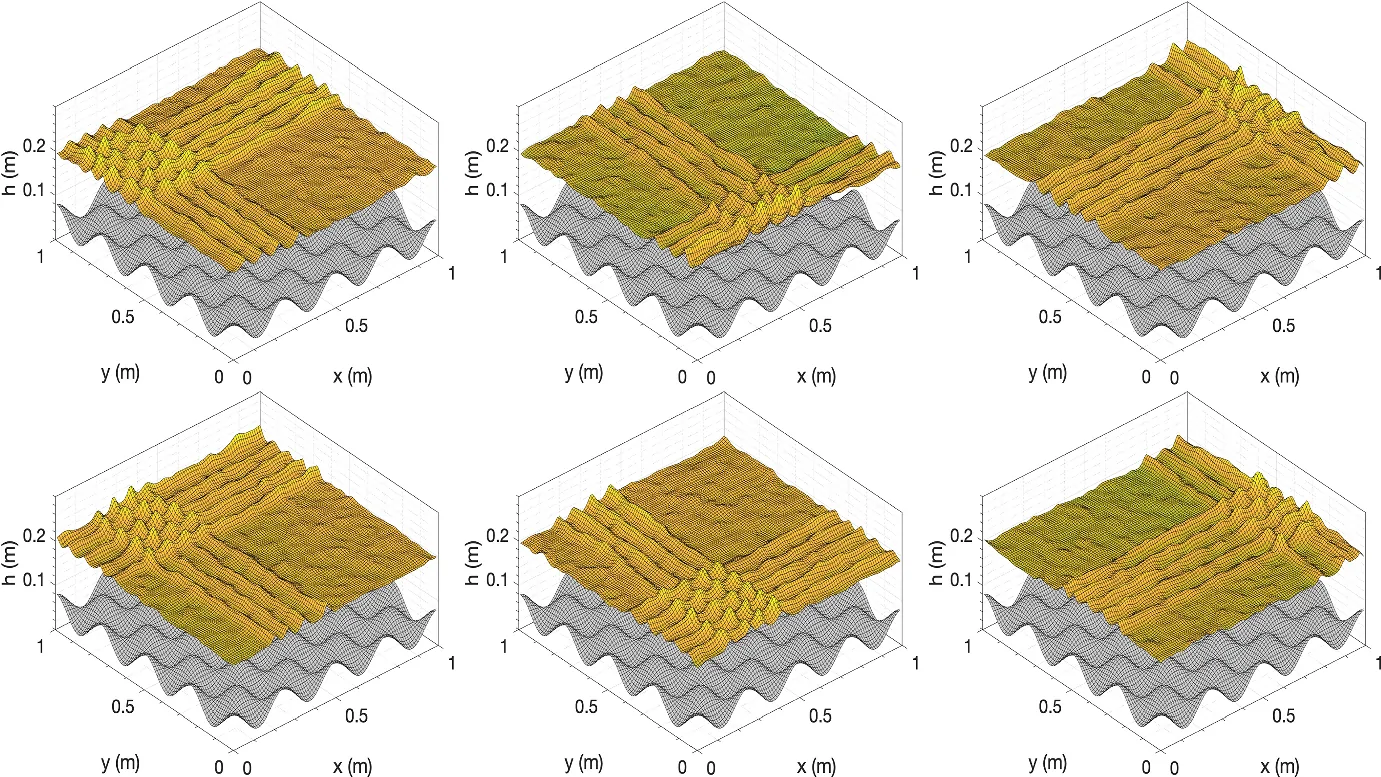
Snapshots showing the formation of a *counterclockwise swirling wave* over a *corrugated bottom surface* (4.7) in a square-base basin undergoing a prescribed diagonal motion (4.5) near *resonance*. First row, from left to right, the free-surface profile at t=750s, the free-surface profile at t=750.5s, and the free-surface profile at t=751s. Second row, from left to right, the free-surface profile at t=751.5s, the free-surface profile at t=752s, and the free-surface profile at t=752.5s.

We first present numerical solutions without biharmonic dissipation. Figure 9 shows the propagation of a *counterclockwise swirling wave* along the tank walls over a *corrugated bottom surface* at a sequence of times. In the presence of a corrugated bottom surface some small scale dispersive waves are observed on the free surface profiles. See also Fig. 7 in Alemi Ardakani and Bridges (2024) for the formation of swirling waves over a *flat bottom surface*.

**Fig. 10:**
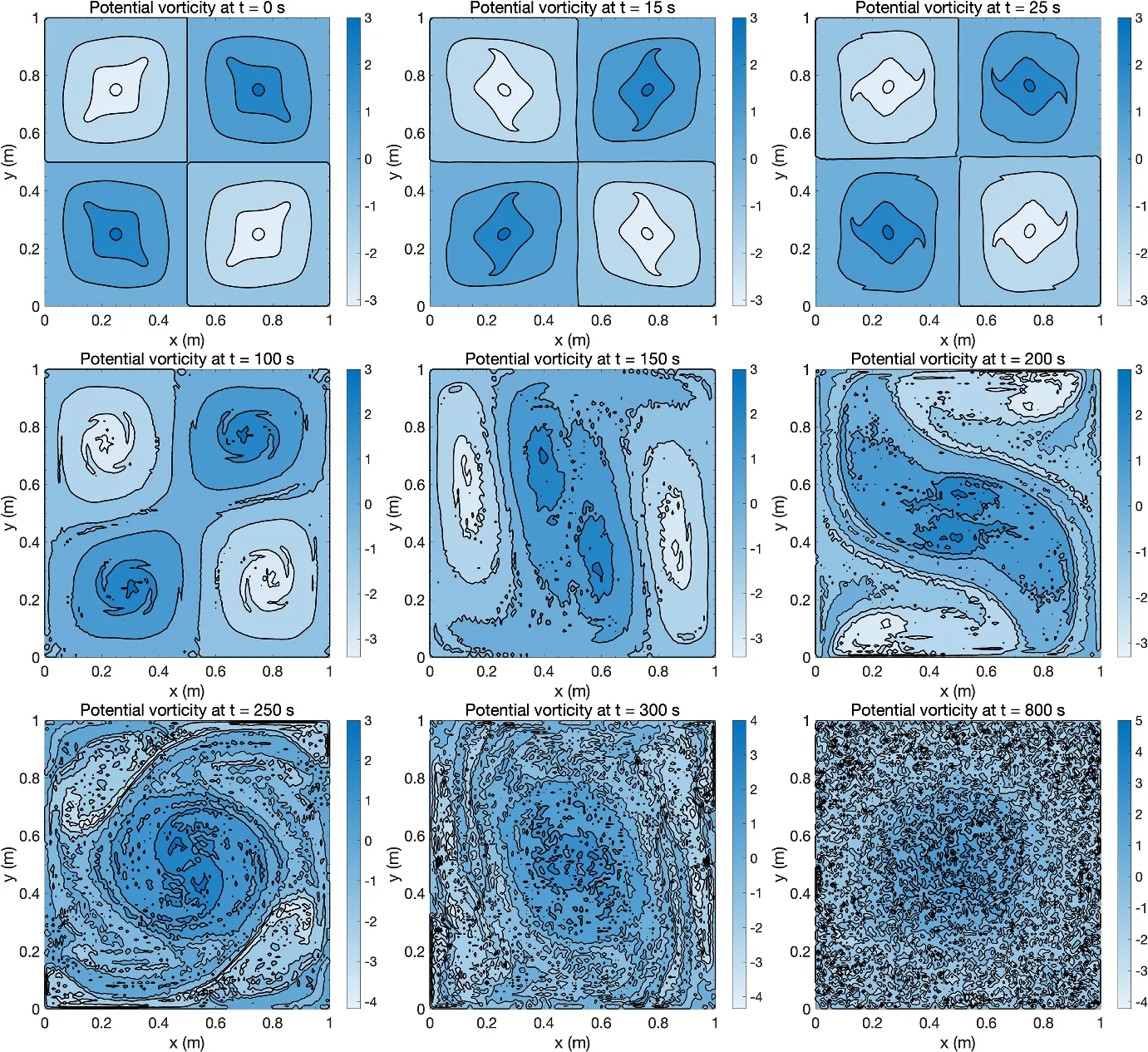
The potential vorticity field with *no dissipation* for the swirling wave simulation in Fig. 9. The paired vortices interact over time in a sloshing system, stretch, merge and mix at later times. First row, the initial potential vorticity field at t=0s, and the computed potential-vorticity-field at t=15s and t=25s using the *fourth-order* C-bracket sloshing solver. Second row, the computed potential-vorticity-field at t=100s, t=150s and t=200s. Third row, the computed potential vorticity field at t=250s, t=300s and t=800s. The potential vorticity is calculated using (2.9b). The potential vorticity field with no dissipation becomes noisy at later times.

Snapshots of the potential vorticity field at t=0s, t=15s, t=25s, t=100s, t=150s, t=200s, t=250s, t=300s, and t=800s are depicted in Fig. 10. As the paired vortices interact at later times, the Froude number $Fr=\sqrt{\boldsymbol{u}\boldsymbol{\cdot }\boldsymbol{u}/gh}$ increases and the vortices become stronger. As shown in these plots, the vortices stretch, merge and mix at later times. Due to the turbulent cascade of potential enstrophy to small scales, noise will eventually develop unless some form of potential enstrophy dissipation is introduced. As depicted in Fig. 10, the potential vorticity field, with no dissipation, becomes noisy at later times, e.g., see the potential vorticity field at t=300s and t=800s.

#### Simulation with biharmonic dissipation

**Fig. 11:**
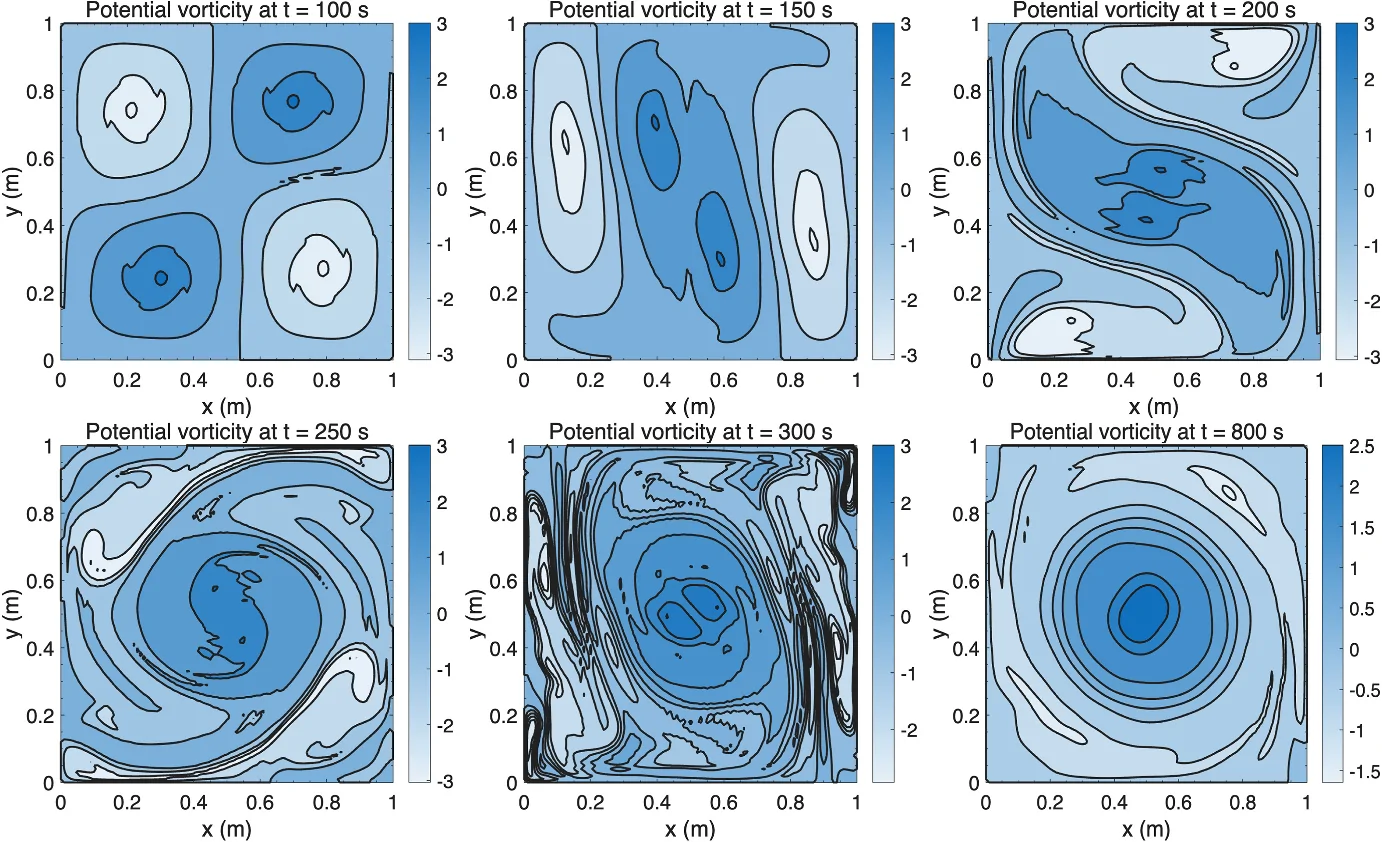
The potential vorticity field with *biharmonic dissipation* for the swirling wave simulation in Fig. 9. First row, the computed potential-vorticity-field at t=100s, t=150s and t=200s using the *fourth-order* C-bracket discretisation with the dissipation terms (2.22) and (2.23). Second row, the computed potential vorticity field at t=250s, t=300s and t=800s. Compared with the simulation in Fig. 10, the biharmonic dissipation simulation dissipates small-scale motion to better preserve the coherent structures of the solutions and stabilise the fields over long-time integration.

Next, we incorporate the biharmonic dissipation terms, as introduced in §2.1, into the fourth-order C-bracket discretisation to dissipate enstrophy at small scales. This significantly improves the quality of the approximate solutions, enabling long-term integrations to be carried out. Set the dissipation coefficient in the discretisations (2.22) and (2.23) to $\nu =10^{-10}\,m^4/s$. Snapshots of the potential vorticity field at t=100s, t=150s, t=200s, t=250s, t=300s, and t=800s are depicted in Fig. 11. Compared with the simulation in Fig. 10 (with no dissipation), the biharmonic dissipation simulation dissipates small-scale motion to better preserve the coherent structures of the solutions and stabilise the fields over long-time integration.

The total mass is conserved to machine precision for both simulations with no dissipation and with biharmonic dissipation. The domain-summed potential enstrophy and total energy are approximately conserved to $\mathcal {O}\left( \Delta t^p\right)$ with 1<p<2 for the simulation with no dissipation, and the errors remain bounded over long-time simulation. Interestingly, for the simulation with biharmonic dissipation, the domain-summed energy is conserved to the same order as the simulation without dissipation. However, as expected, the domain-summed potential enstrophy dissipates over long-time integration. We also note that the biharmonic dissipation simulation recovers the swirling wave surface profiles as depicted in Fig. 9. This shows that incorporation of biharmonic dissipation into the C-bracket scheme does not interfere with long-time sloshing dynamics.

This numerical experiment shows that the fourth-order C-bracket scheme with biharmonic dissipation and the sloshing boundary conditions (3.3), (3.38) and (3.45) is a robust integrator which can be used for long-time simulation of shallow-water sloshing problems in complex setups like the FlexSlosh WEC.

### Numerical simulation with symmetric porous side walls and periodic inflow-outflow boundary conditions as proposed in §3.1

For the next simulation, in addition to a prescribed diagonal forcing function, a corrugated bottom surface, and an initial potential vorticity field, we include *periodic inflow-outflow boundary conditions* as proposed in §3.1 in our modelling, which is applicable to the sloshing problem in the FlexSlosh WEC.

Diagonal wave sloshing in a tank was first investigated by Miles (1994). A diagonal wave corresponds to a nearly diagonal standing wave. In the *shallow-water limit*, diagonal waves are observed in a sloshing tank with a *flat* bottom surface in Alemi Ardakani and Bridges (2024) (see §5.1.2 in Alemi Ardakani and Bridges (2024)). Diagonal waves are also reported in Faltinsen et al. (2003); Wu and Chen (2009) in a sloshing tank with a flat bottom surface and interior *finite water depth*. Our numerical simulations in this section show that diagonal waves can also be observed in a sloshing tank with a *variable bottom surface* with *influx/efflux boundary conditions*.

For this simulation the prescribed surge $q_1(t)$ and sway $q_2(t)$ motions of the square-base rigid tank are taken to be harmonic and in phase:

4.9 $$\begin{aligned} q_1\left( t\right) = \varepsilon _x\cos \omega _xt \quad \text{ and }\quad q_2\left( t\right) = \varepsilon _y\cos \omega _yt\,. \end{aligned}$$

The excitation frequencies and amplitudes are

4.10 $$\begin{aligned} \omega _x=\omega _y=0.9\,\omega _{10}=3.4298\,rad\,s^{-1} \quad \text{ and }\quad \varepsilon _x=\varepsilon _y=0.005\,m\,. \end{aligned}$$

The corrugated bottom surface takes the form

4.11 $$\begin{aligned} h_b\left( x,y\right) = 0.05+0.03\cos \left( 5\pi x\right) \cos \left( 3\pi y\right) \,. \end{aligned}$$

Take the same initial conditions (4.2), (4.3), and the numerical parameters in (4.4), but set m=n=3. The initial fluid depth, with a flat free surface, takes the form

4.12 $$\begin{aligned} h\left( x,y,0\right) = h_0+0.05-h_b\left( x,y\right) \,. \end{aligned}$$

Consider three inlets/outlets of width *d* along the *x*-direction boundaries (see Fig. 3), and four inlets/outlets of width *d* along the *y*-direction boundaries. The prescribed velocity $u_k$ at the symmetric inlets/outlets along the *x*-direction boundaries (see Fig. 3) takes the form

4.13 $$\begin{aligned} \begin{array}{rcl} u_k\left( 0,y_k,t\right) =u_k\left( L_1,y_k,t\right) & =& \epsilon _k\left| \cos \omega _kt\right| \,,\quad k=1,2,3\,, \\ \left( y_1,y_2,y_3\right) & =& \left( 0.295,0.495,0.695\right) \,m \,, \end{array} \end{aligned}$$

with

4.14 $$\begin{aligned} \begin{array}{rcl} \left( \epsilon _1,\epsilon _2,\epsilon _3\right) & =& \left( 0.003,-0.002,0.003\right) m\,s^{-1}\,,\,\, \left( \omega _1,\omega _2,\omega _3\right) = \left( 0.9\,\omega _{10},\omega _{10},0.5\,\omega _{10}\right) \,,\\ \omega _{10} & =& 3.81092\,rad\,s^{-1}\,. \end{array} \end{aligned}$$

The prescribed velocity $v_l$ at the symmetric inlets/outlets along the *y*-direction boundaries takes the form

4.15 $$\begin{aligned} \begin{array}{rcl} v_l\left( x_l,0,t\right) =v_l\left( x_l,L_2,t\right) & =& \epsilon _l^\prime \left| \cos \omega _l^\prime \right| \,,\quad l=1,2,3,4\,, \\ \left( x_1,x_2,x_3,x_4\right) & =& \left( 0.245,0.395,0.545,0.695\right) \,m \,, \end{array} \end{aligned}$$

with

4.16 $$\begin{aligned} \begin{array}{rcl} \left( \epsilon _1^\prime ,\epsilon _2^\prime ,\epsilon _3^\prime ,\epsilon _4^\prime \right) & =& \left( 0.002,-0.003,0.002,-0.003\right) m\,s^{-1}\,,\\ \left( \omega _1^\prime ,\omega _2^\prime ,\omega _3^\prime ,\omega _4^\prime \right) & =& \left( 0.8\,\omega _{10},\omega _{10},1.5\,\omega _{10},2\,\omega _{10}\right) \,. \end{array} \end{aligned}$$

We use the boundary conditions and ghost grid points values proposed in §3.1 in our fourth-order C-bracket simulation. In particular, the boundary conditions used for this simulation are (3.3), (3.38), (3.45), (3.43), (3.44), (3.50), (3.51), (3.41) and (3.48) along the solid walls, and the boundary conditions (3.17), (3.32), (3.20), (3.35), (3.24) and (3.37) for the porous parts of the boundaries. We note that the boundary conditions (3.38) and (3.45) lead to the boundary conditions (3.40) and (3.47), respectively. Moreover, the boundary conditions (3.20) and (3.35) lead to the boundary conditions (3.18) and (3.33), respectively. With these boundary conditions, global conservation of the Casimir property in (3.1c) is satisfied.

**Fig. 12:**
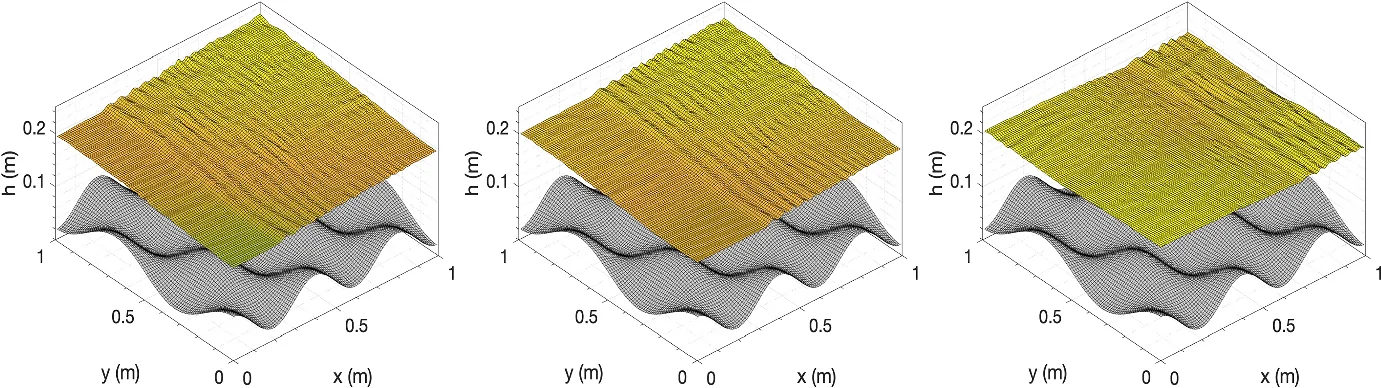
The fourth-order C-bracket sloshing solver: snapshots showing the formation of *diagonal waves* over a *corrugated bottom surface* (4.11) in a square-base basin, with *inflow-outflow mass fluxes* (4.13)-(4.15), undergoing a *prescribed diagonal motion* (4.9). From left to right, the free-surface profile at t=75s, the free-surface profile at t=150s, and the free-surface profile at t=800s.

The wave surface profiles in Fig. 12 show the formation of *diagonal waves* over a corrugated bottom surface (4.11) in a square-base basin, with inflow-outflow mass fluxes (4.13) -(4.15), undergoing a prescribed diagonal motion (4.9). In the presence of a corrugated bottom surface and inflow/outflow mass fluxes some small scale dispersive waves are observed on the free surface profiles.

**Fig. 13:**
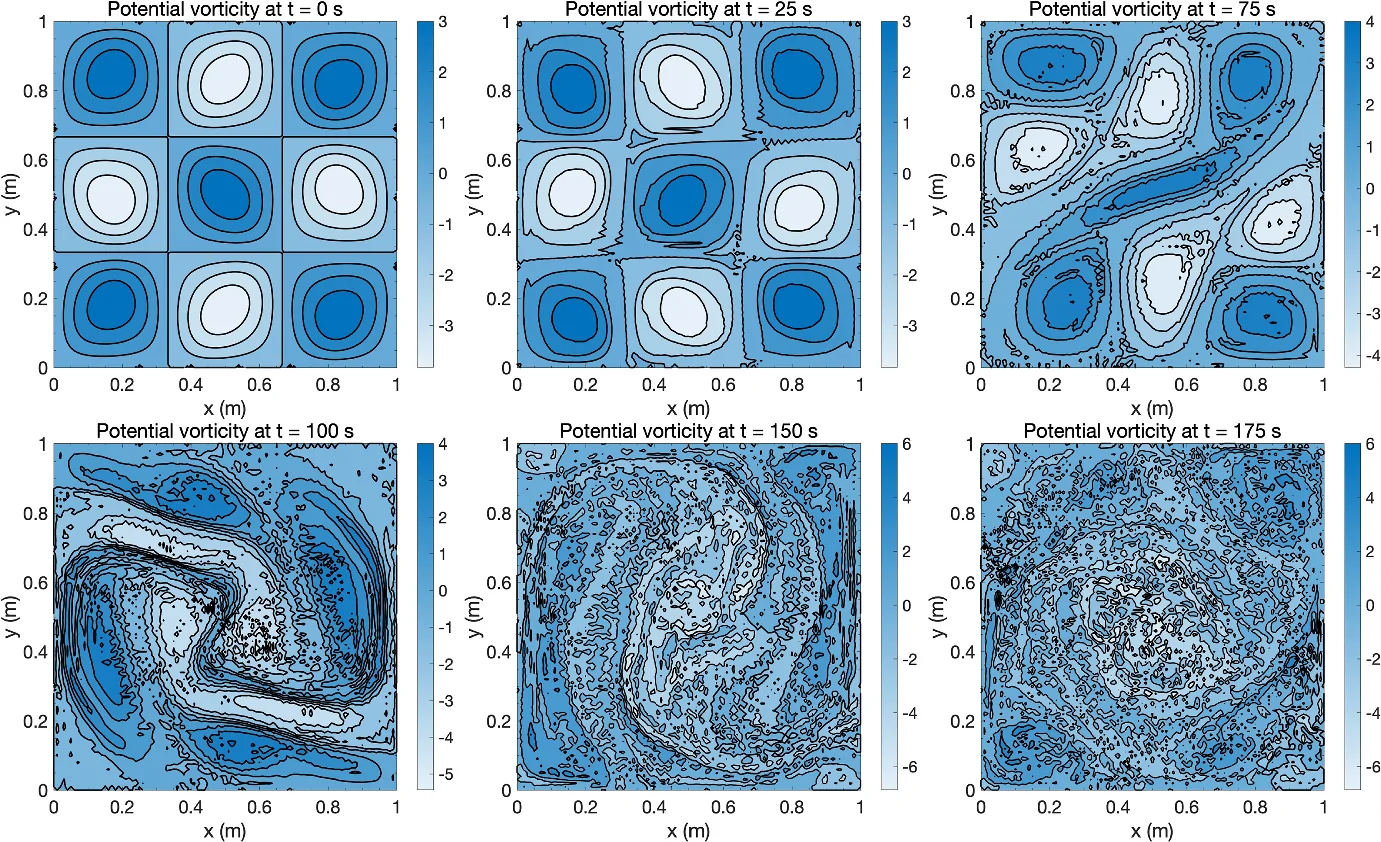
The potential vorticity field with *no dissipation* (for the diagonal wave simulation in Fig. 12) in the presence of *inflow-outflow mass fluxes*. Over time, the vortices interact, stretch, merge and mix. This leads to a chaotic (or noisy) potential vorticity field at later times. First row, the initial potential vorticity field at t=0s, and the computed potential-vorticity-field at t=25s and t=75s using the *fourth-order C-bracket sloshing solver*. Second row, the computed potential-vorticity-field at t=100s, t=150s and t=175s. The potential vorticity is calculated using (2.9b).

Snapshots of the potential vorticity field at t=0s, t=25s, t=75s, t=100s, t=150s, and t=175s are depicted in Fig. 13. As shown in these plots, the vortices interact with each other, stretch, merge and mix over time. This leads to a chaotic or noisy potential vorticity field at later times. Our numerical results (not presented here) show that the potential vorticity field in the presence of inflow-outflow mass fluxes becomes very noisy after 175s of integration.

**Fig. 14:**
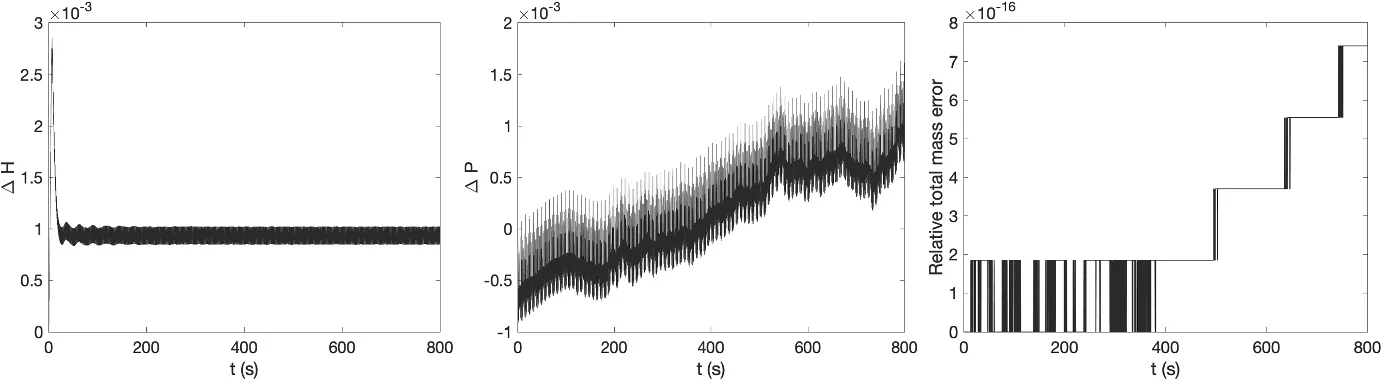
From left to right, the absolute energy and potential enstrophy errors, and the relative total mass error, plotted for 800s. The vertical axis for the domain-summed energy error is $H\left( t\right) -H\left( 0\right)$, where the discrete Hamiltonian *H* is given in (2.1). The vertical axis for the domain-summed potential enstrophy error is $P\left( t\right) -P\left( 0\right)$, where *P* is given in (2.8). The vertical axis for the domain-summed relative total mass error is $|M\left( t\right) -M\left( 0\right) |/|M\left( 0\right) |$, where *M* is given in (2.10). The Knuth summation algorithm is used to calculate these errors.

The relative total mass error and the absolute discrete energy and potential enstrophy errors for 800s are shown in Fig. 14. The domain-summed potential enstrophy is the sum of the computed potential enstrophy at the interior $\mathcal {V}$ grid points and the boundary $\mathcal {V}$ grid points. Using the above mentioned boundary conditions to preserve global conservation of the Casimir property in (3.1c), we note that potential vorticity at the boundary $\mathcal {V}$ grid points surrounding the the prescribed inflow-outflow velocities $u_k$ and $v_l$ (see Fig. 3) is non-zero, but periodic along the *x*-direction and *y*-direction porous boundaries. The domain-summed potential enstrophy and total energy are approximately conserved to $\mathcal {O}\left( \Delta t^p\right)$ with 1<p<2. As shown in these plots, the errors are bounded over 800s time integration. In particular, the potential enstrophy error remains bounded with only very small drift. The domain-summed total mass is conserved to machine precision.

#### Simulation with biharmonic dissipation

**Fig. 15:**
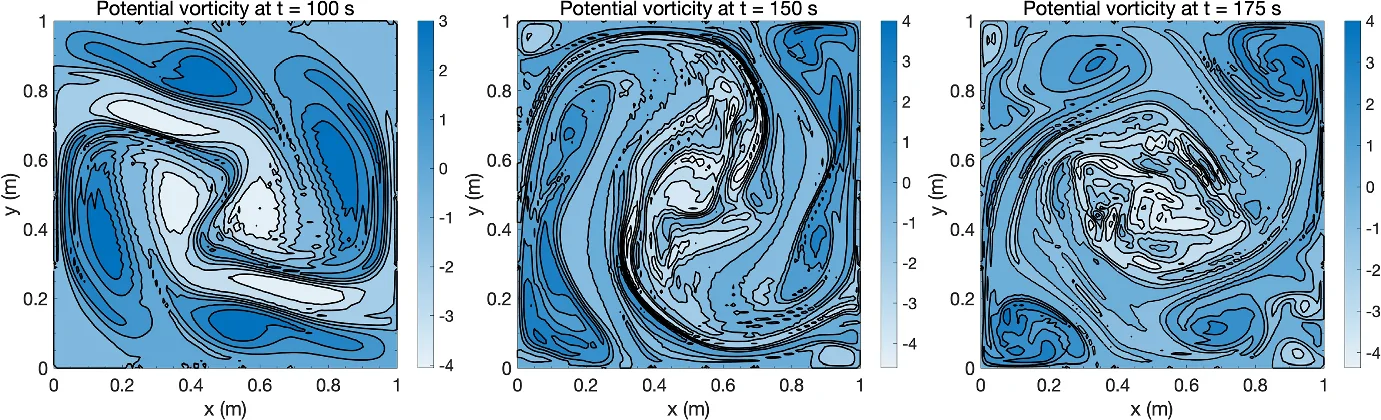
The potential vorticity field with *biharmonic dissipation* (for the diagonal wave simulation in Fig. 12) in the presence of *inflow-outflow mass fluxes*. The computed potential-vorticity-field at t=100s, t=150s and t=175s using the *fourth-order* C-bracket discretisation with the dissipation terms (2.22) and (2.23). Compared with the potential vorticity fields in Fig. 13 (see the second row), the biharmonic dissipation simulation dissipates small-scale motion to better preserve the coherent structures of the solutions and stabilise the fields over long-time integration.

Next, for the simulation in section 4.3, we incorporate the biharmonic dissipation terms into the fourth-order C-bracket discretisation to dissipate enstrophy at small scales. Set the dissipation coefficient in the discretisations (2.22) and (2.23) to $\nu =10^{-10}\,m^4/s$. Snapshots of the potential vorticity field at t=100s, t=150s, and t=175s, are depicted in Fig. 15. As shown in these plots, inclusion of biharmonic dissipation into the C-bracket scheme significantly improves the quality of the approximate solutions, enabling long-term integrations to be carried out. Compared with the potential vorticity fields in Fig. 13 (with no dissipation), the coherent structures of the solutions are better preserved over long-time integration. Interestingly, for the simulation with biharmonic dissipation, the domain-summed energy is conserved with the same accuracy as in the simulation without dissipation in Fig. 14. However, as expected, the domain-summed potential enstrophy dissipates over long-time integration. We also note that the biharmonic dissipation simulation recovers the diagonal wave surface profiles as depicted in Fig. 12. This shows that incorporation of biharmonic dissipation into the C-bracket scheme does not interfere with long-time sloshing dynamics with inflow-outflow mass fluxes.

### Numerical simulations with non-symmetric porous side walls and non-periodic boundary conditions as proposed in §3.2

The aim in this section is to test conservation properties of the fourth-order C-bracket sloshing solver for the problem of shallow-water sloshing over a corrugated bottom surface in a rigid rectangular tank, with *non-symmetric* porous side walls and *non-periodic influx-efflux boundary conditions*, undergoing prescribed surge and sway motions.

#### Repeating the numerical test in §4.1 with non-symmetric porous boundaries

We first repeat the numerical experiment in §4.1 but with non-symmetric porous boundaries and non-periodic inflow-outflow boundary conditions as proposed in Remark 3.4, which respect global conservation of the Casimir property in (3.1c). The initial conditions and input parameters remain the same as in the numerical experiment in §4.1, i.e. we take the initial wave profile in (4.1), and the initial velocity field (4.2) which induces the initial vorticity field in (4.3), with the input parameters (4.4).

Consider a computational domain with non-symmetric porous side walls as depicted in Fig. 5 with four inlets/outlets of width *d* along the bottom boundary line $\{\left( x,y\right) :\,y=0\}$ and the right boundary line $\{\left( x,y\right) :\,x=L_1\}$. The prescribed velocities $u_k$ and $v_k$ at the inlets/outlets along these boundary lines take the form

4.17 $$\begin{aligned} \begin{array}{rcl} u_k\left( L_1,y_k,t\right) =v_k\left( x_k,0,t\right) & =& \epsilon _k\left| \sin \omega _kt\right| \,,\quad k=1,2,3,4\,, \\ \left( y_1,y_2,y_3,y_4\right) & =& \left( 0.295,0.445,0.595,0.745\right) \,m \,,\\ \left( x_1,x_2,x_3,x_4\right) & =& \left( 0.245,0.395,0.545,0.695\right) \,m\,, \end{array} \end{aligned}$$

with

4.18 $$\begin{aligned} \begin{array}{rcl} \left( \epsilon _1,\epsilon _2,\epsilon _3,\epsilon _4\right) & =& \left( 0.002,-0.003,0.002,-0.003\right) m\,s^{-1}\,,\\ \left( \omega _1,\omega _2,\omega _3,\omega _4\right) & =& \left( 0.9\,\omega _{10},\omega _{10},1.5\,\omega _{10},2\,\omega _{10}\right) \,,\,\, \omega _{10}=3.81092\,rad\,s^{-1}\,. \end{array} \end{aligned}$$

The boundary conditions are (3.54), (3.55), (3.24) and (3.37) for the porous parts of the boundaries. In addition, we use the boundary condition (3.38) for all grid points along the *x*-direction boundaries, and the boundary condition (3.45) for all grid points along the *y*-direction boundaries. We note that the boundary condition (3.38) leads to the boundary conditions (3.40) and (3.41), and the boundary condition (3.45) leads to the boundary conditions (3.47) and (3.48) along the solid walls. Furthermore, we use the boundary conditions (3.43), (3.44), (3.50), and (3.51) for the *h* grid points along the solid boundaries with $\ddot{q}_1=\ddot{q}_2=0$.

**Fig. 16:**
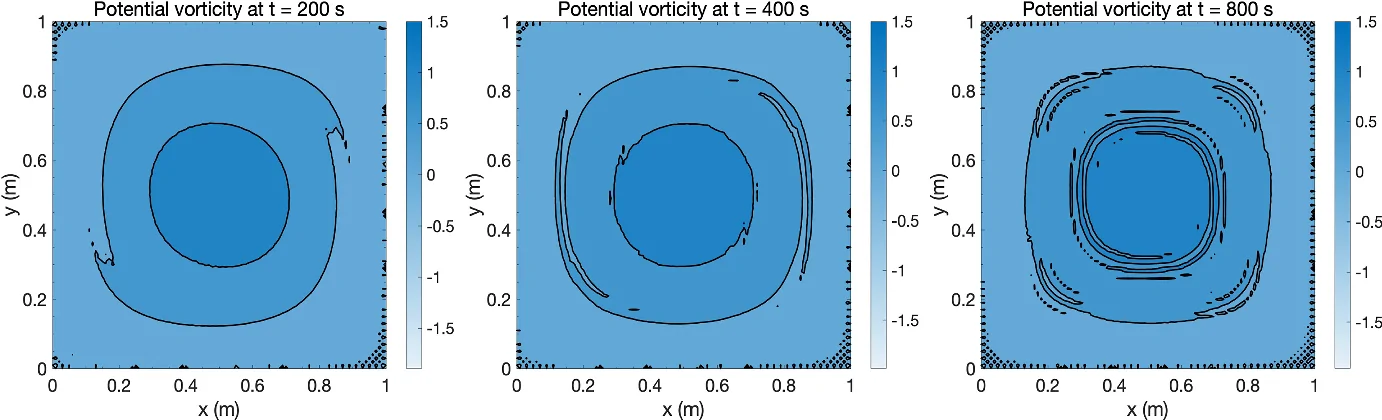
The potential vorticity field (with no dissipation) at different times. The numerical experiment in §4.1 is repeated in this simulation but with *non-symmetric porous boundaries* and *non-periodic influx-efflux boundary conditions* as proposed in Remark 3.4. The computed potential-vorticity-field at t=200s, t=400s and t=800s using the *fourth-order C-bracket sloshing solver* (without biharmonic dissipation). The potential vorticity is calculated using (2.9b).

Snapshots of the potential vorticity field up to t=800s are depicted in Fig. 16.

**Fig. 17:**
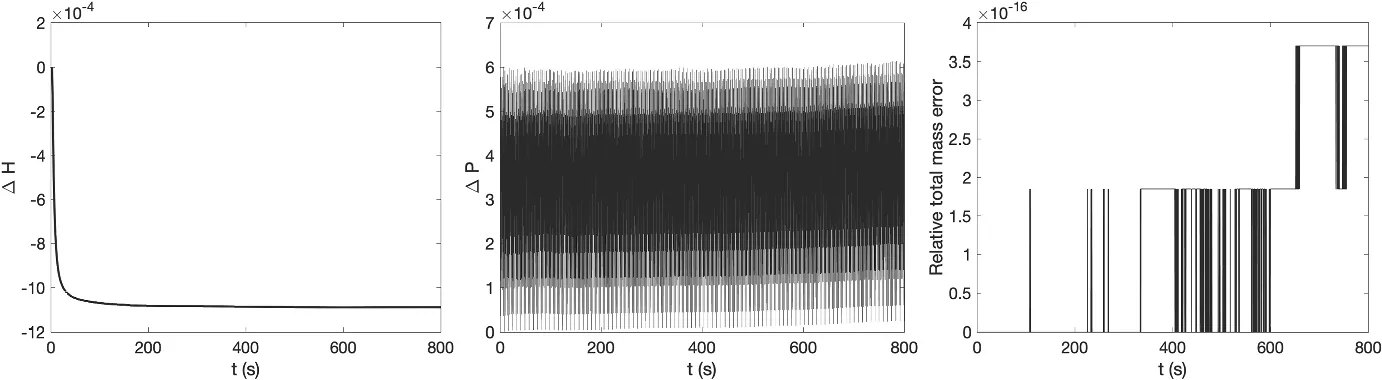
From left to right, the absolute energy and potential enstrophy errors, and the relative total mass error, plotted for 800s. The vertical axis for the domain-summed energy error is $H\left( t\right) -H\left( 0\right)$, where the discrete Hamiltonian *H* is given in (2.1). The vertical axis for the domain-summed potential enstrophy error is $P\left( t\right) -P\left( 0\right)$, where *P* is given in (2.8). The vertical axis for the domain-summed relative total mass error is $|M\left( t\right) -M\left( 0\right) |/|M\left( 0\right) |$, where *M* is given in (2.10). The Knuth summation algorithm is used to calculate these errors.

The relative total mass error and the absolute discrete energy and potential enstrophy errors for 800s are shown in Fig. 17. The domain-summed potential enstrophy is the sum of the computed potential enstrophy at the interior $\mathcal {V}$ grid points and the boundary $\mathcal {V}$ grid points. Like the previous simulation, the domain-summed potential enstrophy and total energy are approximately conserved to $\mathcal {O}\left( \Delta t^p\right)$ with 1<p<2. As shown in these plots, the errors remain bounded over 800s time integration. The domain-summed total mass is conserved to machine precision.

#### A numerical test for the SWEs with a variable bottom surface, prescribed surge-sway motion, and non-symmetric porous boundaries with non-periodic influx-efflux boundary conditions, and with an initial non-zero potential vorticity field

For the next simulation the geometry of the non-symmetric porous boundaries and inflow-outflow boundary conditions remain the same as in §4.4.1, i.e. we use (4.17) and (4.18). The boundary conditions are (3.54), (3.55), (3.24) and (3.37) for the porous parts of the boundaries. In addition, we use the boundary condition (3.38) for all grid points along the *x*-direction boundaries, and the boundary condition (3.45) for all grid points along the *y*-direction boundaries. Furthermore, we use the boundary conditions (3.43), (3.44), (3.50), and (3.51) for the *h* grid points along the solid boundaries.

**Fig. 18:**
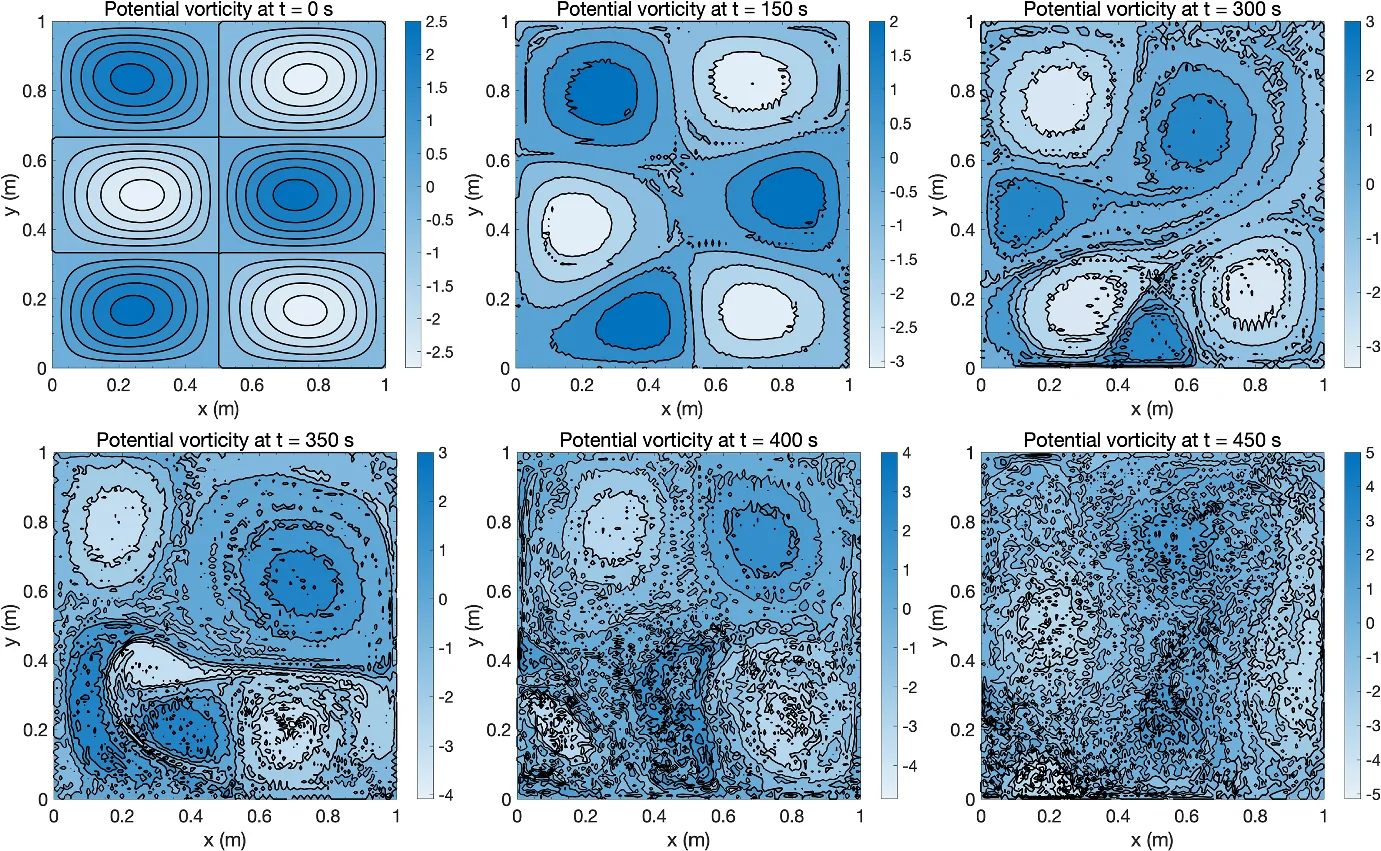
The potential vorticity field with *no dissipation* (for the square-like waves in Fig. 20) at different times in the presence of *non-symmetric porous boundaries* and *non-periodic influx-efflux boundary conditions* as proposed in Remark 3.4 with associated computational domain in Fig. 5. The tank is under a prescribed diagonal (surge-sway) motion. Over time, the vortices interact, stretch, merge and mix. This leads to a chaotic potential vorticity field at later times. First row, the initial potential vorticity field at t=0s, and the computed potential-vorticity-field at t=150s and t=300s using the *fourth-order C-bracket sloshing solver*. Second row, the computed potential-vorticity-field at t=350s, t=400s and t=450s.

A square-like wave corresponds to a nearly diagonal standing wave (see p.17 of Faltinsen et al. (2003)), where the prescribed surge $q_1(t)$ and sway $q_2(t)$ motions of the square-base rigid tank are taken to be harmonic and in phase with the forcing frequencies well above or below the lowest natural frequency. Square-like waves are reported in Faltinsen et al. (2003); Wu and Chen (2009) for fluid sloshing in a tank with interior *finite water depth*. They are also captured in numerical experiments of Alemi Ardakani and Bridges (2024) (see §5.1.1 in Alemi Ardakani and Bridges (2024)) in *shallow-water limit* for sloshing over a *flat bottom surface*. Our numerical simulations in this section show that square-like waves can also be observed in a sloshing tank with a *variable bottom surface* and with *non-periodic influx-efflux boundary conditions*.

For this simulation the prescribed surge $q_1(t)$ and sway $q_2(t)$ motions of the square-base rigid tank are taken to be harmonic and in phase as in (4.9). The excitation frequencies and amplitudes are

4.19 $$\begin{aligned} \omega _x=\omega _y=0.85\,\omega _{10}=3.2392\,rad\,s^{-1} \quad \text{ and }\quad \varepsilon _x=\varepsilon _y=0.003\,m\,. \end{aligned}$$

The variable bottom surface takes the form

4.20 $$\begin{aligned} h_b\left( x,y\right) = 0.05+0.02\cos \left( 2\pi x\right) \cos \left( 2\pi y\right) \,. \end{aligned}$$

Take the initial velocity field (4.2) which induces the initial vorticity field (4.3), and take the numerical parameters in (4.4), but set m=2 and n=3. The initial fluid depth, with a flat free surface, takes the form

4.21 $$\begin{aligned} h\left( x,y,0\right) = h_0+0.05-h_b\left( x,y\right) \,. \end{aligned}$$

Snapshots of the potential vorticity field (without biharmonic dissipation) up to t=450s are depicted in Fig. 18. Over time, the vortices interact, stretch, merge and mix. This leads to a chaotic (or noisy) potential vorticity field at later times.

**Fig. 19:**
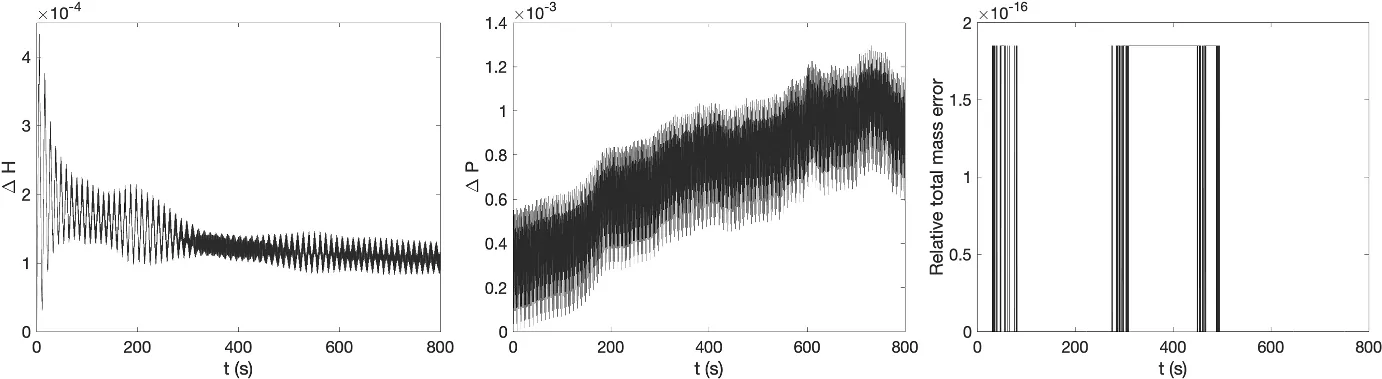
From left to right, the absolute energy and potential enstrophy errors, and the relative total mass error, plotted for 800s. The vertical axis for the domain-summed energy error is $H\left( t\right) -H\left( 0\right)$, where the discrete Hamiltonian *H* is given in (2.1). The vertical axis for the domain-summed potential enstrophy error is $P\left( t\right) -P\left( 0\right)$, where *P* is given in (2.8). The vertical axis for the domain-summed relative total mass error is $|M\left( t\right) -M\left( 0\right) |/|M\left( 0\right) |$, where *M* is given in (2.10). The Knuth summation algorithm is used to calculate these errors.

The relative total mass error and the absolute discrete energy and potential enstrophy errors for 800s are shown in Fig. 19. The domain-summed potential enstrophy is the sum of the computed potential enstrophy at the interior $\mathcal {V}$ grid points and the boundary $\mathcal {V}$ grid points. Like the previous simulation, the domain-summed potential enstrophy and total energy are approximately conserved to $\mathcal {O}\left( \Delta t^p\right)$ with 1<p<2. As shown in these plots, the errors remain bounded over 800s time integration with only very small drift. The domain-summed total mass is conserved to machine precision.

**Fig. 20:**
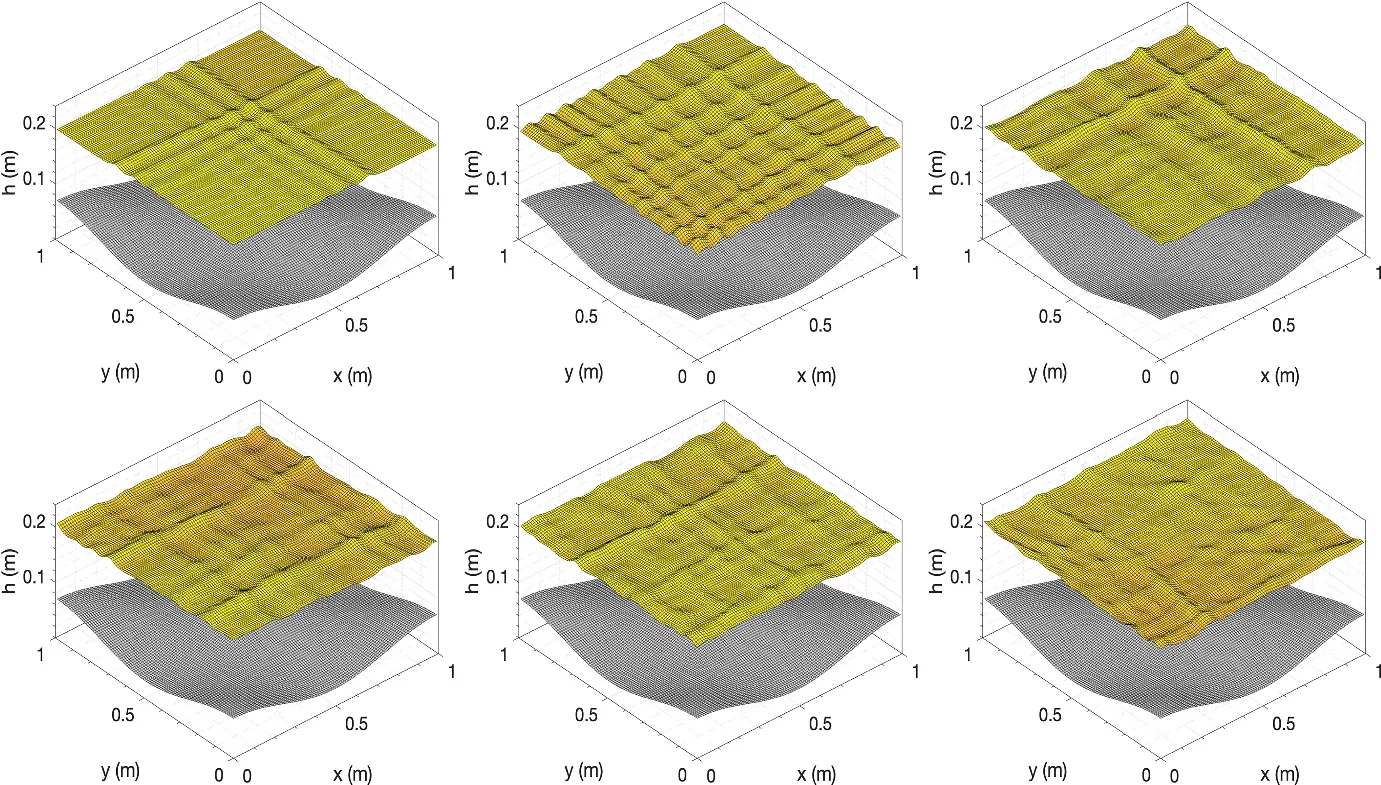
Snapshots showing the formation of *square-like waves* over a *variable bottom surface* (4.20) in a square-base basin with *non-symmetric porous side walls* and *non-periodic inflow-outflow mass fluxes* (4.17) and (4.18), undergoing a *prescribed surge-sway motion* (4.19). First row, from left to right, the free-surface profile at t=15s, t=25s, and t=75s. Second row, from left to right, the free-surface profile at t=100s, t=150s, and t=200s.

The wave surface profiles are depicted in Fig. 20 which show the formation of *square-like waves* over a variable bottom surface (4.20) in a square-base basin with *non-symmetric porous side walls* and *inflow-outflow mass fluxes* (4.17) and (4.18), undergoing a *prescribed surge-sway motion* (4.19). Square-like wave pattern is observed up to t=150s. The wave surface profile becomes irregular after 150s due to influence of the non-periodic inflow-outflow mass fluxes and bottom topography.

**Fig. 21:**
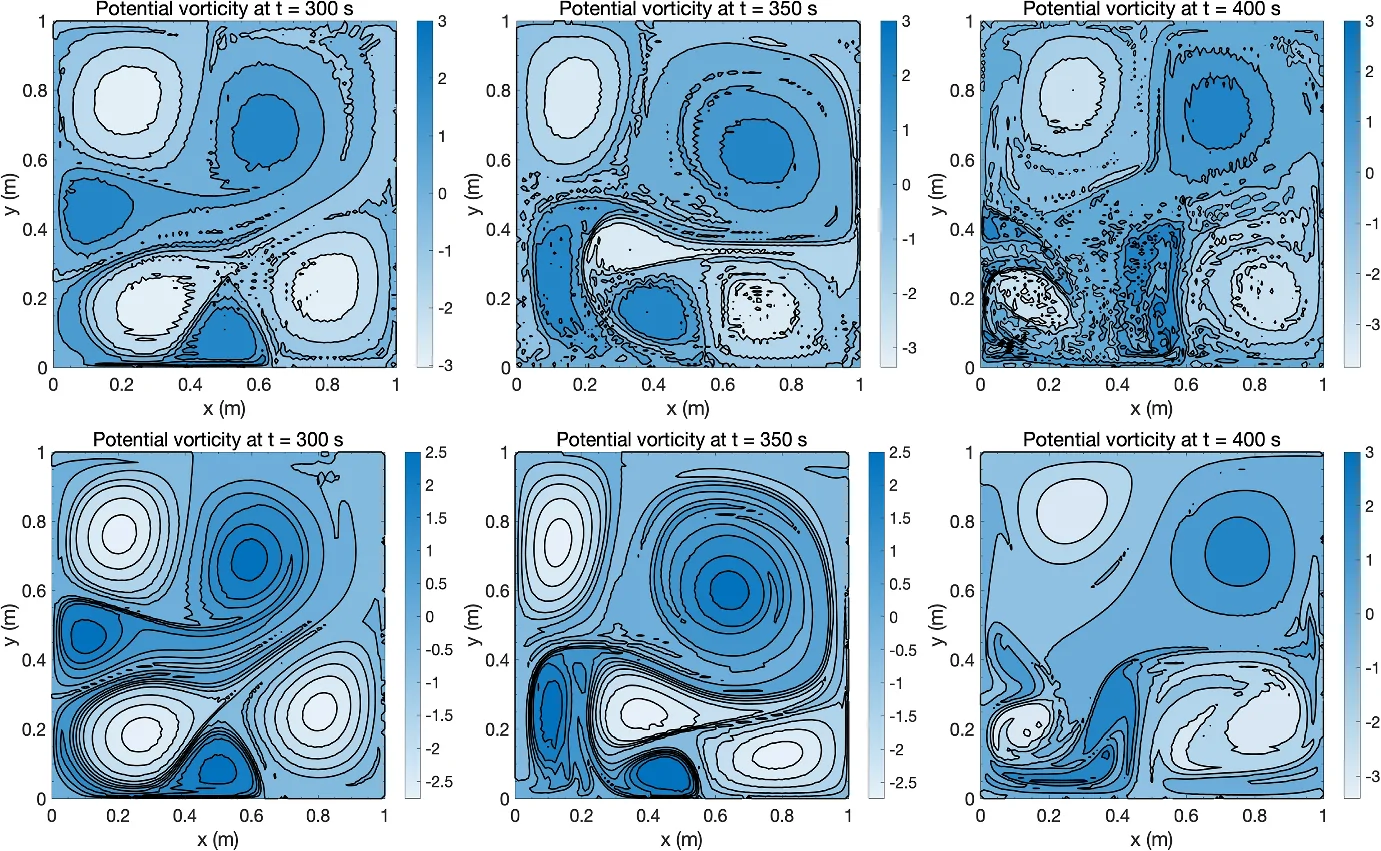
The potential vorticity field with *biharmonic dissipation* (for the square-like waves in Fig. 20) at different times in the presence of *non-symmetric porous boundaries* and *non-periodic influx-efflux boundary conditions*. First row, the computed potential-vorticity-field, with the dissipation coefficient $\nu =10^{-11}$, at t=300s, t=350s and t=400s using the *fourth-order* C-bracket discretisation including the dissipation terms (2.22) and (2.23). Second row, the computed potential-vorticity-field, with the dissipation coefficient $\nu =10^{-10}$, at t=300s, t=350s and t=400s. Compared with the potential vorticity fields in Fig. 18 (with no dissipation), the biharmonic dissipation simulation dissipates small-scale motion and improves the quality of the numerical solutions over long-time integration.

Next, we repeat this simulation including biharmonic dissipation with two different dissipation coefficients. Set the dissipation coefficient in the discretisations (2.22) and (2.23) to $\nu =10^{-11}\,m^4/s$ and $\nu =10^{-10}\,m^4/s$. Snapshots of the potential vorticity field at t=300s, t=350s, and t=400s are depicted in the first and second rows in Fig. 21, respectively. As shown in these plots, inclusion of biharmonic dissipation into the C-bracket scheme dissipates the potential enstrophy accumulation at the grid-scale, and so damps out the small scale noise, which improves the quality of the numerical solutions. For the simulation with biharmonic dissipation, the domain-summed energy is conserved with the same accuracy as in the simulation without dissipation in Fig. 19. However, as expected, the domain-summed potential enstrophy dissipates over long-time integration. We also note that the biharmonic dissipation simulation recovers the square-like wave surface profiles as depicted in Fig. 20, which shows that incorporation of biharmonic dissipation into the C-bracket scheme does not interfere with long-time sloshing dynamics with non-symmetric porous side walls and non-periodic inflow-outflow mass fluxes.

This numerical test shows that the developed fourth-order shallow-water C-bracket solver is robust, stable and precise for long-time total mass-, energy-, and potential-enstrophy-conserving numerical simulations in complex systems like the sloshing problem in the FlexSlosh WEC with different modelling components, i.e. sloshing over an irregular bottom surface with a prescribed diagonal forcing function and non-symmetric porous boundaries with non-periodic inflow-outflow boundary conditions and with an injected non-zero initial potential vorticity field.

## Concluding remarks

This paper is devoted to the development of a robust, fast, accurate and stable mass-, energy-, and potential-enstrophy-conserving numerical solver, based on a Hamiltonian functional and a non-canonical (Eulerian) Poisson-bracket operator, for long-time integration of shallow-water sloshing over a corrugated bottom surface in a rectangular rigid basin, with symmetric and non-symmetric porous boundaries and inflow-outflow mass fluxes, undergoing a prescribed diagonal (coupled surge-sway) motion. Theoretical Poisson-bracket arguments are used to obtain the required boundary conditions on the Arakawa C-grid that preserve the potential-enstrophy-conserving property of the numerical scheme in the presence of porous boundaries with the coupled sloshing and periodic/non-periodic inflow-outflow boundary conditions. The numerical scheme has fourth-order accuracy in the potential vorticity dependent component of the discrete Poisson-bracket (2.4) which leads to high-resolution numerical simulations. In addition, standard biharmonic dissipation is incorporated into the numerical model to prevent potential enstrophy from accumulating at the smallest resolved scales.

To the best of our knowledge, this is the first paper which has adapted the fourth-order Takano–Wurtele type schemes (Takano and Wurtele 1982), in the context of Poisson-bracket discretisation on the Arakawa C-grid, to the problem of nonlinear shallow-water sloshing over a variable bottom surface in an oscillating porous tank with combined no-flow and periodic/non-periodic inflow-outflow boundary conditions.

The C-bracket integrator conserves the domain-summed discrete total mass (2.10) to machine precision. The domain-summed discrete potential enstrophy (2.8) and discrete Hamiltonian (2.1) are conserved to $\mathcal {O}\left( \Delta t^p\right)$ with 1<p<2, with bounded fluctuations over long-time integration, in the numerical simulations with an initial non-zero potential vorticity field and combined no-flow and inflow-outflow boundary conditions. These conservation properties help to prevent spurious cascade of energy into high wavenumbers (Salmon 2004; Stewart and Dellar 2016), and suppress nonlinear numerical instabilities over long-time integration. In addition, as discussed above, biharmonic dissipation can be included in the numerical scheme to dissipate enstrophy at small scale and improve the quality and stability of the approximate solutions.

Using the fourth-order Runge–Kutta time integrator, one would expect to observe errors of $\mathcal {O}\left( \Delta t^4\right)$. The energy and potential enstrophy errors of $\mathcal {O}\left( \Delta t^p\right)$, with 1<p<2, might be due to the imposed sloshing and inflow-outflow boundary conditions in the context of the fourth-order C-bracket discretisation. In this paper we used the antisymmetric shallow water Poisson-bracket (1.15) to design an energy-, mass-, and potential-enstrophy-conserving numerical algorithm in the presence of boundaries with non-periodic boundary conditions. We anticipate that if one uses the bracket (1.14), which contains a boundary integral term on the right-hand side, to construct a Casimir-preserving numerical scheme, the energy and potential enstrophy errors would be closer to $\mathcal {O}\left( \Delta t^4\right)$. However, as shown in the numerical simulations in this paper the errors are bounded with only a small drift over long-time integration. Designing Casimir-preserving numerical schemes based on the bracket (1.14) (with the boundary integral term) is a future research direction of interest.

The $\log -\log$ plots of the changes in the instantaneous domain-summed total energy and potential enstrophy as a function of the time step Δt, presented in §4.1, show that the energy error reduces with $\mathcal {O}\left( \Delta t^p\right)$ with p≈3.21, and the potential enstrophy error reduces with $\mathcal {O}\left( \Delta t^p\right)$ with p≈3.52, which are consistent with the use of RK4 time-stepping.

We note that the bracket (1.14) is not antisymmetric, and so it is not a Poisson bracket. The work of Lewis et al. (1986) provides insights into construction of Poisson brackets with boundary terms. Another direction of research interest is to develop a new (antisymmetric) shallow-water Poisson-bracket with boundary terms which can be a basis for development of new structure-preserving numerical algorithms for dynamical problems with non-periodic boundary conditions.

The presented fourth-order C-bracket sloshing solver can be extended to the problem of energy extraction from shallow-water sloshing over a *flexible bottom surface* as proposed in the FlexSlosh wave energy converter [50, 49]. For this purpose, we need to extend the shallow-water Poisson-bracket formulation to a *generalised bracket* formulation for the problem of boundary coupling between the interior fluid and the bottom surface which is mechanically connected to a mesh of spring/dashpot system, which dissipates or transforms the energy of interior fluid sloshing through the deflection of the bottom surface. The main idea of the generalised bracket is that the dynamical equation for an arbitrary functional $\mathcal {F}$ of the system variables can be expressed through the master equation

5.1 $$\begin{aligned} \frac{d\mathcal {F}}{dt} = \{\mathcal {F},\mathcal {H}\}+\big [\mathcal {F},\mathcal {H}\big ] \,, \end{aligned}$$

where $\mathcal {H}$ is the Hamiltonian of the system, $\{\boldsymbol{\cdot },\boldsymbol{\cdot }\}$ describes the conservative effects through a canonical or non-canonical Poisson-bracket, and $\big [\boldsymbol{\cdot },\boldsymbol{\cdot }\big ]$ accounts for dissipative processes. For the specification of the dissipation bracket $\big [\boldsymbol{\cdot },\boldsymbol{\cdot }\big ]$ in (5.1), the rate at which the total energy (Hamiltonian) dissipates into electrical power in the PTO system will be taken into account. See (Edwards and Beris 1991; Eldred and Gay-Balmaz 2020) (and references therein) for single and double generator bracket formulation of hydrodynamics problems with irreversible processes. The presented high-resolution fourth-order C-bracket integrator has provided foundation for further structure-preserving computational modelling developments for the FlexSlosh WEC problem, which is a multicomponent dynamic coupling sloshing problem with dissipative processes.

Finally, it should be noted that the presented Hamiltonian C-bracket schemes are not capable of handling wet-dry fronts. However, this feature is not essential for the mathematical modelling of the fluid sloshing problem in the proposed FlexSlosh wave energy converter. Over the past few decades, numerous well-balanced and positivity-preserving finite-volume and finite-element schemes have been developed for the shallow-water equations with geometric source terms; see, for example, (LeVeque 1998; Leveque 2002; Bokhove 2005; Alemi Ardakani et al. 2016; Li et al. 2017) (and references therein). These methods can effectively capture shock propagation and rapidly moving wet–dry interfaces associated with wave run-up on beaches and engineered structures, as well as dam-break and flood flows.

## Data Availability

Data will be made available on request.

## Proof of the conservation of the total energy using the bracket (1.14) with the periodic or no-flow boundary conditions

The bracket (1.14) has a boundary integral which breaks its antisymmetry. It can be shown that applying appropriate boundary conditions, the conservation of the total energy (Hamiltonian) can be satisfied using the bracket (1.14). We have

A.1 $$\begin{aligned} \begin{array}{rcl} \displaystyle \{\mathcal {H}\,,\mathcal {H}\} & =& -\int _{\partial \textsf{D}}\, \frac{\delta \mathcal {H}}{\delta h}\frac{\delta \mathcal {H}}{\delta \widetilde{\boldsymbol{u}}} \boldsymbol{\cdot }d\boldsymbol{s}\\ & =& \displaystyle -\int _{\partial \textsf{D}}\,\Phi h\boldsymbol{u}\boldsymbol{\cdot }d\boldsymbol{s} \\ & =& \displaystyle \int _0^{L_2}\big [-\left( \Phi hu\right) \left( L_1,y,t\right) +\left( \Phi hu\right) \left( 0,y,t\right) \big ] \,dy \\ & & \displaystyle +\int _0^{L_1}\big [-\left( \Phi hv\right) \left( x,L_2,t\right) +\left( \Phi hv\right) \left( x,0,t\right) \big ] \,dx \end{array} \end{aligned}$$

noting that $\delta \mathcal {H}/\delta h=\Phi$ and $\delta \mathcal {H}/\delta \widetilde{\boldsymbol{u}}=\delta \mathcal {H}/\delta \boldsymbol{u}=h\boldsymbol{u}$. Now, if we use (a) periodic boundary conditions for the field variables $\left( u,v,h\right)$, and set $h_b\left( 0,y\right) =h_b\left( L_1,y\right)$ and $h_b\left( x,0\right) =h_b\left( x,L_2\right)$, or (b) use the no-flow boundary conditions in (3.3), the right-hand side in (A.1) vanishes and the Hamiltonian is conserved.

## The potential vorticity equation in bracket form

Using the bracket (1.14) with the boundary integral term on the right-hand side, the evolution equation for the potential vorticity takes the form

B.1 $$\begin{aligned} \begin{array}{rcl} \displaystyle \frac{\partial \mathcal {V}}{\partial t} & =& \displaystyle \{\mathcal {V}\,,\mathcal {H}\} \\ & =& \displaystyle \iint _{\textsf{D}}d\boldsymbol{x}\,\left[ \mathcal {V}\, \frac{\delta \left( \mathcal {V},\mathcal {H}\right) }{\delta \left( \widetilde{u},\widetilde{v}\right) } -\frac{\delta \mathcal {V}}{\delta \widetilde{\boldsymbol{u}}}\boldsymbol{\cdot }\boldsymbol{\nabla }\frac{\delta \mathcal {H}}{\delta h} +\frac{\delta \mathcal {H}}{\delta \widetilde{\boldsymbol{u}}} \boldsymbol{\cdot }\boldsymbol{\nabla }\frac{\delta \mathcal {V}}{\delta h}\right] \displaystyle -\int _{\partial \textsf{D}}\,\frac{\delta \mathcal {V}}{\delta h} \frac{\delta \mathcal {H}}{\delta \widetilde{\boldsymbol{u}}}\boldsymbol{\cdot }d\boldsymbol{s} \\ & =& \displaystyle \iint _{\textsf{D}} d\boldsymbol{x}\left[ \frac{\delta \mathcal {V}}{\delta u} \left( \mathcal {V}hv-\Phi _x\right) +\frac{\delta \mathcal {V}}{\delta v} \left( -\mathcal {V}hu-\Phi _y\right) +h\boldsymbol{u}\boldsymbol{\cdot }\boldsymbol{\nabla }\frac{\delta \mathcal {V}}{\delta h}\right] \\ & & \displaystyle -\int _{\partial \textsf{D}}\,\frac{\delta \mathcal {V}}{\delta h}\, h\boldsymbol{u}\boldsymbol{\cdot }d\boldsymbol{s}\,. \end{array} \end{aligned}$$

Now using the momentum equations in (1.2) the equation (B.1) takes the form

B.2 $$\begin{aligned} \begin{array}{rcl} \displaystyle \frac{\partial \mathcal {V}}{\partial t}= &  \displaystyle \iint _{\textsf{D}} d\boldsymbol{x}\left[ \frac{\delta \mathcal {V}}{\delta u}\, \widetilde{u}_t+\frac{\delta \mathcal {V}}{\delta v}\, \widetilde{v}_t+h\boldsymbol{u}\boldsymbol{\cdot }\boldsymbol{\nabla }\frac{\delta \mathcal {V}}{\delta h}\right] -\int _{\partial \textsf{D}}\,\frac{\delta \mathcal {V}}{\delta h}\, h\boldsymbol{u}\boldsymbol{\cdot }d\boldsymbol{s}\,. \end{array} \end{aligned}$$

On the other hand, taking the derivative of the potential vorticity with respect to time gives

B.3 $$\begin{aligned} \begin{array}{rcl} \displaystyle \frac{\partial \mathcal {V}}{\partial t}= &  \displaystyle \iint _{\textsf{D}}\,d\boldsymbol{x} \left( \frac{\delta \mathcal {V}}{\delta u}\,\widetilde{u}_t +\frac{\delta \mathcal {V}}{\delta v}\,\widetilde{v}_t +\frac{\delta \mathcal {V}}{\delta h}\,h_t\right) \,. \end{array} \end{aligned}$$

The first two terms on the right-hand side in (B.2) and (B.3) are the same. The last term in (B.3) can be written in the form

B.4 $$\begin{aligned} \begin{array}{rcl} \displaystyle \iint _{\textsf{D}}\, d\boldsymbol{x}\, \frac{\delta \mathcal {V}}{\delta h}\,h_t & =& \displaystyle -\iint _{\textsf{D}}\, d\boldsymbol{x}\,\frac{\delta \mathcal {V}}{\delta h}\, \boldsymbol{\nabla }\boldsymbol{\cdot }\left( h\boldsymbol{u}\right) \quad \text{(using } \text{ the } \text{ mass } \text{ equation } \text{ in } \text{(1.2)) } \\ & =& \displaystyle \iint _{\textsf{D}}\,d\boldsymbol{x}\,h\boldsymbol{u}\boldsymbol{\cdot }\boldsymbol{\nabla }\frac{\delta \mathcal {V}}{\delta h} -\iint _{\textsf{D}}\,\boldsymbol{\nabla }\boldsymbol{\cdot }\left( \frac{\delta \mathcal {V}}{\delta h}\,h\boldsymbol{u}\right) \\ & =& \displaystyle \iint _{\textsf{D}}\,d\boldsymbol{x}\,h\boldsymbol{u}\boldsymbol{\cdot }\boldsymbol{\nabla }\frac{\delta \mathcal {V}}{\delta h} -\int _{\partial \textsf{D}}\,\frac{\delta \mathcal {V}}{\delta h}\,h\boldsymbol{u}\boldsymbol{\cdot }d\boldsymbol{s} \,, \end{array} \end{aligned}$$

which recovers the last two terms in (B.2).

If one uses the antisymmetric shallow water Poisson-bracket (1.15), i.e. neglecting the boundary integral in (1.14), to design a potential-enstrophy-conserving C-bracket scheme in the presence of boundaries, then one should ensure that the boundary integral in the equation for the potential vorticity (B.1) vanishes using appropriate boundary conditions. The boundary integral in (B.1) takes the form

B.5 $$\begin{aligned} \begin{array}{rcl} \displaystyle \int _{\partial \textsf{D}}\frac{\delta \mathcal {V}}{\delta h}\, h\boldsymbol{u}\boldsymbol{\cdot }d\boldsymbol{s} & =& \displaystyle \int _{\partial \textsf{D}}-\mathcal {V}\boldsymbol{u}\boldsymbol{\cdot }d\boldsymbol{s} \\ & =& \displaystyle \int _0^{L_2} dy\left[ -\left( \mathcal {V}u\right) \left( L_1,y,t\right) +\left( \mathcal {V}u\right) \left( 0,y,t\right) \right] \\ & & \displaystyle +\int _0^{L_1} dx\left[ -\left( \mathcal {V}v\right) \left( x,L_2,t\right) +\left( \mathcal {V}v\right) \left( x,0,t\right) \right] \,. \end{array} \end{aligned}$$

### Remark B.1

Using the no-flow boundary conditions (3.3), or the periodic inflow-outflow boundary conditions (3.5) and (3.7), or the prescribed inflow-outflow boundary conditions (3.53) for the fluid velocity, the right-hand side in (B.5) vanishes if we use the boundary conditions (3.20) and (3.35) for symmetric porous side walls, or the boundary condition (3.55) for non-symmetric porous side walls. We note that the boundary condition (3.20) leads to the periodic boundary condition (3.18), and the boundary condition (3.35) leads to the periodic boundary condition (3.33) for the potential vorticity. The boundary condition (3.55) leads to required boundary values for the potential vorticity such that the right-hand side in (B.5) vanishes when summed over all boundary grid points along porous boundaries.

## Proof of $\{h_0,\mathcal {P}\}=0$ using the bracket (1.14)

It can be proved that $\{h_0,\mathcal {P}\}=0$ using the bracket (1.14). We have

C.1 $$\begin{aligned} \{h_0\,,\mathcal {P}\}&= \displaystyle \iint _{\textsf{D}}\, d\boldsymbol{x}\,\frac{\delta \mathcal {P}}{\delta \boldsymbol{u}}\boldsymbol{\cdot }\boldsymbol{\nabla }\frac{\delta h_0}{\delta h} -\int _{\partial \textsf{D}}\,\frac{\delta h_0}{\delta h} \frac{\delta \mathcal {P}}{\delta \boldsymbol{u}}\boldsymbol{\cdot }d\boldsymbol{s} \nonumber \\&= \displaystyle \iint _{\textsf{D}}\, d\boldsymbol{x}\,\frac{\delta \mathcal {P}}{\delta \boldsymbol{u}}\boldsymbol{\cdot }\boldsymbol{\nabla }\delta \left( \boldsymbol{x}-\boldsymbol{x}_0\right) -\int _{\partial \textsf{D}}\,\delta \left( \boldsymbol{x}-\boldsymbol{x}_0\right) \frac{\delta \mathcal {P}}{\delta \boldsymbol{u}}\boldsymbol{\cdot }d\boldsymbol{s} \nonumber \\&= \displaystyle \iint _{\textsf{D}}\, d\boldsymbol{x}\,\boldsymbol{\nabla }\boldsymbol{\cdot }\left( \frac{\delta \mathcal {P}}{\delta \boldsymbol{u}}\,\delta \left( \boldsymbol{x}-\boldsymbol{x}_0\right) \right) -\iint _{\textsf{D}}\,d\boldsymbol{x}\,\delta \left( \boldsymbol{x}-\boldsymbol{x}_0\right) \boldsymbol{\nabla }\boldsymbol{\cdot }\frac{\delta \mathcal {P}}{\delta \boldsymbol{u}}\nonumber \\&\quad -\int _{\partial \textsf{D}}\,\delta \left( \boldsymbol{x}-\boldsymbol{x}_0\right) \frac{\delta \mathcal {P}}{\delta \boldsymbol{u}}\boldsymbol{\cdot }d\boldsymbol{s} \nonumber \\&= \displaystyle \int _{\partial \textsf{D}}\,\delta \left( \boldsymbol{x}-\boldsymbol{x}_0\right) \frac{\delta \mathcal {P}}{\delta \boldsymbol{u}}\boldsymbol{\cdot }d\boldsymbol{s} -\iint _{\textsf{D}}\,d\boldsymbol{x}\,\delta \left( \boldsymbol{x}-\boldsymbol{x}_0\right) \boldsymbol{\nabla }\boldsymbol{\cdot }\frac{\delta \mathcal {P}}{\delta \boldsymbol{u}}\nonumber \\&\quad -\int _{\partial \textsf{D}}\,\delta \left( \boldsymbol{x}-\boldsymbol{x}_0\right) \frac{\delta \mathcal {P}}{\delta \boldsymbol{u}}\boldsymbol{\cdot }d\boldsymbol{s} \nonumber \\&= \displaystyle -\left( \boldsymbol{\nabla }\boldsymbol{\cdot }\frac{\delta \mathcal {P}}{\delta \boldsymbol{u}}\right) \bigg |_{\boldsymbol{x}=\boldsymbol{x}_0} \nonumber \\&= 0 \,. \end{aligned}$$

Hence, if the shallow water Poisson-bracket (1.15) is used to design a potential-enstrophy-conserving numerical algorithm, the boundary terms on the right-hand side of (3.22) must vanish imposing appropriate boundary conditions.
